# Human *Plasmodium vivax* diversity, population structure and evolutionary origin

**DOI:** 10.1371/journal.pntd.0008072

**Published:** 2020-03-09

**Authors:** Virginie Rougeron, Eric Elguero, Céline Arnathau, Beatriz Acuña Hidalgo, Patrick Durand, Sandrine Houze, Antoine Berry, Sedigheh Zakeri, Rashidul Haque, Mohammad Shafiul Alam, François Nosten, Carlo Severini, Tamirat Gebru Woldearegai, Benjamin Mordmüller, Peter Gottfried Kremsner, Lilia González-Cerón, Gustavo Fontecha, Dionicia Gamboa, Lise Musset, Eric Legrand, Oscar Noya, Tepanata Pumpaibool, Pingchai Harnyuttanakorn, Khadijetou Mint Lekweiry, Musab Mohamad Albsheer, Muzamil Mahdi Abdel Hamid, Ali Ould Mohamed Salem Boukary, Jean-François Trape, François Renaud, Franck Prugnolle

**Affiliations:** 1 Laboratoire MIVEGEC (Université de Montpellier-CNRS-IRD), CREES, Montpellier, France; 2 Service de Parasitologie-mycologie CNR du Paludisme, AP-HP Hôpital Bichat, Paris, France; 3 Centre de Physiopathologie de Toulouse-Purpan (CPTP), Institut National de la Santé et de la Recherche Médicale (INSERM) UMR1043, CNRS UMR5282, Université de Toulouse Paul Sabatier, F-31300 Toulouse, France; 4 Service de Parasitologie-Mycologie, Institut Fédératif de Biologie, Centre Hospitalier Universitaire de Toulouse, F-31300 Toulouse, France; 5 Malaria and Vector Research Group (MVRG), Biotechnology Research Center (BRC), Pasteur Institute of Iran, Tehran, Iran; 6 Emerging Infections & Parasitology Laboratory, icddr,b, Mohakhali, Dhaka, Bangladesh; 7 Centre for Tropical Medicine and Global Health,Oxford, United Kingdom; 8 Shoklo Malaria Research Unit, Mahidol-Oxford Tropical Medicine Research Unit, Faculty of Tropical Medicine, Mahidol University, Mae Sot, Thailand; 9 Department of Infectious Diseases, Istituto Superiore di Sanità, Rome, Italy; 10 Institute of Tropical Medicine, University of Tübingen, Tübingen, Germany; 11 German Centre for Infection Research (DZIF), partner site Tübingen, Tübingen, Germany; 12 Department of Medical Laboratory Sciences, College of Medical and Health Sciences, Haramaya University, Harar, Ethiopia; 13 Regional Centre of Research in Public Health, National Institute of Public Health, Tapachula, Chiapas, Mexico; 14 Microbiology Research Institute, Universidad Nacional Autónoma de Honduras, Tegucigalpa, Honduras; 15 Instituto de Medicina Tropical Alexander Von Humboldt, Universidad Peruana Cayetano Heredia, AP, Lima, Peru; 16 Unit, Institut Pasteur de Guyane, BP6010, French Guiana; 17 Malaria Genetic and Resistance Group, Biology of Host-Parasite Interactions Unit, Institut Pasteur, Paris, France; 18 Centro para Estudios Sobre Malaria, Instituto de Altos Estudios en Salud “Dr. Arnoldo Gabaldón”, Ministerio del Poder Popular para la Salud and Instituto de Medicina Tropical, Universidad Central de Venezuela, Maracay, Caracas, Venezuela; 19 Biomedical Science, Graduate School, Chulalongkorn University, Bangkok, Thailand; 20 Malaria Research Programme, College of Public Health Science, Chulalongkorn University, Bangkok, Thailand; 21 Department of Biology, Faculty of Science, Chulalongkorn University, Bangkok, Thailand; 22 UR-Génomes et milieux, Faculté des Sciences et Techniques, Université de Nouakchott Al-Aasriya, Mauritania; 23 Department of Parasitology and Medical Entomology, Medical Campus, University of Khartoum, Sudan; 24 Aix Marseille Univ, IRD, AP-HM, SSA, VITROME, Marseille, France; Deakin University, AUSTRALIA

## Abstract

More than 200 million malaria clinical cases are reported each year due to *Plasmodium vivax*, the most widespread *Plasmodium* species in the world. This species has been neglected and understudied for a long time, due to its lower mortality in comparison with *Plasmodium falciparum*. A renewed interest has emerged in the past decade with the discovery of antimalarial drug resistance and of severe and even fatal human cases. Nonetheless, today there are still significant gaps in our understanding of the population genetics and evolutionary history of *P*. *vivax*, particularly because of a lack of genetic data from Africa. To address these gaps, we genotyped 14 microsatellite loci in 834 samples obtained from 28 locations in 20 countries from around the world. We discuss the worldwide population genetic structure and diversity and the evolutionary origin of *P*. *vivax* in the world and its introduction into the Americas. This study demonstrates the importance of conducting genome-wide analyses of *P*. *vivax* in order to unravel its complex evolutionary history.

## Author summary

Among the five *Plasmodium* species infecting humans, *P*. *vivax* is the most prevalent parasite outside Africa. To date, there has been less research on this species than for *Plasmodium falciparum*, a more lethal species, principally because of the lack of an *in vitro* culture system and also because *P. vivax* is considered relatively benign. Nevertheless, *P*. *vivax* is responsible for severe and incapacitating clinical symptoms with significant effects on human health. The emergence of new drug resistance and the discovery of severe and even fatal cases due to *P*. *vivax* question the benign status of *P*. *vivax* malaria. In recent years, there has been increased interest in characterizing the distribution of genetic variation in *P*. *vivax*. However, these studies either generated genetic information from a regional geographic scale or combine genetic datasets generated in different molecular platforms, which is known to generate biased results. In this study, we used a single genotyping platform to genotype 14 microsatellite markers in 834 samples of *P*. *vivax* obtained from 28 locations in 20 countries from around the world, including several populations from East and West Africa. We discuss the worldwide population genetic structure and the evolutionary origins of *P*. *vivax*, as well as its introduction into the Americas.

## Introduction

*Plasmodium* parasites are the agents responsible for malaria, one of the worst scourges of mankind, with almost 600,000 infant deaths and about 200 million clinical cases reported each year[1]. Among the five *Plasmodium* species infecting humans, *Plasmodium vivax* is the most prevalent parasite outside Africa [2]. To date, there has been less research on this species than for *Plasmodium falciparum*, a more lethal species, mostly because of the lack of long-term *in vitro* culture system and also because it is considered rather benign compared to *P*. *falciparum*. Nevertheless, *P*. *vivax* is responsible for severe and incapacitating clinical symptoms with significant effects on human health [3,4]. Indeed, the periodicity of *P*. *vivax* transmission confers only transient and low immune protection [5] and relapsing liver stages hinder control and elimination strategies. Infected individuals present regular episodes of fever that may occur each month, leading to delays in education in infected children and to direct economic burden caused by the loss of productivity in infected adults [6]. Moreover, the continual emergence of new therapeutic resistance and the discovery of severe and even fatal cases due to *P*. *vivax* question the benign status of this malaria species [7–10]. Finally, the discovery of *P*. *vivax* parasite populations able to infect Duffy-negative reticulocytes of humans from Africa and South America [11–15] pushed the malaria community to consider *P*. *vivax* as a major public health issue.

In recent years, due to the importance of understanding the demography of populations, the migration patterns as well as the evolutionary history of the parasite, there has been increased interest in describing the distribution of genetic variation in the global *P*. *vivax* population. Several studies on *P*. *vivax* have been published, from those focusing on a small set of genetic markers to those describing population genetic structure at the scale of the entire genome (including [16–21]). Although these studies all brought their set of information, none of them was able to describe the relationship and the diversity of *P*. *vivax* populations over the entire range including East and Western African strains. Among these published studies, only one studied genetic diversity and structure of this pathogen at the worldwide scale [16]. In this study, Koepfli et al. showed that *P*. *vivax* was structured at a global geographic scale and characterized by differences in genetic diversity associated to transmission intensity [16]. This work was based on published genotyping data, associated to the genotyping by their own platform of South Asian parasite populations. To ensure the compatibility of the data produced in different laboratories, the authors genotyped a subset of each dataset using their own standardized protocol and used these results to reassign alleles where discrepancies were found. Although this is an accepted strategy, differences in sample treatment and genotyping platforms can still lead to biased results. It is therefore better to apply the same molecular strategy for all samples studied. Moreover, the study included only limited representation of the parasite isolates across Africa [16]. Indeed, among the 13 countries studied, only one African population from Sudan and nine samples from different places in Africa obtained from travellers coming back from these regions infected by *P*. *vivax* were analysed. The lack of *P*. *vivax* genetic information from the African continent is a major roadblock to a better understanding of the worldwide distribution of *P*. *vivax* genetic diversity, structure and evolutionary history. In this study, we used a single genotyping platform to genotype 14 microsatellite markers in 834 samples of *P*. *vivax* obtained from 28 locations in 20 countries from around the world, including several populations from East and West Africa.

## Methods

### Study sites and *P*. *vivax* isolates collection

**Fig 1:**
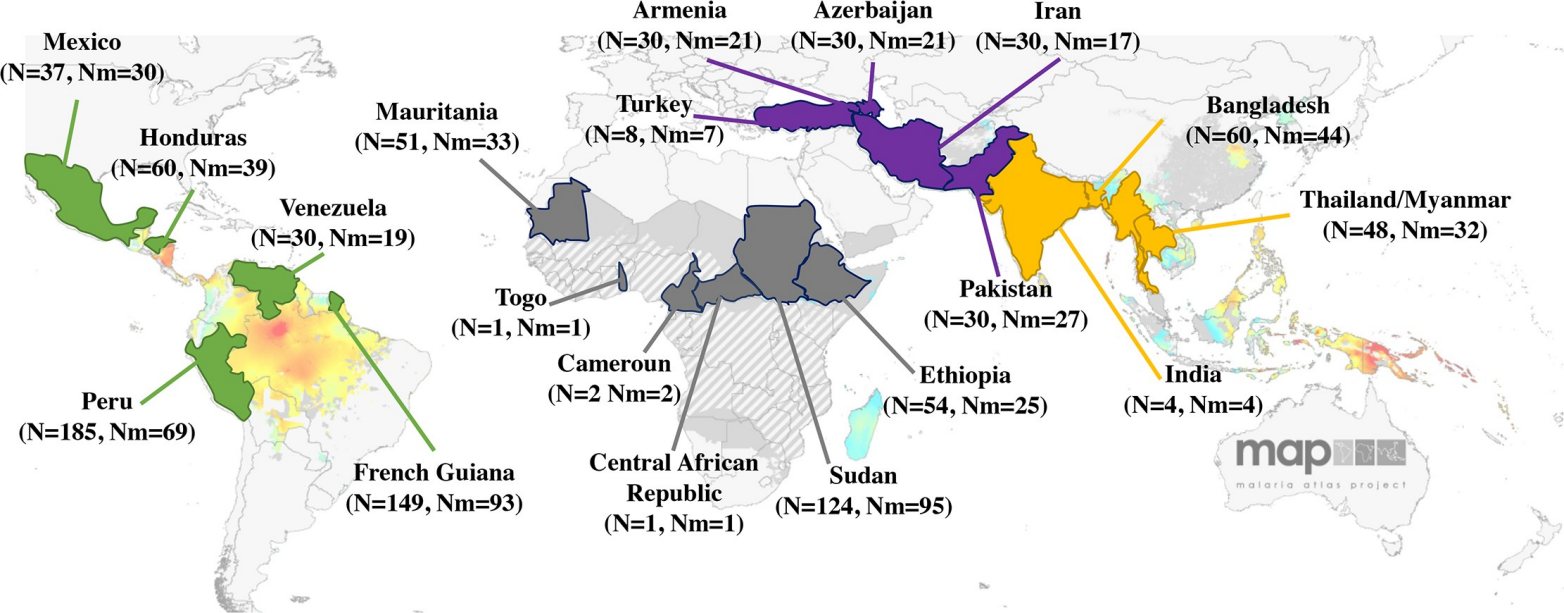
Worldwide samples distribution per country. For each country are indicated the number of samples collected (N) and the number of mono-infected samples (Nm). In yellow are represented Asian countries, in purple the Middle-east countries, in grey African countries and in green American countries. Map adapted from Malaria Atlas Project (MAP), University of Oxford.

We studied 834 *P*. *vivax*-infected human blood samples from 28 localities in 20 countries: 233 from Africa (Cameroon, Central African Republic, Ethiopia, Mauritania, Sudan and Togo), 361 from Central and South America (Mexico, Honduras, Venezuela, Peru and French Guiana), 128 from the Middle East (Armenia, Azerbaijan, Iran, Pakistan and Turkey) and 112 from Asia (Bangladesh, Laos, India and Thailand) (Fig 1 and S1 Table). The two populations sampled in Pakistan being actually close to the Iranian border and the Gulf of Oman, they were attributed to the Middle East instead of the more usual inclusion of Pakistan to Asia. Infected blood samples were collected from symptomatic patients either by venous puncture or by finger-pricks. All blood samples were collected after informed consent and conserved either as dried blood spot or whole blood. *P*. *vivax* infections were diagnosed using microscopy, PCR and / or RDT. Ethical clearance was obtained from local committees in each country sampled.

## Ethic statements

Ethical clearance was obtained from the local ethics committees in each country sampled. The informed consent procedure for the study consisted of a presentation of the aims of the study to the community followed by invitation of adult individuals for enrolment. At the time of sample collection, the purpose and design of the study was explained to each individual and a study information sheet was provided before oral informed consent was collected. Oral consent was provided since the study did not present any harm to subjects and did not involve procedures for which written consent is required. The verbal consent process was consistent with the ethical expectations for each country at the time of enrolment, approved by each country IRD and the ethics committees approved these procedures. Privacy and confidentiality of the samples data collected were ensured by the anonymization of all samples before the beginning of the study.

Samples from Bangladesh were collected under the protocol # 2008–049 and approved by the IRB: icddr,b Institutional Review Board (Federal Wide Assurance # FWA00001468, Institutional Review Board # IRB00001822 and IRB Organization Regn. # IORG0001386).

For samples from Mauritania, the study received the approval from the paediatric services of the National Hospital, the Cheikh Zayed Hospital and the Direction régionale à l’Action Sanitaire de Nouakchott (DRAS)/Ministry of Health in Mauritania. No ethic approval number obtained at this time. For samples from Comoros, Afghanistan, India, Senegal, Togo, Cameroon, CAR and Pakistan, no specific consent was required because of, in coordination with Santé Publique France organisation for the care and surveillance of malaria, the human clinical, epidemiological and biological data were collected in the CNRP database and analysed in accordance with the common public health mission of all French National Reference Centers (https://www.legifrance.gouv.fr/affichTexte.do?cidTexte=JORFTEXT000000810056&dateTexte=&categorieLien=id). The study of the biological samples obtained in the medical care context was considered as non-interventional research (article L1221-1.1 of the French public health code) only requiring the non-opposition of the patient during sampling (article L1211-2 of the French public health code). All data collected were anonymized before analyse.

For samples from Ethiopia, this study was approved by the Ethical Clearance Committee of Haramaya University-College of Health and Medical Sciences, and from the Harari and Oromia Regional State Health Bureau in Ethiopia.

For samples from Thailand, the blood collection protocol was approved by the ethical committee of the Institute of Health Research, Chulalongkorn University and the Ministry of Public Health, Thailand (Reference no. 101/2550 and 34/2557). The samples from the Thai-Myanmar border were collected as part of a study approved by both the Mahidol University Faculty of Tropical Medicine Ethics Committee (MUTM 2011–043, TMEC 11–008) and the Oxford Tropical Research Ethics Committee (OXTREC 17–11)

For samples from Armenia, Azerbaijan, Iran, Turkey, *P*. *vivax* was isolated from patients as part of routine primary diagnosis and post-treatment follow-up, with no unnecessary invasive procedures. The informed consent of each patient or an adult guardian of children enrolled in this study was obtained at the moment of blood collection and included information that samples would be used to investigate the genetic diversity of *Plasmodium* parasites (VIVANIS project, supported by the COPERNICUS-2 RTD project contract ICA2-CT-2000-10046 of European commission).

The samples from Mexico were obtained by CONACyT-Mexico project CB-2009-01-131247, Pan-American Health Organization (project MEX/07/005015) and the National Institute for Public Health (project CI624). This studies/projects were approved by the Ethics in Research Review Committee of the National Institute of Public Health (Mexico). Informed consent was obtained from all patients or the guardians of minors.

For samples from Honduras, scientific approval and ethical clearance was obtained from the Ethics Review Committee of the Infectious and Zoonotic Diseases Masters Program at UNAH (CEI-MEIZ 02–2014; 5/19/2014). R. Data were analysed anonymously since the study made secondary use of biological specimens originally collected for malaria diagnosis as per standard of care in Honduras.

Concerning the Peruvian samples, the study protocol was approved by both the Ethical Review Committee of the Universidad Peruana Cayetano Heredia and the Institute of Tropical Medicine, Antwerp, Belgium. The research was performed in accordance with the ethical standards of the Peruvian Ministry of Health. The trial has been registered as an International Standard Randomised Controlled Trial, number NCT00373607 at http://www.clinicaltrials.gov.

For samples collected in Venezuela, each patient gave written informed consent and ethical clearance was obtained from the Comité Ético Científico del Instituto de Medicina Tropical de la Universidad Central de Venezuela.

All the samples collected in French Guiana and analyzed in this study were from blood collections that were required as standard medical care for any patient presenting with a fever on admission to the hospital. According to French legislation (Article L.1211‐2 and related, French Public Health Code), biobanking and the secondary use of remaining human clinical samples for scientific purposes is possible if the corresponding patient is informed and has not objected to such use. This requirement was fulfilled for the present study; each patient was informed via the hospital brochure entitled “Information for Patients,” and no immediate or delayed patient opposition was reported to the Malaria NRC by the clinicians.

### Microsatellite genotyping

DNA from blood samples was extracted using DNeasy Blood and Tissue kit (Qiagen, France) according to manufacturer’s recommendations, and eluted in 100 μl of elution buffer per 200 μl of whole blood or per filter plot. For each DNA sample, whole genome amplification was carried out using an Illustra^™^ GenomiPhi^™^ V2 DNA Amplification Kit (GE Healthcare, Uppsala, Sweden) following the manufacturer’s instructions. The preparation (DNA + Sample buffer) was denatured by heating at 95°C for 3 min, and then kept on ice. 9 μL of Reaction Buffer (Illustra^™^ GenomiPhi^™^ V2 DNA Amplification Kit”—GE Healthcare, Uppsala, Sweden) and 1μL of enzyme Phi 29 was added, and the preparation was incubated at 30°C for 2 h for genome amplification. The amplification was stopped by placing the samples at 65°C for 10 min, before being stored at -20°C. The whole genome amplified DNA was used as template for the PCR-based amplification of 14 polymorphic microsatellite markers distributed among 10 of the 14 chromosomes of *P*. *vivax*. The PCR protocol was adapted from Karunawera et al. [17] as follow: a 20 μl reaction mix was made of 0.3 μl of each primer (10 μM), with the forward being labelled with fluorochrome, 0.1 μl template DNA, 0.5 μl dNTP mix (10 pmol/μl), 0.7 μl of MgCl_2_ (50mM), 2.5 of 10X buffer, 0.2 μl Taq polymerase (5 UI/μl, Invitrogen). Amplifications were carried out in a thermal cycler: 40 cycles of denaturation at 94°C for 30s, annealing temperature of each locus for 1 min at 58°C and extension at 72°C for 30s, followed by a final extension at 72°C for 7 min. The 14 polymorphic microsatellites are located on 10 of *P*. *vivax*’s 14 chromosomes (S2 Table). The reaction products were visualized on a 1.5% agarose gel stained with EZ-vision. Fluorescence-labelled PCR products were sized on Applied Biosystems ABI3500XL (GenSeq platform, Montpellier), with a Genescan 500 LIZ internal size standard. All 834 isolates were genotyped at all 14 loci.

### Multiplicity of infections

Blood samples are frequently infected with two or more haploid clones of *P*. *vivax*, resulting in the detection of two or more alleles at polymorphic loci. Isolates with more than one allele at any of the 14 microsatellite loci were removed from the analysis (Fig 1 and S1 Table), leaving 575 samples available for population genetic analyses.

### Microsatellite markers under natural selection

Natural selection may impact genetic frequencies differently than demography. We searched for microsatellite loci that could be under positive or balancing selection, using the method developed by Beaumont et al. [22] and implemented in the program LOSITAN [23]. Outliers values of genetic differentiation at specific loci were detected by running coalescent simulations to generate the expected distribution of Wright’s inbreeding coefficient *F*_ST_ as a function of expected heterozygosity. The distribution of *F*_ST_ was obtained by simulating an island model with a distribution centred on the empirical estimated average over loci. This average *F*_ST_ cannot be assumed to be neutral because (initially unknown) selected loci may be included in the computation. We ran LOSITAN to determine a subset of candidate selected loci and then removed them for the computation of the neutral *F*_ST_. A total of 100,000 coalescent simulations were carried out with 95% confidence intervals and a false discovery rate of 0.1. The value obtained is likely to be a better approximation of the neutral *F*_ST_. The approach is expected to be robust with respect to variation in mutation rate among loci, sample size, and departure from mutation/drift equilibrium [22].

### Population genetic analyses

Genetic diversity was estimated within each population and continent using Nei’s unbiased estimate of expected heterozygosity *Hs* [24]. We also computed the overall allelic richness for each population using the method implemented in the Hierfstat R package [25], which uses a rarefaction method to estimate allelic richness while considering for sample size disparities. Multilocus linkage disequilibrium was estimated for each population using the Agapow and Burt’s rdbar. Weir and Cockerham’s estimates of genetic differentiation were computed in a two-level model with a population effect nested inside a continent effect, using the Hierfstat R package [25]. To test the significance of the between-continents *F*_ST_ we employed a permutation test. Namely, for each pair of continents, the populations were randomly attributed to the continents and a new *F*_ST_ computed, this process being repeated 500 times. The p-value was taken as the proportion of simulated *F*_ST_ higher than the observed *F*_ST_. We then investigated patterns of isolation by distance (IBD) within each continent, by plotting genetic differentiation *F*_ST_ estimates against the geographic distance in kilometres between pairs of populations. Note that for IBD analysis the AFR population, composed of 4 individuals from Central Africa, Cameroon and Togo, was not included in the analysis due to the limited sample size and the absence of exact geographic locations. Pairwise geographic distances were computed using MAPINFO (Pitney Bowes). The significance of the relationship was assessed with a Mantel test using 10,000 permutations [26].

Genetic relationships between populations were visualized and assessed using (i) population trees, (ii) MultiDimensional Scaling (MDS) and (iii) Bayesian clustering methods (STRUCTURE analysis). Neighbour-joining trees with bootstrap resampling (N = 500) and MDS were constructed to examine the relationships between *P*. *vivax* populations all-over the world, based on Cavalli-Sforza distances computed between each pair of populations, a distance frequently used to reconstruct trees from population datasets and infer genetic relationship between populations [27]. At the population level, MDS was used to visualize the Cavalli-Sforza distance matrix. All these analyses were performed using the R software and specifically the APE package [28] and the ADE4 package [29]. Individuals were finally grouped based on their multi-locus genotypes using the Bayesian clustering method implemented in Structure 2.3.4 version [30]. Several models with possible admixture were run, with the number of clusters K ranging from 1 to the number of populations. All simulations used 100,000 Markov-chain Monte Carlo (MCMC) generations in the burn-in phase and 500,000 generations in the dataset collection step. To verify the convergence of the estimates of posterior probabilities, 10 independent runs were performed for each K value.

## Results

We discarded samples presenting evidence of multiple infection and ended up with a total of 575 mono-infected isolates from all 28 geographic localities in 20 countries (Fig 1, S1 and S3 Tables).

### *F*_ST_-based test of selection

Analyses of microsatellite markers often assume that they evolve neutrally, so that the distribution of allele frequencies is shaped by mutation, migration and genetic drift. However, because microsatellite loci may themselves be under selection or may be linked to other loci under selection, this assumption should be tested. The LOSITAN program employs coalescent simulations to estimate the distributions of heterozygosity and *F*_ST_ under the island model. Loci that do not fit neutral expectations are considered candidates of selection. The analysis of the overall genetic differentiation between populations suggested positive selection at two loci: MS3 and MS6 (S1 Fig). We searched for the position of these two microsatellite loci in *P*. *vivax* genome PvP01 [31] and identified them in two conserved *P*. *vivax* proteins of unknown function (PVP01_0410500 and PVP01_1306500). For these reasons, in the next steps of the analysis, we only present the results obtained for a dataset that excludes these two loci (MS3 and MS6) in order to avoid biased and spurious conclusions (S2 Fig).

### Genetic diversity and linkage disequilibrium

**Fig 2:**
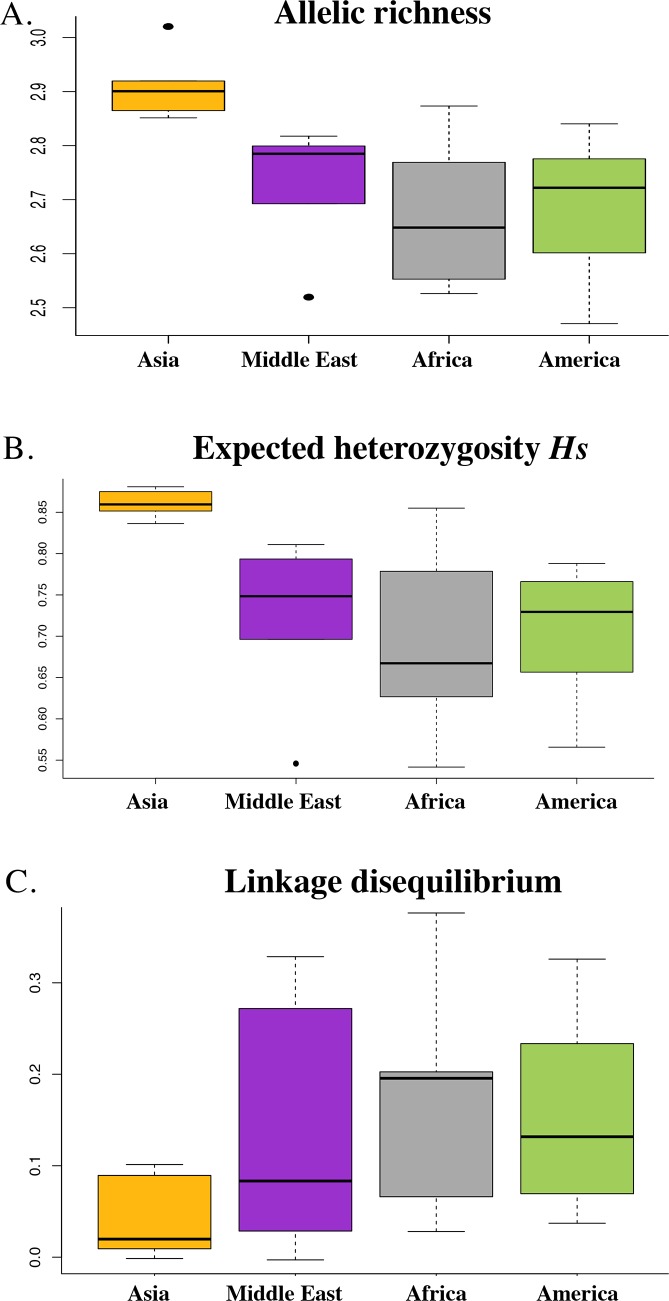
Overall allelic richness, *Hs* and rDbar were estimated using all loci for each population. A. Allelic richness for each continent. B. Hs for each continent. C. rDbar for each continent. Allelic richness, *Hs* and rDbar were estimated overall loci for each population. Boxes represent the interquartile range between first and third quartiles and the line inside represents the median of the estimate per population. Points represent outliers beyond the whiskers. The different regions of the world are represented using different colors: South East Asia (in yellow), the Middle East (in purple), Africa (in grey) and America (in green).

South East Asian populations present the highest allelic richness (Fig 2A) and genetic diversity (*Hs*Asia = 0.865; *Hs*MiddleEast = 0.788; *Hs*Africa = 0.820; *Hs*America = 0.820) (Fig 2B) in comparison to the Middle East, African and South American populations. Consistent patterns of allelic richness and expected heterozygosity are observed when considering each locus separately (S3 and S4 Figs, S4 and S5 Tables). Regarding linkage disequilibrium (as estimated by the rDbar), the South East Asian populations present the lowest levels followed by the Middle East, Africa and South American populations (Fig 2C).

### Population genetic structure

**Fig 3:**
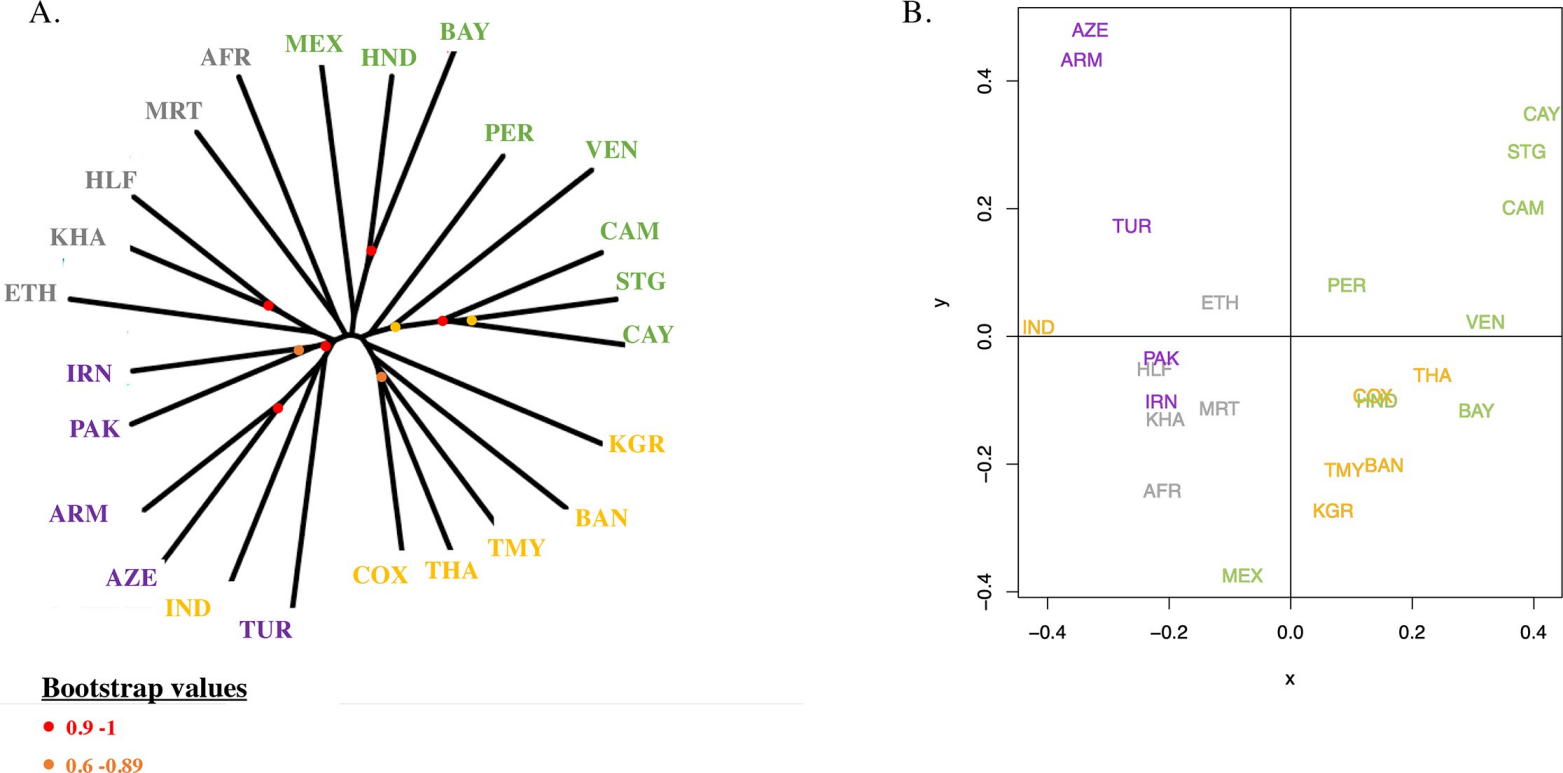
Worldwide genetic structure of 575 *P*. *vivax* isolates collected around the world. A. Neighbor-joining tree of isolates based on Cavalli-Sforza distance, with bootstrap resampling (N = 500); Red and orange dots indicate the bootstrap values, ranging from 0.9 to 1, and from 0.6 to 0.89 respectively. B. MultiDimensional Scaling representation. AFR: Central African Republic + Cameroon + Togo; ARM: Armenia; AZE: Azerbaijan; BAN: Bandarban; BAY: Bay Islands; CAM: Camopi; CAY: Cayenne; COX: Cox’s Bazar; ETH: Ethiopia; HLF: New Halfa; HND: Honduras; IND: India; IRN: Iran; KGR: Khagrachari; KHA: Khartoum; MEX: Mexico; MRT: Mauritania; PAK: Pakistan; PER: Peru; STG: Saint Georges de l’Oyapock; THA: Thailand; TMY: Thailand/Myanmar; TUR: Turkey; VEN: Venezuela. In yellow are represented Asian countries, in purple Middle-east countries, in grey African countries and in green American countries.

**Fig 4:**
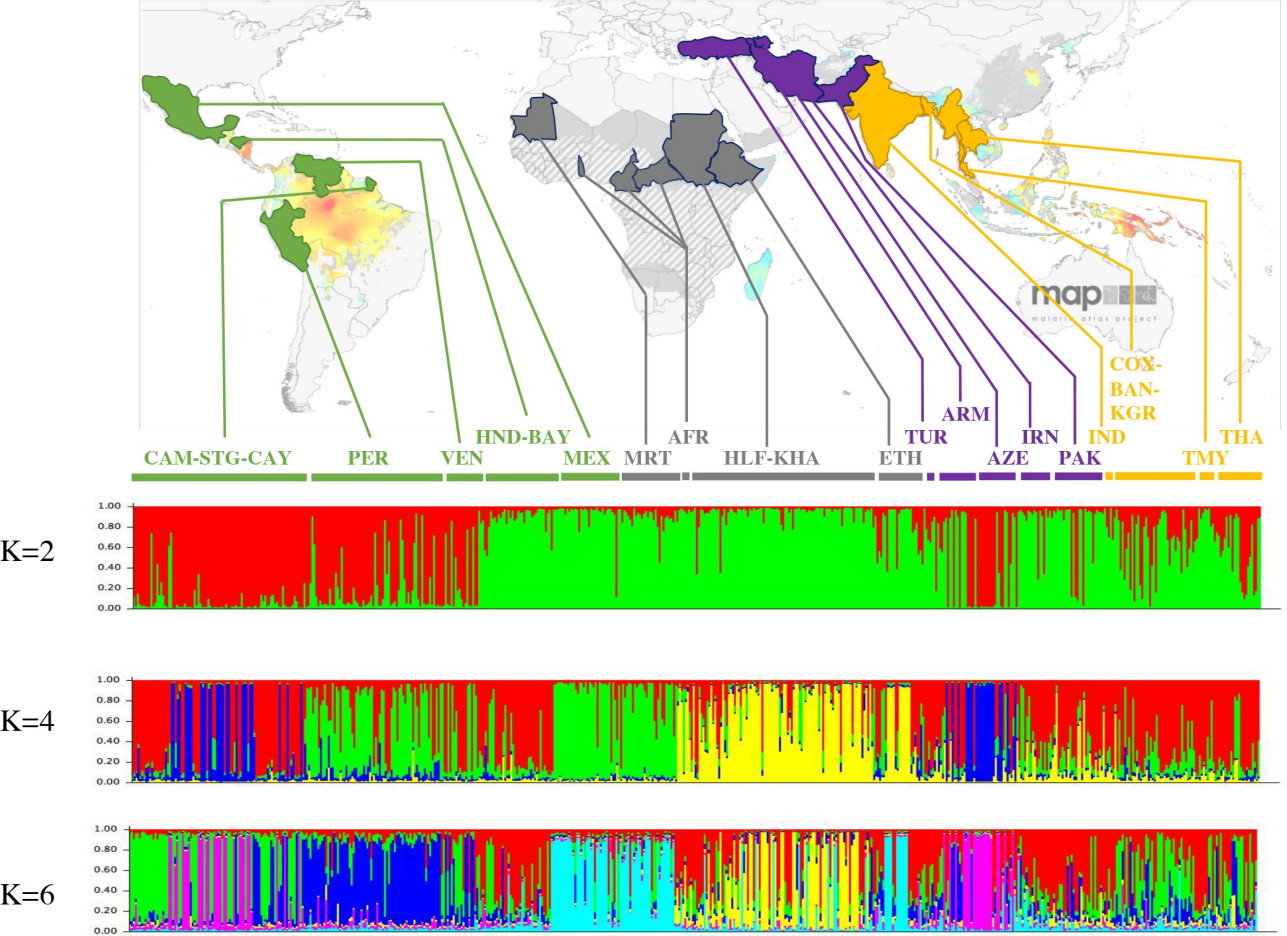
Bayesian cluster analysis on 575 *P*. *vivax* isolates collected around the world, using STRUCTURE software, for K = 2, K = 4 and K = 6. Map adapted from Malaria Atlas Project (MAP), University of Oxford. AFR: Central African Republic + Cameroon + Togo; ARM: Armenia; AZE: Azerbaijan; BAN: Bandarban; BAY: Bay Islands; CAM: Camopi; CAY: Cayenne; COX: Cox’s Bazar; ETH: Ethiopia; HLF: New Halfa; HND: Honduras; IND: India; IRN: Iran; KGR: Khagrachari; KHA: Khartoum; MEX: Mexico; MRT: Mauritania; PAK: Pakistan; PER: Peru; STG: Saint Gorges de l’Oyapock; THA: Thailand; TMY: Thailand/Myanmar; TUR: Turkey; VEN: Venezuela. In yellow are represented Asian countries, in purple Middle-east countries, in grey African countries and in green American countries.

Both the neighbour-joining tree and the MDS show that *P. vivax* populations cluster together according to geographic origin (Fig 3A and 3B), although we note that many of the clades in the NJ tree have low bootstrap values. For the Asian populations, all populations clustered together, except the Indian one that clustered with the middle east populations (Fig 3A and 3B). All American isolates are regrouped into two clusters, one corresponding to South American populations (French Guiana, Peru and Venezuela) and one to Central American populations (Honduras populations and Mexico). Based on the Bayesian clustering analysis, the Asian, the Middle East and East African (Ethiopia and Sudan) populations appear to be less structured by country and more diversified, in comparison to American populations (Fig 4). Within the American continent, the Mexican *P*. *vivax* population appears to be associated with the Mauritanian population. Indeed, when considering the Bayesian clustering analysis (under the models with K varying from 4 to 8), Mexico is assigned to the same group as Mauritania (Fig 4). Mauritania isolates are also separated from all other African isolates (Fig 4), whereas African isolates from Ethiopia and Sudan appear to be genetically closer to the Middle East and Asian isolates.

Genetic differentiations between continents varied from moderate between African, Asian and American populations (*F*_ST_ values varying between 0.139 and 0.164) to particularly high between the Middle East *P*. *vivax* populations and the other continents (all *F*_ST_ values > 0.294; significant *F*_ST_ with America) (S6 and S7 Tables). In Asia/Middle East and South America, there are patterns of isolation by distance, with genetic differentiation significantly (*F*_ST_) increasing with geographic distance between populations (S5 Fig). This pattern was not observed for the African populations.

## Discussion

To evaluate the structure, genetic diversity and evolutionary origins of *P*. *vivax*, we genotyped 834 *P*. *vivax* isolates sampled from across the global range of this parasite species, including several populations from the African continent. After excluding samples from individuals with multiple infections, genetic analyses were performed on a total of 575 mono-infected isolates from all 28 geographic localities in 20 countries (Fig 1, S1 and S3 Tables).

### Worldwide genetic structure of *P*. *vivax*

Results based on NJ tree, MDS and structure analyses reveals patterns of population genetic structure that are consistent with previous studies (e.g. [16–21]). The South East Asian populations are the most diverse populations as measured by allelic richness and genetic diversity *Hs* and present the lowest levels of multilocus linkage disequilibrium (Fig 2). In contrast, populations from other regions of the world (Middle East, Africa and America) display lower levels of genetic diversity as well as higher levels of linkage disequilibrium (Fig 2). *P*. *vivax* populations are genetically differentiated between continents, with significantly higher *F*_ST_ values for Middle Eastern populations than for others (S6 and S7 Tables). Increased differentiation between populations in the Middle East could be explained by the fact that many of the countries in this region, including Azerbaijan, are close to elimination of *P*. *vivax*. Such a situation could lead to a reduced effective population size and limited gene flow, resulting in greater genetic differentiation between local parasite populations within this region in comparison with worldwide *P*. *vivax p*opulations. Within each continent, populations are genetically differentiated and patterns of isolation by distance are observed (S5 Fig), indicating that gene flow mainly occurs between close populations. A high genetic differentiation is observed between East and Western African *P*. *vivax* populations (S7 Table), which can be explained by the geographic and ecological separation of these populations limiting migration and gene flow. This could also be due to the fact that these populations were founded separately from different source populations or from the same source at different times. In the Americans, the high genetic differentiation values obtained between *P*. *vivax* populations from Mexico and the others could be explained by multiple factors, such as a low effective population size, some reduced gene flow with the other American populations and/or a different origin compared the other American populations (as discussed in more detail below).

At a worldwide scale, populations tend to group together according to their geographic locations but the relationships between populations of different geographic origins are far less obvious (Figs 3 and 4). Our data indeed provide little information on the inter-continental relationships between populations, certainly because the number of markers that were used in our study is low (12 microsatellite markers) thus limiting our ability to reconstruct the genealogical relationships between populations of different continents. Several observations can nevertheless be noted (although they need to be taken with caution and validated by larger datasets): (i) the east African populations seem to be more related to the middle east populations than they are to the western populations of Africa (e.g. Mauritania); (ii) some populations of America look related to the Asian populations while others (those from central America) look more related to certain African populations.

The variations of genetic diversity and genetic differentiation observed among populations at a worldwide scale are likely the consequence of the history of colonisation of the world by *P*. *vivax* as well as the effect of more recent demographic events like population contraction, expansion or founding events.

### *P*. *vivax* origin and worldwide spread: “Out of Asia” *versus* “Out of Africa”?

Two scenarios have been proposed to explain the origin of *P*. *vivax* and its spread throughout the world: the “Out of Asia” scenario and the “Out of Africa” scenario. Although these two hypotheses were based on different lines of evidence, the “Out of Africa” hypothesis has enjoyed renewed interest in recent years following the discovery of parasites closely related to *P*. *vivax* in African apes (named *P*. *vivax-like*) and the observation that human *P*. *vivax* diversity appears to be nested within that of the *P*. *vivax-like* parasites. This discovery has led some researchers to suggest that human strains of *P*. *vivax* originated following the transfer of parasites from African great apes to humans [32,33]. The second element that has been advanced in support of this hypothesis is the absence of circulation of *P*. *vivax* in sub-Saharan human populations, associated to the absence at the surface of their red blood cells of the Duffy antigen (i.e. Duffy negativity), suggesting that the Duffy-negative mutation was selected a long time ago in response to *P*. *vivax* infection [34]. However, several remarks should be made concerning these interpretations. First, the apparent nested relationship between human and non-human strains of *P*. *vivax* was inferred from just a few nuclear genes and partial mitochondrial genomes and is consistent with several alternative scenarios. Indeed, such phylogenetic patterns can also be obtained because of incomplete lineage sorting or because of a lack of phylogenetic signal for the sequences used, two phenomena that are frequent when species have recently diverged (which is the case for *P*. *vivax* and *P*. *vivax*-*like*). We recently showed, comparing *P*. *vivax-like* genomes to those of *P*. *vivax*, that incongruent phylogenies may be obtained when one limits the analyses to a single or a couple of genes [35]. Second, how could we explain an “Out of Africa” origin of *P*. *vivax*, with the high genetic diversity obtained in current Asian *P*. *vivax* parasite populations in comparison to the rest of worldwide *P*. *vivax* parasites? This pattern of genetic diversity is the opposite for *P*. *falciparum*, which has a well-established African origin [36]. Indeed, for this parasite, the highest genetic diversity is found in Africa and decreases toward Asia, the opposite pattern obtained in *P*. *vivax* [36]. Finally, the phylogenetic position of *P*. *vivax* among *Plasmodium* parasite species infecting mostly wild Asian monkeys (about fifteen monkey species’) also calls into question the “Out of Africa” hypothesis.

If the history of colonisation has been important in shaping the distribution of genetic diversity of *P*. *vivax* at a worldwide scale, then our results based on microsatellite markers, would be more congruent with an Asian origin and a spread from Asia of the current populations of *P*. *vivax* found worldwide in humans. Genetic diversity and allelic richness are highest in South-East Asian *P*. *vivax* populations and decrease from South East Asia to East Africa (Fig 2A and 2B), with a significant isolation by distance pattern (S5 Fig), suggesting that the source population of human *P*. *vivax* was in Southeast Asia and then spread to Africa following a step-by-step colonisation process characterized by recurrent bottlenecks during which populations have lost genetic diversity (S5 Fig). Such a scenario could also explain the patterns of linkage disequilibrium observed in the populations (Fig 2C), with the populations which have been founded the most recently displaying the highest levels of linkage.

One may nevertheless advocate that some of these genetic patterns (e.g. the decrease of genetic diversity from Asia to Africa) could also be the consequence of the current differences in population size of the *P*. *vivax* populations in the different regions of the world, the largest population sizes and transmission levels being observed in Asia for *P*. *vivax*. This is indeed a possibility and our genetic data (e.g. a few microsatellite markers) are clearly insufficient to disentangle the impact of recent and ancient demographic events on the distribution of *P*. *vivax* genetic variability. More extensive analyses of the population genetic structure of these parasites, using whole genome data and some tests of demographic scenarios, might be necessary to resolve the origin and evolution of these parasites.

### *P*. *vivax* evolutionary history in the Americas

Within the Americas, a separation is observed between *P*. *vivax* populations from South America (French Guiana, Venezuela and Peru) and Central America (Mexico and Honduras) (Fig 4). When considering the Bayesian clustering analysis (Fig 4), it seems that South American *P*. *vivax* populations are more related to the Asian populations while the Central American *P*. *vivax* populations (in particular the Mexican one) appear to be more closely related to some African populations (the Mauritanian *P*. *vivax* population), thus suggesting a double origin of *P*. *vivax* in America (Asian and African). Moreover, we found that American *P*. *vivax* populations harbour low levels of genetic variation. These results are consistent with a scenario in which American *P*. *vivax* populations were introduced from multiple sources. This possibility of introduction from multiple locations has already been mentioned in several recent studies based on the analysis of different kinds of genetic markers [37,38]. Rodrigues et al. proposed that African and South East Asian *P*. *vivax* populations contributed to the current diversity of *P*. *vivax* observed in South America [38]. Carter et al. suggested that a large part of the genetic diversity observed today was due to gene flow from Western Pacific to the Americas, which needs to be considered when considering the Australian and Eurasian ancestral origin of three south American native human populations [34]. Gelabert et al. showed based on the mitogenome analysis from slides of European *P*. *vivax* dated between 1942 and 1944 that European isolates were closely related to the most common present-day American haplotype [39]. This suggested that *P*. *vivax* likely entered the American continent during the post Columbian contact [39]. To conclude, South American *P*. *vivax* parasites seem to be characterized by a recent history through multiple introductions and the next steps will be to identify the sources of introduction and to date such events.

This study confirmed the worldwide genetic structure of *P*. *vivax* populations. The highest genetic diversity is again observed in South East Asian *P*. *vivax* populations in comparison to the populations of the rest of the world, which argues again the suggested African origin of current human *P*. *vivax*. Concerning the colonization of the American continent, results obtained in our study seem to suggest a double origin from Africa (or Europe) and Asia, as already suggested by different studies [34,37–39]. In order to resolve the origin and evolution of these protozoan parasites, more extensive genomic analyses of the genetic diversity and structure of *P*. *vivax* are needed, including the study of ancient European *P*. *vivax* strains.

## Supporting information

S1 Fig *F*_ST_ values plotted against heterozygosity (*He*) and computed using the microsatellite loci dataset with LOSITAN program after 100,000 coalescent simulations. The microsatellite markers suspected to be under positive selection (MS3 and MS6) are displayed. Grey: area including 95% of the neutral *F*_ST_ computed using an island model. Red: area including the highest 5% neutral *F*_ST_. Yellow: area including the lowest 5% neutral *F*_ST_.
(PDF)

S2 Fig Neighbor-joining trees of isolates based on Cavalli-Sforza distance, with bootstrap resampling (N = 500), obtained based on 14 microsatellite markers and on 12 (without MS3 and M6, found under positive selection). Red and orange dots indicate the bootstrap values, ranging from 0.9 to 1, and from 0.6 to 0.89 respectively. The only difference detectable is that the AFR+MRT cluster is associated to the Central American populations when considering the full microsatellite markers set, whereas it is distinct when getting rid of the two markers detected under positive selection (MS3 and MS6). AFR: Central African Republic + Cameroon + Togo; ARM: Armenia; AZE: Azerbaijan; BAN: Bandarban; BAY: Bay Islands; CAM: Camopi; CAY: Cayenne; COX: Cox’s Bazar; ETH: Ethiopia; HLF: New Halfa; HND: Honduras; IND: India; IRN: Iran; KGR: Khagrachari; KHA: Khartoum; MEX: Mexico; MRT: Mauritania; PAK: Pakistan; PER: Peru; STG: Saint Gorges de l’Oyapock; THA: Thailand; TMY: Thailand/Myanmar; TUR: Turkey; VEN: Venezuela. In yellow are represented Asian countries, in purple Middle-east countries, in grey African countries and in green American countries.
(PDF)

S3 Fig Allelic richness for each locus analyzed in Asia (As), Middle East (Me), Africa (Af) and America (Am). In yellow are represented Asian countries, in purple Middle-east countries, in grey African countries and in green American countries.
(PDF)

S4 Fig Expected heterozygosity *Hs* for each locus analyzed in Asia (As), Middle East (Me), Africa (Af) and America (Am). In yellow are represented Asian countries, in purple Middle-east countries, in grey African countries and in green American countries.
(PDF)

S5 Fig Isolation by distance (IBD) with Asian/Middle East and American continents. Pairwise geographic along landmasses plotted against pairwise genetic differentiation (F_ST_). A. Asian/Middle East continent and B. American continent. Mantel tests gave *P-values* < 0.05 for both continents tested.
(PDF)

S1 Table Characteristics of the *P*. *vivax* samples with geographic locations, year of collection, short name attributed for the analysis, geographic coordinates, total sample size and mono-infected sample size. All isolates were genotyped by us for the 14 microsatellite loci. NA: non-available information.
(DOCX)

S2 Table The 14 *P*. *vivax* microsatellite loci with marker code, chromosome number in Salvador-1 strain (and the protein associated code), and size range of the amplified alleles in base pairs. Locations of markers are given according to the *P*. *vivax* reference genome available at the Institute for Genomic Research website (http://www.tigr.ord/tdb/e2k1/pva1). For markers on the same chromosome, distances are enough to limit any physical linkage: MS2, MS5 and MS6 are at a minimum at 0.15Mb apart from each others; MS7 and MS8 are 1.14Mb appart; MS12 and MS15 are 1.04Mb appart.
(DOCX)

S3 Table Microsatellite dataset obtained in this study, with each country, locality, sample ID, year of collection and all genotypes obtained at each locus by PCR. NA: Not available information. For microsatellite genotypes, blank cases correspond to the absence of readable amplification/genotype.
(DOCX)

S4 Table Number of alleles per locus and population. AFR: Central African Republic + Cameroon + Togo; ARM: Armenia; AZE: Azerbaijan; BAN: Bandarban; BAY: Bay Islands; CAM: Camopi; CAY: Cayenne; COX: Cox’s Bazar; ETH: Ethiopia; HLF: New Halfa; HND: Honduras; IND: India; IRN: Iran; KGR: Khagrachari; KHA: Khartoum; MEX: Mexico; MRT: Mauritania; PAK: Pakistan; PER: Peru; STG: Saint Gorges de l’Oyapock; THA: Thailand; TMY: Thailand/Myanmar; TUR: Turkey; VEN: Venezuela.
(DOCX)

S5 Table Expected heterozygosity (*Hs*) estimates computed within each population and for each locus. ARM: Armenia; AZE: Azerbaijan; BAN: Bandarban; BAY: Bay Islands; CAM: Camopi; CAY: Cayenne; COX: Cox’s Bazar; ETH: Ethiopia; HLF: New Halfa; HND: Honduras; IND: India; IRN: Iran; KGR: Khagrachari; KHA: Khartoum; MEX: Mexico; MRT: Mauritania; PAK: Pakistan; PER: Peru; STG: Saint Gorges de l’Oyapock; THA: Thailand; TMY: Thailand/Myanmar; TUR: Turkey; VEN: Venezuela.
(DOCX)

S6 Table *F*_ST_ values between continents, considering pairs of populations and *P-values* based on a permutation test are indicated in brackets. *P-value < 0.008 (Bonferroni correction).
(DOCX)

S7 Table *F*_ST_ values between pairs of populations. All significant values are indicated in bold (p-value < 0.05). AFR: Cantral African Republic + Cameroon + Togo; ARM: Armenia; AZE: Azerbaijan; BAN: Bandabar; BAY: Bay Islands; CAM: Camopi; CAY: Cayenne; COX: Cox’s Bazar; ETH: Ethiopia; HLF: New Halfa; HND: Honduras; IND: India; IRN: Iran; KGR: Khagrachari; KHA: Khartoum; MEX: Mexico; MRT: Mauritania; PAK: Pakistan; PER: Peru; STG: Saint Georges de l’Oyapock; THA: Thailand; TMY: Thailand/Myanmar; TUR: Turkey; VEN: Venezuela.
(DOCX)
