# Supplementary figures and images for: Human Plasmodium vivax diversity, population structure and evolutionary origin
Source: PLoS Negl Trop Dis. 2020 Mar 9;14(3):e0008072. doi: 10.1371/journal.pntd.0008072 (PMC7082039; doi:10.1371/journal.pntd.0008072)

# Fst/He

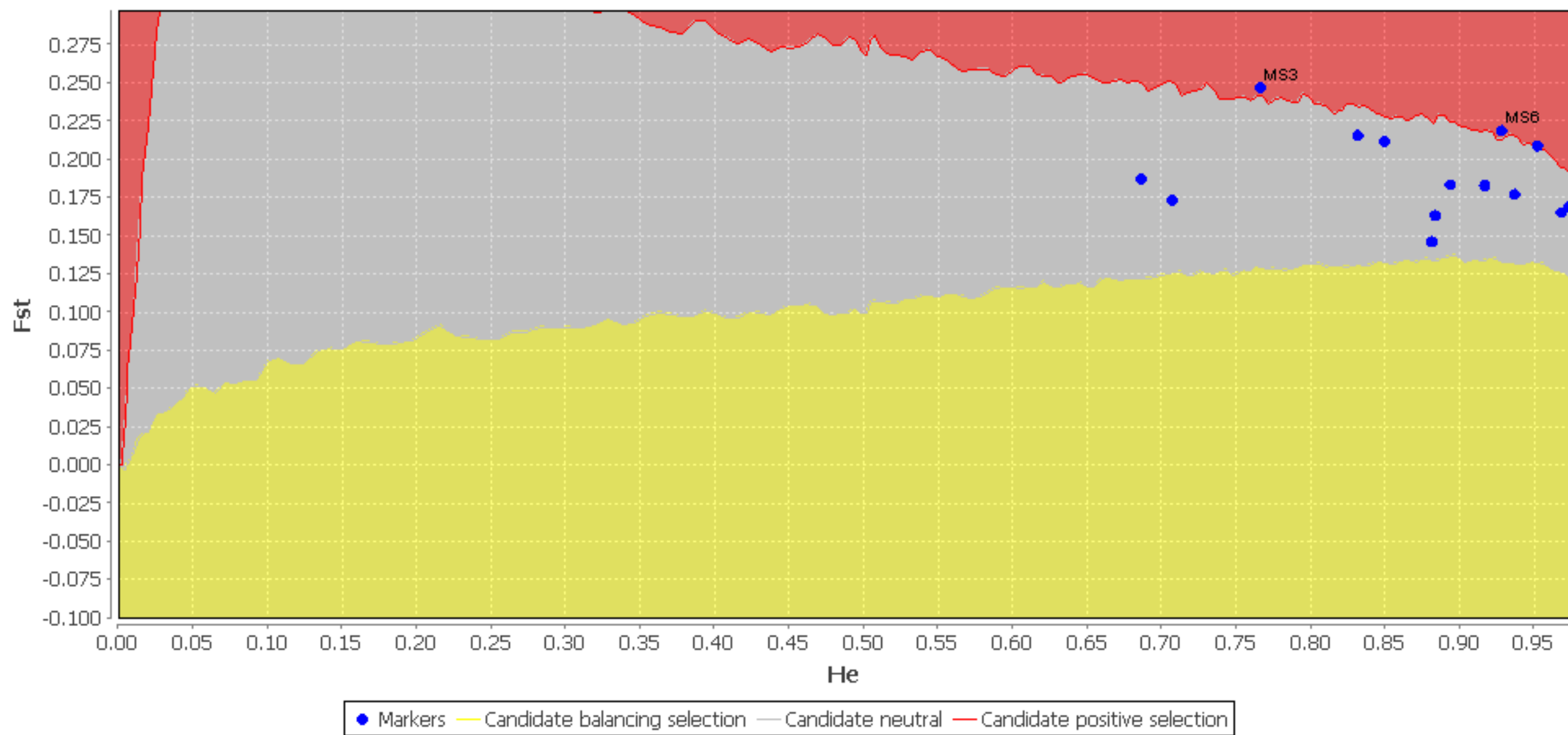

Supplement: S1 Fig — The microsatellite markers suspected to be under positive selection (MS3 and MS6) are displayed. Grey: area including 95% of the neutral FST computed using an island model. Red: area including the highest 5% neutral FST. Yellow: area including the lowest 5% neutral FST. (PDF) [file pntd.0008072.s001.pdf]

All microsatellite markers  
(n=14)

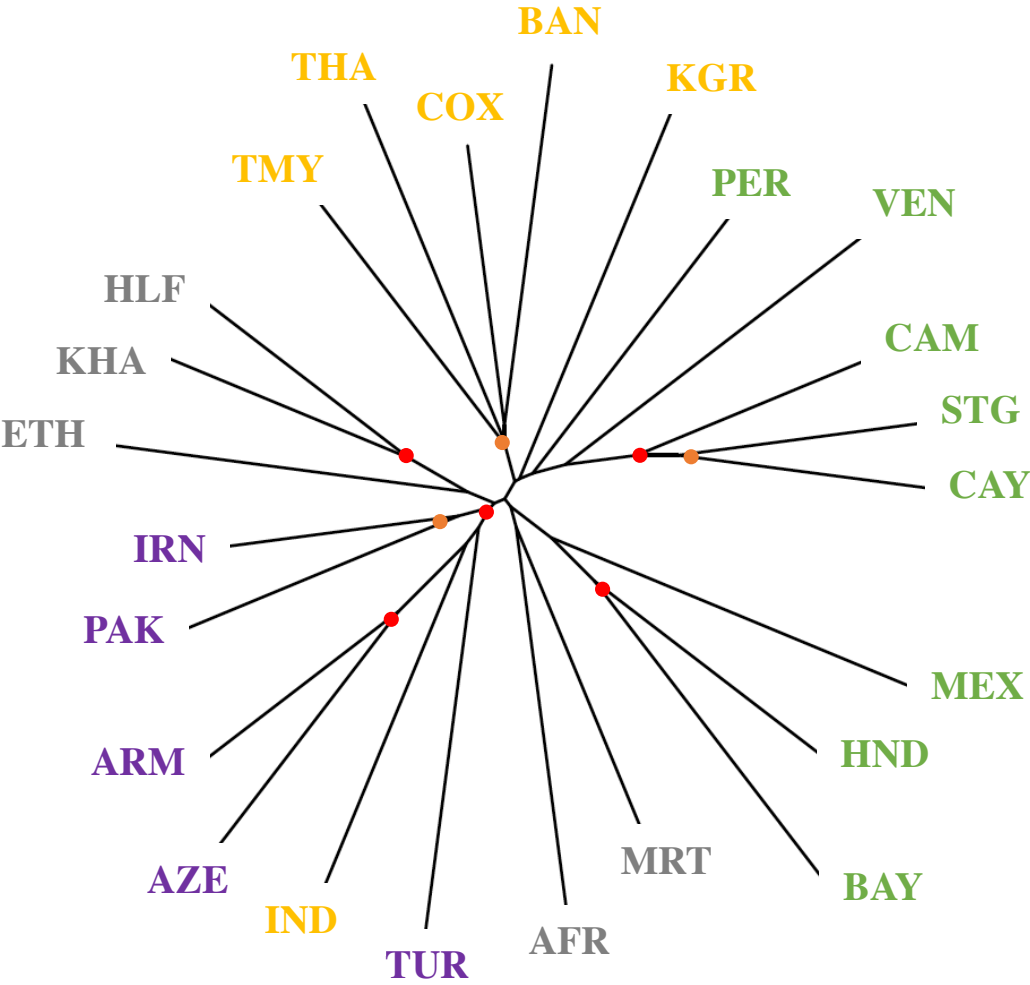

All microsatellite markers,  
except MS3 and MS6 (n=12)

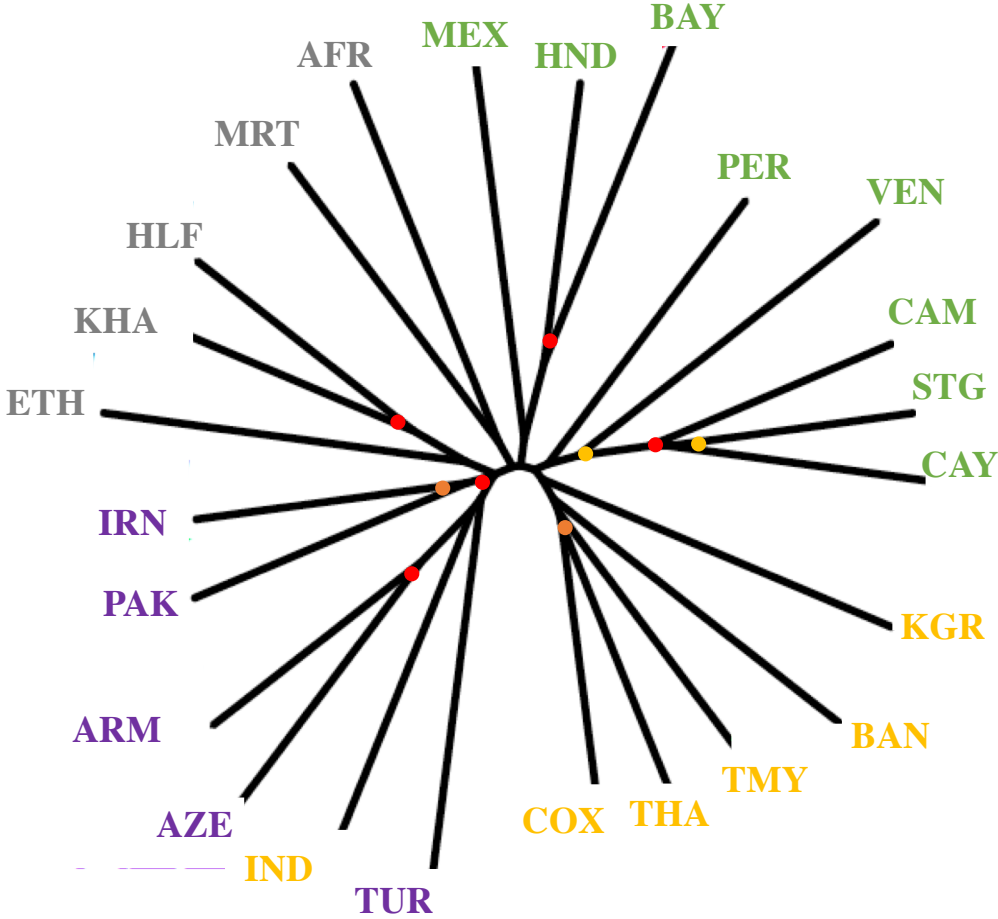

Bootstrap values

- 0.9 -1
- 0.6 -0.89

Supplement: S2 Fig — Red and orange dots indicate the bootstrap values, ranging from 0.9 to 1, and from 0.6 to 0.89 respectively. The only difference detectable is that the AFR+MRT cluster is associated to the Central American populations when considering the full microsatellite markers set, whereas it is distinct when getting rid of the two markers detected under positive selection (MS3 and MS6). AFR: Central African Republic + Cameroon + Togo; ARM: Armenia; AZE: Azerbaijan; BAN: Bandarban; BAY: Bay Islands; CAM: Camopi; CAY: Cayenne; COX: Cox’s Bazar; ETH: Ethiopia; HLF: New Halfa; HND: Honduras; IND: India; IRN: Iran; KGR: Khagrachari; KHA: Khartoum; MEX: Mexico; MRT: Mauritania; PAK: Pakistan; PER: Peru; STG: Saint Gorges de l’Oyapock; THA: Thailand; TMY: Thailand/Myanmar; TUR: Turkey; VEN: Venezuela. In yellow are represented Asian countries, in purple Middle-east countries, in grey African countries and in green American countries. (PDF) [file pntd.0008072.s002.pdf]

**Asia/Middle East**

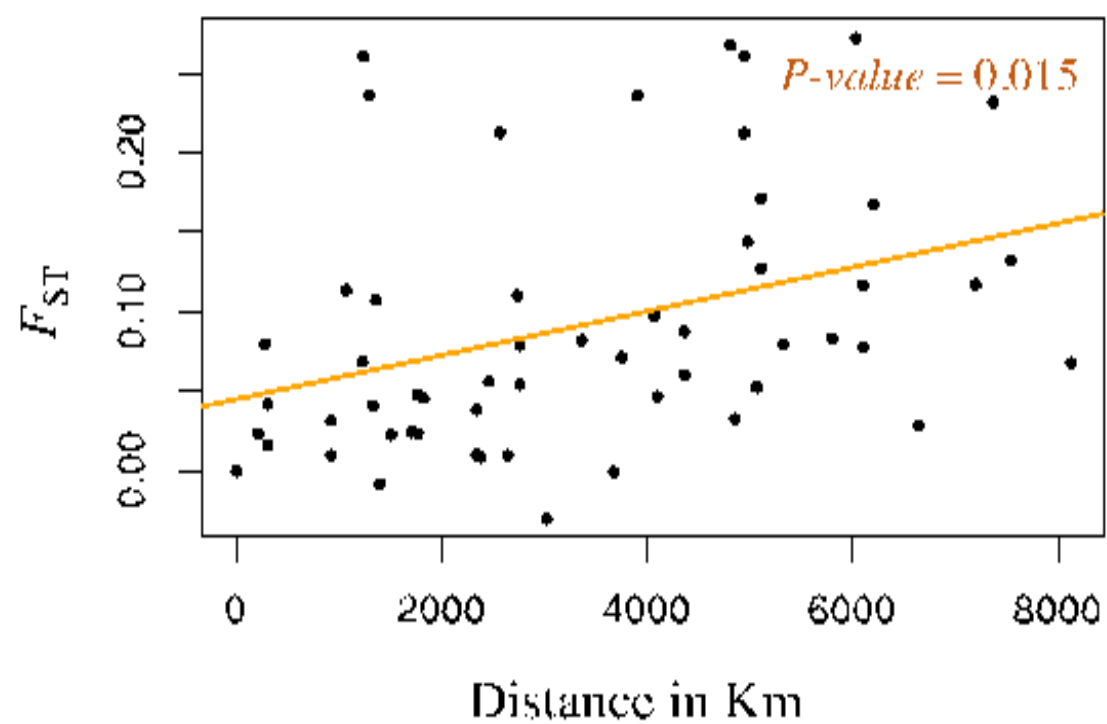

**America**

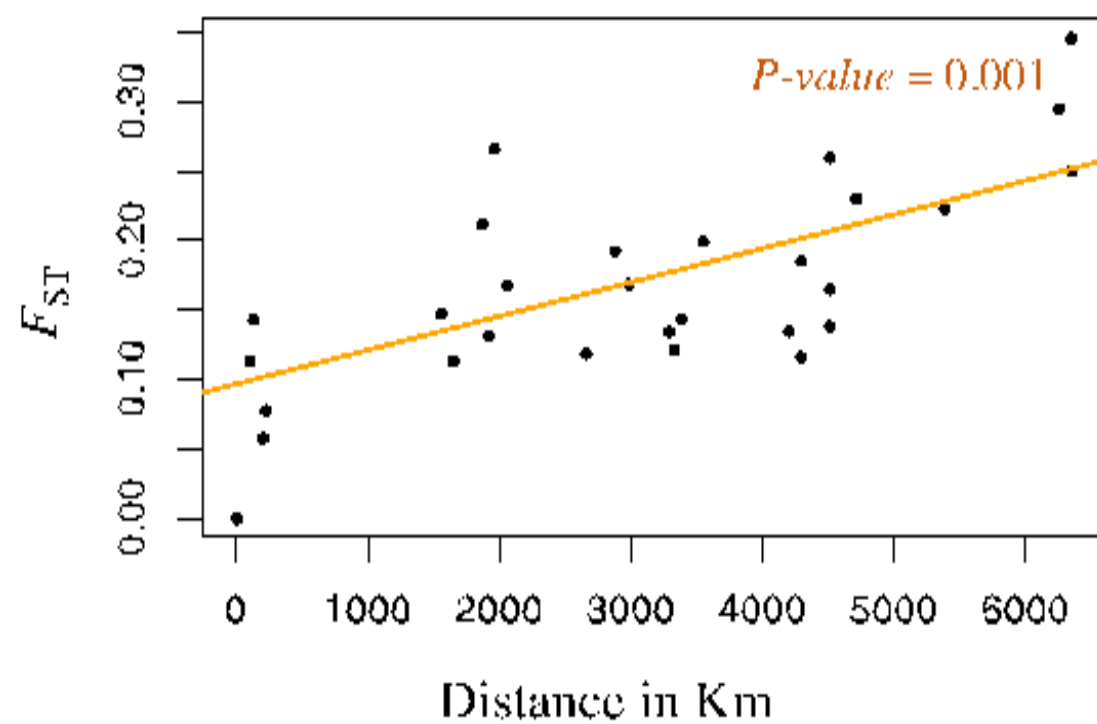

Supplement: S5 Fig — Pairwise geographic along landmasses plotted against pairwise genetic differentiation (FST). A. Asian/Middle East continent and B. American continent. Mantel tests gave P-values < 0.05 for both continents tested. (PDF) [file pntd.0008072.s005.pdf]
