# Supplementary material for: Human Plasmodium vivax diversity, population structure and evolutionary origin
Source: PLoS Negl Trop Dis. 2020 Mar 9;14(3):e0008072. doi: 10.1371/journal.pntd.0008072 (PMC7082039; doi:10.1371/journal.pntd.0008072)
Supplement: S3 Fig — In yellow are represented Asian countries, in purple Middle-east countries, in grey African countries and in green American countries. (PDF) [file pntd.0008072.s003.pdf]

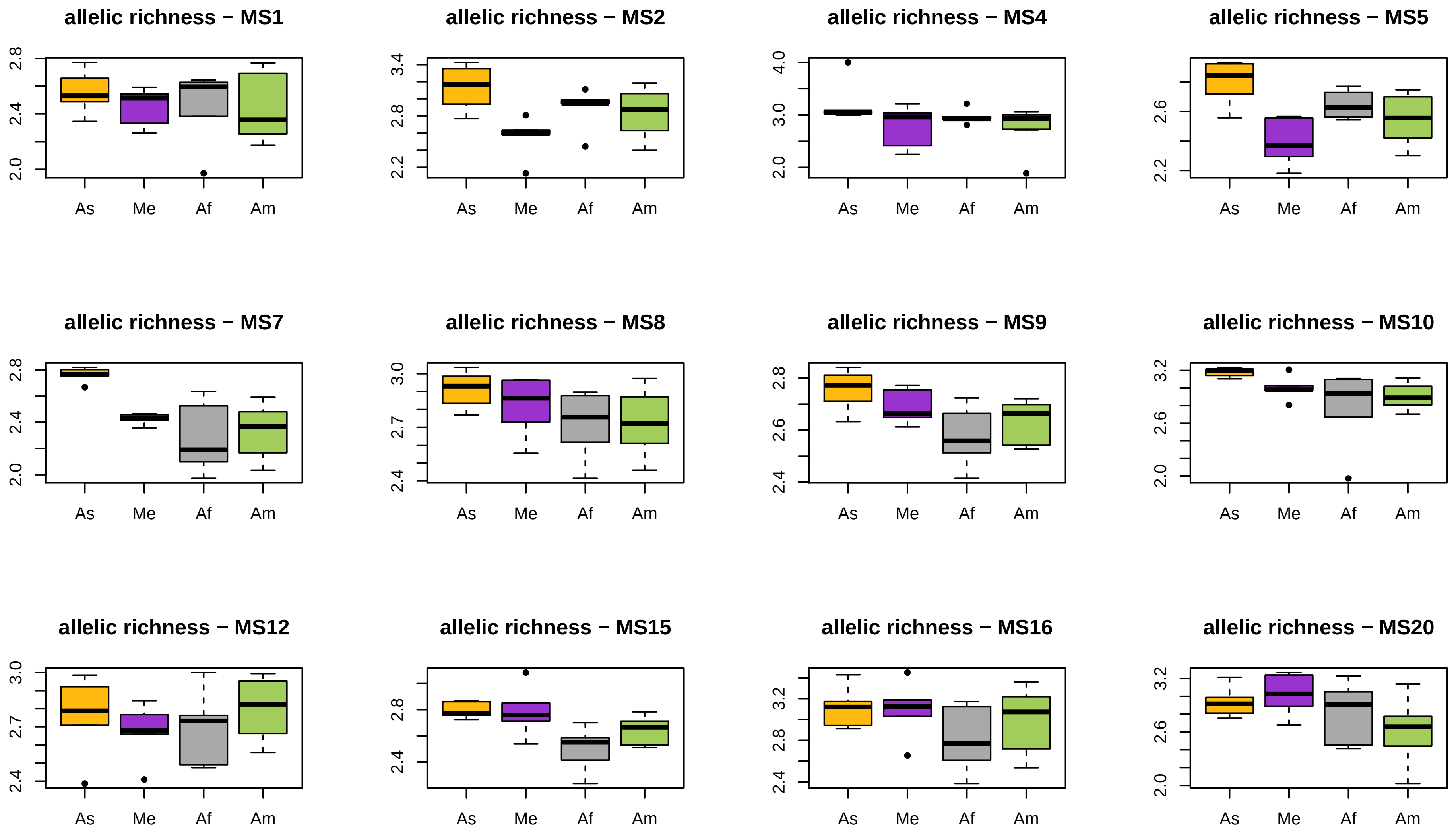
allelic richness − MS1
allelic richness − MS2
allelic richness − MS4
allelic richness − MS5
allelic richness − MS7
allelic richness − MS8
allelic richness − MS9
allelic richness − MS10
allelic richness − MS12
allelic richness − MS15
allelic richness − MS16
allelic richness − MS20
As
Me
Af
Am
