# Supplementary material for: Human Plasmodium vivax diversity, population structure and evolutionary origin
Source: PLoS Negl Trop Dis. 2020 Mar 9;14(3):e0008072. doi: 10.1371/journal.pntd.0008072 (PMC7082039; doi:10.1371/journal.pntd.0008072)
Supplement: S4 Fig — In yellow are represented Asian countries, in purple Middle-east countries, in grey African countries and in green American countries. (PDF) [file pntd.0008072.s004.pdf]

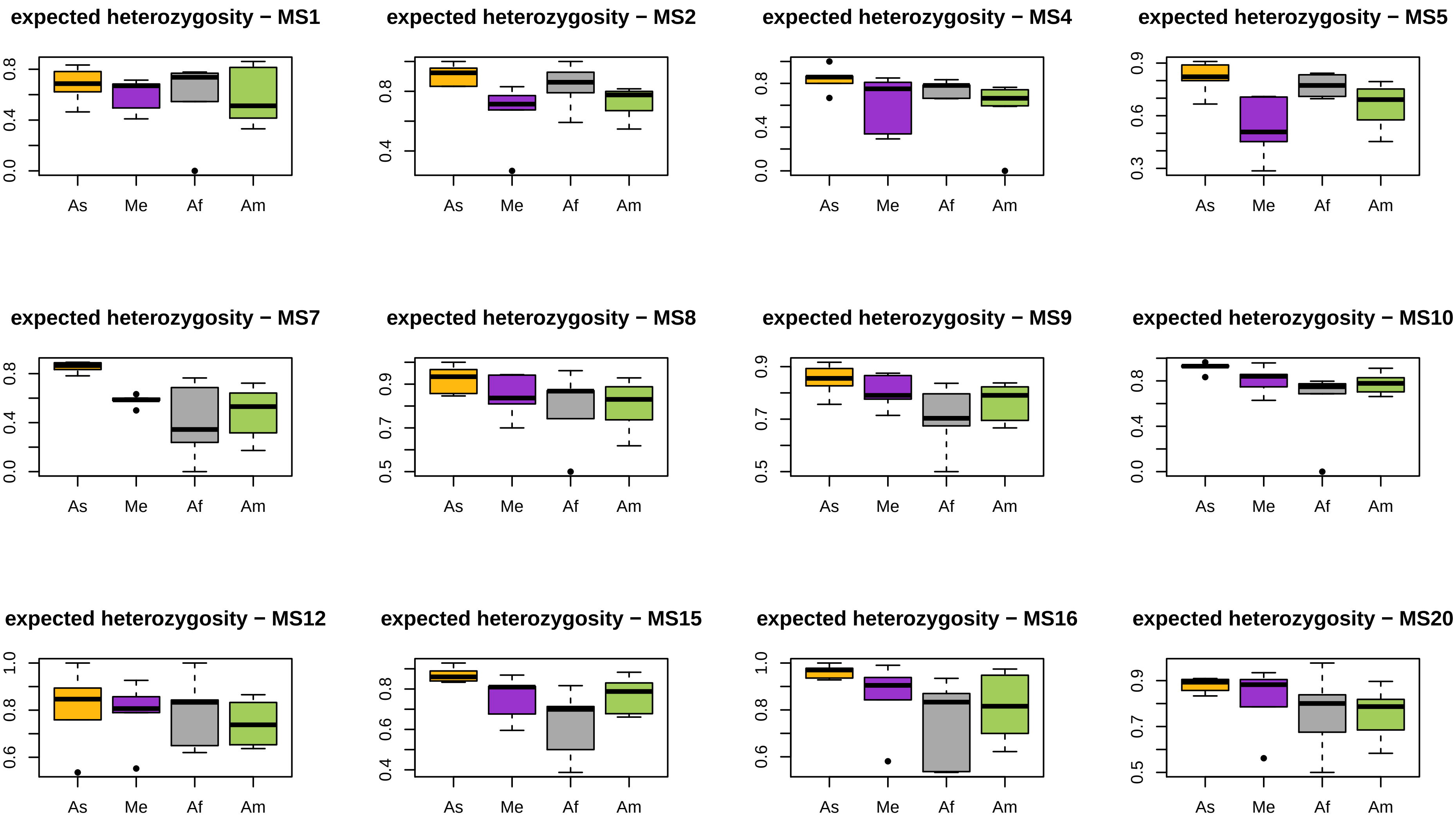
expected heterozygosity − MS1
0.0
0.4
0.8
As
Me
Af
Am
expected heterozygosity − MS2
0.4
0.8
As
Me
Af
Am
expected heterozygosity − MS4
0.0
0.4
0.8
As
Me
Af
Am
expected heterozygosity − MS5
0.3
0.6
0.9
As
Me
Af
Am
expected heterozygosity − MS7
0.0
0.4
0.8
As
Me
Af
Am
expected heterozygosity − MS8
0.5
0.7
0.9
As
Me
Af
Am
expected heterozygosity − MS9
0.5
0.7
0.9
As
Me
Af
Am
expected heterozygosity − MS10
0.0
0.4
0.8
As
Me
Af
Am
expected heterozygosity − MS12
0.6
0.8
1.0
As
Me
Af
Am
expected heterozygosity − MS15
0.4
0.6
0.8
As
Me
Af
Am
expected heterozygosity − MS16
0.6
0.8
1.0
As
Me
Af
Am
expected heterozygosity − MS20
0.5
0.7
0.9
As
Me
Af
Am
