# Supplementary material for: Human Plasmodium vivax diversity, population structure and evolutionary origin
Source: PLoS Negl Trop Dis. 2020 Mar 9;14(3):e0008072. doi: 10.1371/journal.pntd.0008072 (PMC7082039; doi:10.1371/journal.pntd.0008072)
Supplement: S1 Table — All isolates were genotyped by us for the 14 microsatellite loci. NA: non-available information. (DOCX) [file pntd.0008072.s006.docx]

**Table S1**.

| **Country** | **Site** | **Year of collection** | **Short name** | | **Latitude** | | **Longitude** | **Sample size** | **Mono-infected sample size** |
| --- | --- | --- | --- | --- | --- | --- | --- | --- | --- |
| **ASIA** | | | | | | | | | |
| **Thailand** | NA | 2005-2007 | THA | 100.99254 | | 15.87003 | | 18 | 10 |
| **Thailand/Myanmar** | Border Thailand/Myanmar | 2015 | TMY | 101.975766 | | 4.210484 | | 30 | 22 |
| **India** | NA | 2015 | IND | 78.96288 | | 20.59368 | | 4 | 4 |
| **Bangladesh** | Bandarban | 2014 | BAN | 92.36863 | | 21.8311 | | 16 | 13 |
|  | Khagrachari | 2014 | KGR | 91.94902 | | 23.13218 | | 13 | 8 |
|  | Cox’s Bazar | 2014 | COX | 92.00773 | | 21.43946 | | 31 | 23 |
| **MIDDLE EAST** | | | | | | | | | |
| **Pakistan** | Chabahar | 2013 | PAK | 69.34512 | | 30.37532 | | 30 | 27 |
| **Armenia** | NA | 2003-2005 | ARM | 45.03819 | | 40.0691 | | 30 | 21 |
| **Azerbaijan** | Sabirabad, Imishli and Saatly | 2003 | AZE | 47.57693 | | 40.1431 | | 30 | 21 |
| **Iran** | Chabahar district and Pishin district | 2005-2012 | IRN | 53.68805 | | 32.42791 | | 30 | 17 |
| **Turkey** |  | 2003-2005 | TUR | 35.24332 | | 38.96375 | | 8 | 7 |
| **AFRICA** | | | | | | | | | |
| **Central African Republic + Cameroun + Togo** | NA | NA | AFR | NA | | NA | | 4 | 4 |
| **Ethiopia** | NA | 2006-2011 | ETH | 40.48967 | | 9.145 | | 54 | 25 |
| **Mauritania** | NA | 2007 | MRT | -10.94083 | | 21.00789 | | 51 | 33 |
| **Sudan** | New Halfa | 2013 | HLF | 35.59861 | | 15.32876 | | 25 | 19 |
|  | Khartoum | 2013-2015 | KHA | 32.5599 | | 15.50065 | | 99 | 76 |
| **AMERICA** | | | | | | | | | |
| **Mexico** | Southern | 2010-2013 | MEX | -102.5528 | | 23.6345 | | 37 | 30 |
| **Honduras** | Continental (Atlandita, Colon, Gracias A Dios) | NA | HND | -86.24191 | | 15.2 | | 48 | 30 |
|  | Bay Islands | NA | BAY | -86.42632 | | 16.37715 | | 12 | 9 |
| **Venezuela** | NA | 2003-2007 | VEN | -66.58973 | | 6.42375 | | 30 | 19 |
| **Peru** | Maynas, Iquitos | 2008-2014 | PER | -75.015152 | | -9.189967 | | 85 | 69 |
| **French Guiana** | Saint Georges de l’Oyapock | 2007 | STG | -51.807382 | | 3.892572 | | 47 | 43 |
|  | Cayenne | 2007 | CAY | -52.31345 | | 4.92242 | | 46 | 25 |
|  | Camopi | 2007 | CAM | -52.341382 | | 3.166021 | | 56 | 20 |
