# Supplementary material for: Human Plasmodium vivax diversity, population structure and evolutionary origin
Source: PLoS Negl Trop Dis. 2020 Mar 9;14(3):e0008072. doi: 10.1371/journal.pntd.0008072 (PMC7082039; doi:10.1371/journal.pntd.0008072)
Supplement: S2 Table — For markers on the same chromosome, distances are enough to limit any physical linkage: MS2, MS5 and MS6 are at a minimum at 0.15Mb apart from each others; MS7 and MS8 are 1.14Mb appart; MS12 and MS15 are 1.04Mb appart. (DOCX) [file pntd.0008072.s007.docx]

**Table S2.**

| **Code** | **Chromosome location (hypothetical protein code)** | **Allele size, bp** |
| --- | --- | --- |
| **MS1** | 3 (CM000444) | 222-249 |
| **MS2** | 6 (CM000447) | 161-269 |
| **MS3** | 4 (CM000445; Pv002815) | 179-206 |
| **MS4** | 6 (CM000447; Pv110815) | 188-245 |
| **MS5** | 6 (CM000447; Pv110955) | 164-218 |
| **MS6** | 11 (CM000452;  Pv113500) | 175-259 |
| **MS7** | 12 (CM00453; Pv116655) | 134-179 |
| **MS8** | 12 (CM00453; Pv118000) | 195-321 |
| **MS9** | 8 (CM000449; Pv119540) | 144-180 |
| **MS10** | 13 (CM00454; Pv084410) | 165-249 |
| **MS12** | 5 (CM000446; Pv088840) | 174-267 |
| **MS15** | 5 (CM000446; Pv090090) | 223-316 |
| **MS16** | 9 (CM00450; Pv092625) | 170-353 |
| **MS20** | 10 (CM000451; Pv097575) | 154-259 |
