# Supplementary material for: Human Plasmodium vivax diversity, population structure and evolutionary origin
Source: PLoS Negl Trop Dis. 2020 Mar 9;14(3):e0008072. doi: 10.1371/journal.pntd.0008072 (PMC7082039; doi:10.1371/journal.pntd.0008072)
Supplement: S3 Table — For microsatellite genotypes, blank cases correspond to the absence of readable amplification/genotype. (DOCX) [file pntd.0008072.s008.docx]

**Table S3.**

| Country | Locality | Sample ID | MS1 | MS2 | MS3 | MS4 | MS5 | MS6 | MS7 | MS8 | MS9 | MS10 | MS12 | MS15 | MS16 | MS20 |
| --- | --- | --- | --- | --- | --- | --- | --- | --- | --- | --- | --- | --- | --- | --- | --- | --- |
| Armenia | NA | ARM1 | 237 | 205 | 188 | 197 | 176 | 184 | 143 | 282 | 159 | 213 | 222 | 250 | 245 | 205 |
| Armenia | NA | ARM2 | 234 | 209 | 188 | 197 | 176 | 187 | 143 | 285 | 156 | 186 | 222 | 250 | 245 | 202 |
| Armenia | NA | ARM3 | 237 | 213 | 185 | 194 | 176 | 250 | 143 | 297 | 147 | 186 | 222 | 295 | 314 | 217 |
| Armenia | NA | ARM4 | 237 | 213 | 188 | 245 | 179 | 250 | 143 | 282 | 159 | 213 | 222 | 262 | 251 | 211 |
| Armenia | NA | ARM5 | 234 | 205 | 191 | 194 | 182 | 241 | 143 |  | 168 | 216 | 222 | 250 | 323 | 202 |
| Armenia | NA | ARM6 | 234 | 229 | 191 |  | 188 | 250 | 143 |  | 168 | 210 | 228 | 256 | 245 | 205 |
| Armenia | NA | ARM7 | 234 | 201 | 191 | 194 | 176 | 241 | 146 | 240 | 153 | 213 | 219 | 310 | 305 | 217 |
| Armenia | NA | ARM8 | 231 | 213 |  | 194 | 185 | 256 | 149 | 240 | 147 | 198 |  | 253 | 284 | 238 |
| Armenia | NA | ARM9 | 234 | 205 | 194 | 194 | 185 | 256 | 149 | 207 | 147 | 198 | 210 | 253 | 350 | 214 |
| Armenia | NA | ARM10 | 234 | 201 |  |  | 176 | 238 | 146 |  | 150 | 213 | 219 | 310 | 305 | 217 |
| Armenia | NA | ARM11 | 234 | 209 | 185 | 194 | 173 | 241 | 143 | 228 | 147 | 210 | 231 | 256 | 290 | 196 |
| Armenia | NA | ARM12 | 234 | 209 | 185 | 194 | 173 | 238 | 143 | 231 | 147 | 210 | 231 | 256 | 293 | 196 |
| Armenia | NA | ARM13 | 234 | 201 | 191 | 194 | 176 | 241 | 146 | 240 | 150 | 213 | 219 | 310 | 305 | 217 |
| Armenia | NA | ARM14 | 234 | 209 | 185 | 194 | 173 | 238 | 143 | 228 | 147 | 210 | 231 | 256 | 290 | 196 |
| Armenia | NA | ARM15 | 234 | 209 | 188 | 194 | 173 | 241 | 143 | 252 | 147 | 186 | 231 | 250 | 236 | 196 |
| Armenia | NA | ARM16 | 231 | 209 | 188 | 194 | 173 | 241 | 143 | 252 | 147 | 186 | 231 | 250 | 251 | 196 |
| Armenia | NA | ARM17 | 234 | 201 | 191 | 194 | 176 | 241 | 146 | 240 | 150 | 213 | 219 | 310 | 305 | 217 |
| Armenia | NA | ARM18 | 234 | 201 | 191 | 194 | 176 | 241 | 146 | 240 | 153 | 213 | 234 |  | 305 | 217 |
| Armenia | NA | ARM19 | 234 | 201 | 191 | 194 | 176 | 241 | 146 | 240 | 150 | 213 | 219 | 310 | 305 | 217 |
| Armenia | NA | ARM20 | 234 | 201 | 191 | 194 | 176 | 238 | 146 | 240 | 153 | 213 | 219 | 310 | 305 | 217 |
| Armenia | NA | ARM21 | 234 | 209 | 185 | 194 | 173 | 241 | 143 | 231 | 147 | 210 | 231 |  | 305 | 196 |
| Azerbaïdjan | SABIRABAD | AZBSAB1 | 237 | 201 | 191 | 194 | 176 | 238 | 143 | 243 | 150 | 204 | 219 | 310 | 308 | 217 |
| Azerbaïdjan | SABIRABAD | AZBSAB2 | 237 | 201 | 191 | 194 | 176 | 238 | 146 | 243 | 153 | 213 | 219 | 310 | 305 | 217 |
| Azerbaïdjan | SABIRABAD | AZBSAB3 | 237 | 201 | 191 | 194 | 167 | 238 | 146 | 243 | 150 | 213 | 210 | 310 | 305 | 217 |
| Azerbaïdjan | SABIRABAD | AZBSAB4 | 234 | 201 | 191 | 194 | 176 | 238 | 146 | 243 | 150 | 213 | 219 | 310 | 305 | 217 |
| Azerbaïdjan | SABIRABAD | AZBSAB5 | 234 | 201 | 191 | 194 | 176 | 238 | 143 | 240 | 153 | 204 | 219 | 310 | 308 | 217 |
| Azerbaïdjan | IMISHLI | AZBIM1 | 234 | 201 | 191 | 194 | 176 | 238 | 146 | 240 | 153 | 213 | 219 | 310 | 305 | 217 |
| Azerbaïdjan | IMISHLI | AZBIM2 | 234 | 201 | 191 | 194 | 176 | 238 | 146 | 240 | 153 | 213 | 219 | 310 | 305 | 217 |
| Azerbaïdjan | IMISHLI | AZBIM3 | 234 | 201 | 191 | 194 | 176 | 238 | 146 | 240 | 153 | 213 | 219 | 310 | 305 | 217 |
| Azerbaïdjan | IMISHLI | AZBIM4 | 237 | 201 | 191 | 194 | 176 | 238 | 146 | 240 | 153 | 213 | 219 | 350 | 305 | 217 |
| Azerbaïdjan | IMISHLI | AZBIM5 | 237 | 201 | 191 | 194 | 176 | 238 | 149 | 243 | 150 | 213 | 219 | 310 | 305 | 217 |
| Azerbaïdjan | MINGECHEVIR | AZBMIN1 | 243 | 221 | 197 | 194 | 173 | 238 | 137 | 249 | 162 | 186 | 225 | 253 | 293 | 178 |
| Azerbaïdjan | MINGECHEVIR | AZBMIN2 | 234 | 201 | 197 | 200 | 173 | 238 | 143 | 240 | 147 | 210 | 213 | 244 | 308 | 223 |
| Azerbaïdjan | MINGECHEVIR | AZBMIN3 | 234 | 201 | 191 | 194 | 176 | 238 | 146 | 240 | 150 | 213 | 219 | 316 | 305 | 217 |
| Azerbaïdjan | MINGECHEVIR | AZBMIN4 | 234 | 201 | 191 | 194 | 176 | 238 | 146 | 240 | 150 | 213 | 219 | 310 | 305 | 217 |
| Azerbaïdjan | MINGECHEVIR | AZBMIN5 | 234 | 221 | 188 | 197 | 176 | 238 | 137 | 219 | 156 | 186 | 213 | 253 | 290 | 172 |
| Azerbaïdjan | SAATLY | AZBSAA1 | 234 | 201 | 191 | 200 | 173 | 238 | 146 | 240 | 156 | 186 | 225 | 250 | 233 | 181 |
| Azerbaïdjan | SAATLY | AZBSAA2 | 234 | 201 | 191 | 194 | 176 | 238 | 146 | 243 | 150 | 213 | 219 | 310 | 305 | 217 |
| Azerbaïdjan | SAATLY | AZBSAA3 | 234 | 201 | 191 | 194 | 176 | 238 | 137 | 219 | 159 | 210 | 231 | 250 | 305 | 202 |
| Azerbaïdjan | BEYLOGAR | AZBBEY1 | 237 | 209 | 197 | 194 | 173 | 238 | 137 | 219 | 159 | 186 | 231 | 250 | 308 | 196 |
| Azerbaïdjan | BEYLOGAR | AZBBEY2 | 234 | 201 | 191 | 200 | 173 | 238 | 146 | 231 | 165 | 186 | 219 | 250 | 308 | 196 |
| Azerbaïdjan | BEYLOGAR | AZBBEY3 | 234 | 201 | 191 | 194 | 176 | 238 | 146 | 240 | 153 | 213 | 219 | 310 | 305 | 217 |
| Bangladesh | Cox's Bazar | BGLDSL1 | 237 |  | 194 | 206 | 188 | 250 | 149 | 228 | 159 | 213 | 213 | 247 | 242 | 208 |
| Bangladesh | Cox's Bazar | BGLDSL2 | 231 | 213 | 191 | 194 | 176 | 244 | 155 | 228 | 147 | 201 | 213 | 247 | 242 | 217 |
| Bangladesh | Cox's Bazar | BGLDSL3 | 237 | 225 | 188 | 206 | 173 | 187 | 146 | 237 | 156 | 180 | 210 | 277 | 266 | 199 |
| Bangladesh | Cox's Bazar | BGLDSL4 | 231 | 181 | 191 | 218 | 191 | 184 | 152 | 309 | 156 | 180 | 216 | 241 | 332 | 220 |
| Bangladesh | Cox's Bazar | BGLDSL5 | 234 | 241 | 200 | 203 | 182 | 250 | 143 | 267 | 159 | 225 | 210 | 259 | 251 | 211 |
| Bangladesh | Cox's Bazar | BGLDSL6 | 231 | 181 | 194 | 200 | 188 | 247 | 155 | 243 | 168 | 180 | 210 | 238 | 329 | 220 |
| Bangladesh | Cox's Bazar | BGLDSL7 | 237 | 221 | 197 | 188 | 167 | 226 | 143 | 300 | 162 | 195 | 228 | 262 | 197 | 208 |
| Bangladesh | Cox's Bazar | BGLDSL8 | 237 | 217 | 200 | 197 | 206 | 241 | 161 | 297 | 159 | 204 | 219 | 307 | 278 | 205 |
| Bangladesh | Khagrachari | BGLDKM1 | 234 | 217 | 191 | 209 | 173 | 229 | 143 | 270 | 162 | 198 | 210 | 244 | 248 | 196 |
| Bangladesh | Khagrachari | BGLDKM2 | 225 | 233 | 188 | 209 | 176 | 229 | 140 | 258 | 165 | 228 | 210 | 241 | 278 | 214 |
| Bangladesh | Cox's Bazar | BGLDSL9 | 231 |  | 188 | 206 | 176 | 244 | 152 | 213 | 159 | 198 | 228 | 250 | 299 | 205 |
| Bangladesh | Cox's Bazar | BGLDSL10 | 234 | 189 | 185 | 203 | 173 | 211 | 155 | 225 | 159 | 201 | 216 | 244 | 209 | 214 |
| Bangladesh | Cox's Bazar | BGLDSL11 | 231 | 229 | 191 | 212 | 173 | 250 | 158 | 255 | 150 | 207 | 210 | 262 | 284 | 193 |
| Bangladesh | Khagrachari | BGLDKM3 | 234 | 189 | 191 | 206 | 197 | 241 | 143 | 252 | 156 | 207 | 210 | 244 | 263 | 214 |
| Bangladesh | Khagrachari | BGLDKM4 | 234 | 225 | 191 | 197 | 173 | 250 | 137 | 282 | 162 | 201 | 216 | 256 | 278 | 211 |
| Bangladesh | Cox's Bazar | BGLDSL12 | 234 | 205 | 191 | 197 | 179 | 250 | 143 | 267 | 153 | 204 | 213 | 247 | 281 | 208 |
| Bangladesh | Cox's Bazar | BGLDSL13 | 234 | 209 | 191 | 203 | 188 | 241 | 146 | 285 | 162 | 210 | 210 | 247 | 266 | 202 |
| Bangladesh | Cox's Bazar | BGLDSL14 | 231 | 213 | 191 | 206 | 179 | 247 | 152 | 237 | 150 | 219 | 234 | 247 | 290 | 217 |
| Bangladesh | Cox's Bazar | BGLDSL15 | 237 | 213 | 200 | 203 | 188 | 247 | 155 |  | 150 | 213 | 210 | 250 | 266 | 199 |
| Bangladesh | Cox's Bazar | BGLDSL16 | 234 | 221 | 191 | 212 | 200 | 247 | 152 | 279 | 162 | 207 | 210 | 241 | 284 | 205 |
| Bangladesh | Cox's Bazar | BGLDSL17 | 237 | 197 | 197 | 206 | 176 | 256 | 149 | 219 | 144 | 207 | 204 | 244 | 302 | 199 |
| Bangladesh | Cox's Bazar | BGLDSL18 | 234 | 217 | 194 | 212 | 185 | 241 | 146 | 246 | 147 | 228 | 183 | 247 | 281 | 202 |
| Bangladesh | Cox's Bazar | BGLDSL19 | 231 | 205 | 197 | 206 | 173 | 256 | 146 | 261 | 165 | 201 | 210 | 241 | 311 | 211 |
| Bangladesh | Cox's Bazar | BGLDSL20 | 237 | 209 | 191 | 203 | 176 | 244 | 143 | 255 | 153 | 192 | 219 | 241 | 287 | 202 |
| Bangladesh | Cox's Bazar | BGLDSL21 | 228 | 197 | 191 | 200 | 173 | 235 | 146 | 267 | 162 | 210 | 210 | 244 | 293 | 208 |
| Bangladesh | Cox's Bazar | BGLDSL22 | 237 | 209 | 194 | 203 | 188 | 247 | 164 | 279 | 162 | 198 | 210 | 253 | 263 | 211 |
| Bangladesh | Cox's Bazar | BGLDSL23 | 237 | 205 | 188 | 188 | 182 | 253 | 152 | 267 | 159 | 201 | 210 | 250 | 269 | 208 |
| Bangladesh | Khagrachari | BGLDKM5 | 231 | 205 | 191 | 200 | 188 | 250 | 143 | 252 | 159 | 204 | 216 | 274 | 293 | 214 |
| Bangladesh | Khagrachari | BGLDKM6 | 234 | 177 | 191 | 209 | 176 | 244 | 161 | 258 | 165 | 195 | 210 | 247 | 281 | 220 |
| Bangladesh | Khagrachari | BGLDKM7 | 234 | 181 | 191 | 200 | 173 | 184 | 152 | 273 | 159 | 207 | 210 | 247 | 245 | 208 |
| Bangladesh | Khagrachari | BGLDKM8 | 234 | 253 | 191 | 212 | 188 | 235 | 146 | 252 | 171 | 216 | 216 | 250 | 272 | 208 |
| Bangladesh | Bandarban | BGLDSL24 | 234 | 225 | 191 | 200 | 191 | 247 | 155 | 258 | 180 | 189 | 222 | 244 | 311 | 211 |
| Bangladesh | Bandarban | BGLDSL25 | 237 | 213 | 194 | 200 | 176 | 241 | 149 | 261 | 156 | 201 | 216 | 250 | 302 | 220 |
| Bangladesh | Bandarban | BGLDSL26 | 228 | 205 | 206 | 197 | 167 | 247 | 155 | 261 | 156 | 177 | 204 | 247 | 347 | 226 |
| Bangladesh | Bandarban | BGLDSL27 | 228 | 205 | 206 |  | 167 | 247 | 155 | 261 | 156 | 177 | 204 | 247 | 347 | 226 |
| Bangladesh | Bandarban | BGLDSL28 | 228 | 217 | 206 | 197 | 173 | 247 | 155 | 261 | 156 | 207 | 204 | 247 | 347 | 226 |
| Bangladesh | Bandarban | BGLDSL29 | 234 | 201 | 194 | 203 | 176 | 244 | 155 | 237 | 156 | 192 |  | 256 | 302 | 205 |
| Bangladesh | Bandarban | BGLDSL30 | 234 | 217 | 191 | 212 | 188 | 232 | 155 | 261 | 153 | 225 | 210 | 241 | 272 | 223 |
| Bangladesh | Bandarban | BGLDSL31 | 231 | 197 | 191 | 212 | 188 | 241 | 161 | 321 | 159 | 195 | 210 | 253 | 227 | 211 |
| Bangladesh | Bandarban | BGLDSL32 | 234 | 205 | 197 | 203 | 179 | 250 | 152 | 285 | 156 | 207 | 210 | 241 | 260 | 220 |
| Bangladesh | Bandarban | BGLDSL33 | 234 | 205 | 197 |  | 185 | 232 | 146 | 282 | 159 | 189 | 234 | 241 | 299 | 226 |
| Bangladesh | Bandarban | BGLDSL34 | 237 | 197 | 191 | 200 |  | 208 | 146 | 291 | 159 | 231 | 216 | 256 | 233 | 214 |
| Bangladesh | Bandarban | BGLDSL35 | 231 | 225 | 197 | 200 | 179 | 256 | 140 | 282 | 153 | 192 | 225 | 241 | 227 | 199 |
| Bangladesh | Bandarban | BGLDSL36 | 237 | 205 | 200 | 197 | 179 | 247 | 152 | 258 | 162 | 207 | 213 | 253 | 230 | 202 |
| Cameroun | NA | CAM1 | 234 | 201 | 191 | 206 | 173 | 226 | 143 | 237 | 162 | 183 |  | 247 | 239 | 199 |
| Cameroun | NA | CAM2 | 234 | 217 | 191 | 206 | 173 | 226 | 143 | 237 | 162 | 183 |  | 247 | 239 | 199 |
| Central African Region | NA | CAR | 234 | 213 | 191 | 200 | 176 | 241 | 143 | 237 | 159 | 183 | 210 | 247 | 287 | 181 |
| Ethiopia | NA | H5 | 228 |  |  | 197 | 179 | 184 | 143 | 282 | 150 | 213 | 237 | 250 | 314 | 226 |
| Ethiopia | NA | H6 | 234 |  | 188 | 200 | 173 | 253 | 146 | 270 | 171 | 216 | 222 |  | 245 | 211 |
| Ethiopia | NA | H8 | 234 |  | 191 |  | 173 | 253 | 143 | 270 | 171 | 216 | 210 |  |  | 211 |
| Ethiopia | NA | H9 | 240 |  | 191 |  | 179 | 232 | 143 | 288 | 150 |  | 210 |  |  | 223 |
| Ethiopia | NA | H10 | 228 |  | 188 | 245 | 182 | 241 | 146 | 207 | 156 | 189 | 210 |  | 245 | 205 |
| Ethiopia | NA | H11 | 228 |  |  | 245 | 182 | 184 | 143 | 207 | 156 | 189 | 210 | 256 | 245 | 205 |
| Ethiopia | NA | H13 | 228 |  | 188 | 245 | 182 | 241 |  | 207 | 156 | 189 | 210 | 256 | 245 | 205 |
| Ethiopia | NA | H14 | 228 |  | 188 | 245 | 182 | 241 | 143 | 207 | 156 | 189 | 210 | 256 | 245 | 205 |
| Ethiopia | NA | H15 | 240 |  |  | 206 | 179 | 232 | 143 | 288 | 150 | 207 | 210 |  |  | 223 |
| Ethiopia | NA | H17 | 234 | 181 |  |  | 179 | 250 | 143 | 204 | 174 | 219 | 225 | 256 | 287 | 205 |
| Ethiopia | NA | H18 | 228 | 181 | 188 | 245 | 182 | 241 | 143 | 207 | 156 | 189 | 210 | 256 | 245 | 205 |
| Ethiopia | NA | H19 | 228 |  | 188 | 245 | 182 | 241 | 143 | 207 | 156 | 189 | 210 | 256 | 245 | 205 |
| Ethiopia | NA | H20 | 228 | 181 |  | 245 | 182 |  | 146 | 207 | 156 | 189 | 210 |  | 245 | 205 |
| Ethiopia | NA | H21 | 228 | 181 | 188 | 245 | 182 | 241 | 143 | 207 | 156 | 189 | 210 | 256 | 245 | 205 |
| Ethiopia | NA | H22 | 228 | 181 | 188 | 245 | 182 | 241 | 146 | 207 | 156 | 189 | 210 | 256 | 245 | 205 |
| Ethiopia | NA | H23 | 228 | 181 | 188 | 245 | 182 | 241 | 143 | 207 | 156 | 189 | 210 | 256 | 245 | 205 |
| Ethiopia | NA | J13 | 231 | 205 |  | 194 | 179 |  | 143 |  | 150 |  | 225 | 271 | 245 |  |
| Ethiopia | NA | J15 | 237 | 209 | 188 |  | 176 | 247 | 143 |  | 156 | 219 | 234 | 247 | 251 |  |
| Ethiopia | NA | J16 | 240 | 213 | 185 | 197 | 179 | 250 | 143 | 231 | 150 | 219 | 210 | 262 | 245 |  |
| Ethiopia | NA | J17 | 234 | 209 |  | 197 | 176 |  | 146 | 231 | 150 | 183 | 210 | 268 | 245 | 223 |
| Ethiopia | NA | J20 | 237 | 209 | 197 |  | 179 | 247 | 143 |  | 150 |  | 225 | 262 | 260 | 223 |
| Ethiopia | NA | ETHIO1 | 234 | 229 | 191 |  | 188 | 250 | 143 |  | 165 | 210 | 228 | 256 | 245 | 205 |
| Ethiopia | NA | ETHIO2 | 234 | 205 | 191 | 197 | 182 | 238 | 143 |  | 165 | 216 | 222 | 250 | 326 | 202 |
| Ethiopia | NA | ETHIO3 | 237 | 213 | 188 |  | 179 | 250 | 143 | 282 | 156 | 213 | 222 | 262 | 251 | 211 |
| Ethiopia | NA | ETHIO4 | 237 | 213 | 185 | 194 | 176 | 247 | 143 | 297 | 147 | 186 | 222 | 295 | 314 | 217 |
| French Guyana | Saint Georges | GFSTG1 | 237 | 201 | 191 | 197 | 176 | 235 | 143 | 264 | 165 | 195 | 216 | 244 | 299 | 205 |
| French Guyana | Saint Georges | GFSTG2 | 237 | 201 | 191 | 197 | 179 | 235 | 143 | 264 | 165 | 195 | 210 | 244 | 299 | 205 |
| French Guyana | Saint Georges | GFSTG3 | 234 | 209 | 188 | 206 | 188 | 208 | 143 | 255 | 168 | 195 | 267 | 241 | 287 | 205 |
| French Guyana | Saint Georges | GFSTG4 | 237 | 201 | 191 | 197 | 179 | 235 | 143 | 255 | 168 | 234 | 210 | 244 | 296 | 202 |
| French Guyana | Saint Georges | GFSTG5 | 237 | 201 | 191 | 197 | 179 | 235 | 143 | 258 | 168 | 234 | 210 | 244 | 299 | 202 |
| French Guyana | Saint Georges | GFSTG6 | 237 | 197 | 188 | 194 | 188 | 241 | 155 | 243 | 159 | 222 | 258 | 241 | 269 | 205 |
| French Guyana | Saint Georges | GFSTG7 | 237 | 197 | 188 | 197 | 191 | 235 | 143 | 255 | 159 | 207 | 261 | 241 | 287 | 226 |
| French Guyana | Saint Georges | GFSTG8 | 237 | 201 | 191 | 197 | 179 | 235 | 143 | 264 | 165 | 195 | 210 | 244 | 299 | 205 |
| French Guyana | Saint Georges | GFSTG9 | 237 | 201 | 194 | 197 | 176 | 235 | 143 | 264 | 165 | 195 | 216 | 292 | 299 | 205 |
| French Guyana | Saint Georges | GFSTG10 | 234 | 201 | 191 | 197 | 176 | 235 | 143 | 264 | 165 | 195 | 216 | 265 |  | 205 |
| French Guyana | Saint Georges | GFSTG11 | 237 | 201 | 194 | 197 | 179 | 235 | 143 | 297 | 171 | 234 | 210 | 244 | 299 | 202 |
| French Guyana | Saint Georges | GFSTG12 | 234 | 213 | 188 | 194 | 170 | 235 | 143 | 222 | 159 | 213 | 216 | 235 | 293 | 199 |
| French Guyana | Saint Georges | GFSTG13 | 237 | 197 | 191 | 194 | 185 | 235 | 155 | 252 | 162 | 195 | 216 | 241 | 251 | 202 |
| French Guyana | Saint Georges | GFSTG14 | 231 | 197 | 191 | 200 | 200 | 235 | 143 | 222 | 171 | 207 | 222 | 250 | 305 | 196 |
| French Guyana | Saint Georges | GFSTG15 | 237 | 197 | 194 | 194 | 200 | 241 | 143 | 243 | 162 | 222 | 222 | 292 | 251 | 205 |
| French Guyana | Saint Georges | GFSTG16 | 237 | 201 | 194 | 197 | 179 | 235 | 143 | 264 | 165 | 195 | 210 | 244 | 299 | 205 |
| French Guyana | Saint Georges | GFSTG17 | 237 | 201 | 194 | 200 | 179 | 235 | 143 | 297 | 168 | 234 | 210 | 244 | 299 | 202 |
| French Guyana | Saint Georges | GFSTG18 | 237 | 201 | 194 | 197 | 179 | 235 | 143 | 264 | 165 | 195 | 210 | 244 | 299 | 205 |
| French Guyana | Saint Georges | GFSTG19 | 234 | 233 | 191 | 194 | 173 | 235 | 155 | 222 | 159 | 195 | 210 | 259 | 221 | 211 |
| French Guyana | Saint Georges | GFSTG20 | 234 | 201 | 191 | 200 | 179 | 235 | 143 | 264 | 165 | 195 | 210 | 235 |  | 205 |
| French Guyana | Saint Georges | GFSTG21 | 234 | 201 | 191 | 197 | 179 | 238 | 143 | 264 | 165 | 195 | 210 | 235 |  | 205 |
| French Guyana | Saint Georges | GFSTG22 | 234 | 209 | 191 | 203 | 188 | 208 | 143 | 255 | 168 | 195 | 267 | 241 |  | 205 |
| French Guyana | Saint Georges | GFSTG23 | 234 | 209 | 191 | 203 | 188 | 208 | 143 | 255 | 168 | 195 | 267 | 241 |  | 205 |
| French Guyana | Saint Georges | GFSTG24 | 237 | 201 | 194 | 197 | 179 | 235 | 143 | 297 | 168 | 234 | 210 | 244 | 299 | 202 |
| French Guyana | Saint Georges | GFSTG25 | 237 | 201 | 194 | 197 | 179 | 235 | 143 | 297 | 168 | 234 | 210 | 244 | 299 | 202 |
| French Guyana | Saint Georges | GFSTG26 | 237 | 201 | 194 | 197 | 179 | 235 | 143 | 297 | 168 | 234 | 210 | 244 | 299 | 202 |
| French Guyana | Saint Georges | GFSTG27 | 237 | 201 | 194 | 197 | 179 | 235 | 143 | 297 | 168 | 234 | 210 | 244 | 299 | 202 |
| French Guyana | Saint Georges | GFSTG28 | 237 | 197 | 188 | 197 | 200 | 235 | 143 | 222 | 168 | 234 | 174 | 241 | 254 | 205 |
| French Guyana | Saint Georges | GFSTG29 | 237 | 201 | 194 | 197 | 179 | 235 | 143 | 264 | 165 | 195 | 210 | 244 | 299 | 205 |
| French Guyana | Saint Georges | GFSTG30 | 237 | 201 | 194 | 197 | 179 | 235 | 143 | 297 | 168 | 234 | 210 | 244 | 299 | 202 |
| French Guyana | Saint Georges | GFSTG31 | 237 | 197 | 194 | 194 | 200 | 241 | 143 | 243 | 162 | 222 | 213 | 244 | 251 | 202 |
| French Guyana | Saint Georges | GFSTG32 | 237 | 197 | 194 | 194 | 200 | 241 | 143 | 243 | 162 | 222 | 213 | 244 | 251 | 202 |
| French Guyana | Saint Georges | GFSTG33 | 234 | 209 | 191 | 203 | 188 | 208 | 143 | 255 | 168 | 195 | 267 | 241 | 257 | 205 |
| French Guyana | Saint Georges | GFSTG34 | 237 | 201 | 194 | 197 | 179 | 235 | 143 | 297 | 168 | 234 | 210 | 244 | 299 | 202 |
| French Guyana | Saint Georges | GFSTG35 | 237 | 237 | 191 | 194 | 194 | 229 | 143 | 273 | 147 | 234 | 261 | 241 | 338 | 202 |
| French Guyana | Saint Georges | GFSTG36 | 237 | 213 | 194 | 209 | 188 | 235 | 155 | 273 | 165 | 198 | 216 | 235 |  | 208 |
| French Guyana | Saint Georges | GFSTG37 | 237 | 201 | 194 | 197 | 179 | 235 | 143 | 297 | 168 | 234 | 210 | 244 | 299 | 202 |
| French Guyana | Saint Georges | GFSTG38 | 237 | 201 | 194 | 197 | 179 | 235 | 143 | 297 | 168 | 234 | 210 | 244 | 299 | 202 |
| French Guyana | Saint Georges | GFSTG39 | 237 | 201 | 194 | 197 | 179 | 235 | 143 | 297 | 168 | 234 | 210 | 244 | 299 | 202 |
| French Guyana | Saint Georges | GFSTG40 | 231 | 209 | 191 | 203 | 188 | 208 | 143 | 255 | 168 | 195 | 267 | 241 |  | 205 |
| French Guyana | Saint Georges | GFSTG41 | 237 | 201 | 194 | 197 | 179 | 235 | 143 | 264 | 165 | 195 | 210 | 244 | 299 | 205 |
| French Guyana | Saint Georges | GFSTG42 | 237 | 201 | 194 | 197 | 179 | 235 | 143 | 264 | 165 | 195 | 210 | 244 | 299 | 205 |
| French Guyana | Saint Georges | GFSTG43 | 234 | 197 | 191 | 197 | 182 | 238 | 143 | 240 | 168 | 207 | 210 | 265 | 299 | 238 |
| French Guyana | Cayenne | GFCAY1 | 237 | 233 | 191 | 194 | 194 | 229 | 143 | 273 | 147 | 234 | 261 | 241 | 338 | 202 |
| French Guyana | Cayenne | GFCAY2 | 237 | 233 | 191 | 194 | 194 | 229 | 143 | 273 | 147 | 234 | 261 | 241 | 338 | 202 |
| French Guyana | Cayenne | GFCAY3 | 237 | 233 |  |  | 194 | 184 |  | 228 | 147 | 234 | 213 | 232 | 338 | 202 |
| French Guyana | Cayenne | GFCAY4 | 237 | 233 | 191 | 194 | 185 | 241 | 143 | 219 | 159 | 231 | 231 | 241 |  | 208 |
| French Guyana | Cayenne | GFCAY5 | 234 | 197 | 191 | 197 | 194 | 208 | 146 | 210 | 171 | 237 | 231 | 259 | 296 | 241 |
| French Guyana | Cayenne | GFCAY6 | 234 | 193 | 191 | 200 | 188 | 235 | 143 | 222 | 171 | 207 | 210 | 259 | 269 | 205 |
| French Guyana | Cayenne | GFCAY7 | 237 | 225 | 194 | 212 | 185 | 235 | 155 | 252 | 165 | 195 | 216 | 241 | 305 | 205 |
| French Guyana | Cayenne | GFCAY8 | 237 | 209 |  |  | 188 | 235 | 143 | 225 | 162 | 207 | 210 | 241 |  | 205 |
| French Guyana | Cayenne | GFCAY9 | 237 | 233 | 194 | 200 | 188 | 235 | 143 | 249 | 171 |  | 210 | 241 | 251 | 202 |
| French Guyana | Cayenne | GFCAY10 | 237 | 233 | 191 | 194 | 185 | 241 | 143 | 219 | 159 | 234 | 231 | 241 |  | 208 |
| French Guyana | Cayenne | GFCAY11 | 237 | 233 | 191 | 194 | 185 | 241 | 143 | 219 | 159 | 231 | 231 | 241 |  | 208 |
| French Guyana | Cayenne | GFCAY12 | 234 | 193 | 191 | 200 | 188 | 235 | 143 | 222 | 171 | 207 | 210 | 259 | 269 | 205 |
| French Guyana | Cayenne | GFCAY13 | 237 | 201 | 191 | 197 | 179 | 235 | 143 | 264 | 165 | 195 | 210 | 235 |  | 205 |
| French Guyana | Cayenne | GFCAY14 | 234 | 193 | 194 | 197 |  | 235 | 143 | 303 | 159 | 198 | 261 | 250 | 305 | 220 |
| French Guyana | Cayenne | GFCAY15 | 237 | 209 | 191 | 218 |  | 208 | 143 | 261 | 159 | 237 | 216 | 235 | 293 | 217 |
| French Guyana | Cayenne | GFCAY16 | 237 | 233 | 191 | 194 | 194 | 229 | 143 | 273 | 147 | 234 | 261 | 241 | 338 | 202 |
| French Guyana | Cayenne | GFCAY17 | 234 | 201 | 191 | 197 | 179 | 235 | 143 | 264 | 165 | 195 | 210 | 235 |  | 205 |
| French Guyana | Cayenne | GFCAY18 | 237 | 233 | 191 | 194 | 194 | 229 | 143 | 273 | 147 | 234 | 261 | 241 | 338 | 202 |
| French Guyana | Cayenne | GFCAY19 | 237 | 225 | 194 | 212 | 185 | 235 | 155 | 252 | 165 | 195 | 216 | 241 | 305 | 205 |
| French Guyana | Cayenne | GFCAY20 | 237 | 193 | 194 | 194 | 176 | 235 | 143 | 243 | 165 | 189 | 210 | 235 | 314 | 208 |
| French Guyana | Cayenne | GFCAY21 | 237 | 209 | 191 | 197 | 197 | 235 | 155 | 210 | 156 | 237 |  | 241 | 338 | 214 |
| French Guyana | Cayenne | GFCAY22 | 237 | 209 | 188 | 197 | 194 | 235 | 155 | 222 | 159 | 231 | 213 | 241 | 251 | 205 |
| French Guyana | Cayenne | GFCAY23 | 237 | 233 | 191 | 194 | 194 | 229 | 143 | 273 | 147 | 234 | 261 | 241 | 338 | 202 |
| French Guyana | Cayenne | GFCAY24 | 237 | 201 | 194 | 197 | 179 | 235 | 143 | 264 | 165 | 195 | 210 | 244 | 299 | 205 |
| French Guyana | Cayenne | GFCAY25 | 231 | 209 |  |  | 185 | 235 |  | 210 | 171 | 222 | 210 | 250 | 296 | 238 |
| French Guyana | Camopi | GFCAM1 |  | 193 | 191 | 194 | 182 | 211 | 155 | 243 | 159 | 231 | 213 | 247 | 338 | 208 |
| French Guyana | Camopi | GFCAM2 | 243 | 193 | 188 | 200 | 188 | 235 | 155 | 222 | 159 | 234 | 267 | 265 |  | 205 |
| French Guyana | Camopi | GFCAM3 | 237 | 197 | 194 | 206 | 173 | 235 | 155 | 210 | 156 | 207 | 210 | 241 | 308 | 217 |
| French Guyana | Camopi | GFCAM4 | 231 | 201 | 191 | 194 | 200 | 241 | 143 | 243 | 165 | 195 | 216 | 244 | 251 | 214 |
| French Guyana | Camopi | GFCAM5 | 243 | 193 | 188 | 200 | 188 | 235 | 155 | 222 | 159 | 231 | 267 | 259 |  | 205 |
| French Guyana | Camopi | GFCAM6 | 243 | 193 | 191 | 200 | 176 | 235 | 143 | 222 | 159 | 234 | 210 | 259 |  | 205 |
| French Guyana | Camopi | GFCAM7 | 243 | 193 | 191 | 200 | 188 | 235 | 155 | 222 | 159 | 231 | 210 | 250 |  | 205 |
| French Guyana | Camopi | GFCAM8 | 237 | 197 | 191 | 197 | 179 | 241 | 155 | 240 | 162 | 207 | 216 | 268 | 296 | 205 |
| French Guyana | Camopi | GFCAM9 | 237 | 197 | 191 | 197 | 179 | 241 | 155 | 240 | 162 | 207 | 216 | 268 | 296 | 205 |
| French Guyana | Camopi | GFCAM10 | 246 | 197 | 197 | 197 | 182 | 232 | 143 | 300 | 159 | 231 | 216 | 247 | 329 | 205 |
| French Guyana | Camopi | GFCAM11 | 234 | 201 | 188 | 194 | 188 | 241 | 143 | 210 | 162 | 213 | 216 | 241 | 284 | 238 |
| French Guyana | Camopi | GFCAM12 | 240 | 193 | 188 | 194 | 188 | 208 | 143 | 243 | 159 | 234 | 213 | 241 | 221 | 202 |
| French Guyana | Camopi | GFCAM13 | 234 | 233 | 191 | 194 | 182 | 235 | 143 | 210 | 171 | 198 | 216 |  |  | 199 |
| French Guyana | Camopi | GFCAM14 | 237 | 225 | 188 | 194 | 188 | 208 | 143 | 243 | 147 | 231 | 213 | 241 | 341 | 238 |
| French Guyana | Camopi | GFCAM15 | 231 | 197 | 188 | 194 | 188 | 241 | 155 | 249 | 156 | 207 | 255 | 235 | 299 | 211 |
| French Guyana | Camopi | GFCAM16 | 237 | 197 | 194 | 191 | 197 | 241 | 143 | 210 | 174 | 222 | 216 | 250 | 221 | 208 |
| French Guyana | Camopi | GFCAM17 | 237 | 193 | 188 | 200 | 197 | 232 | 143 | 243 | 159 | 231 | 228 | 241 |  | 205 |
| French Guyana | Camopi | GFCAM18 | 237 | 197 | 191 | 197 | 179 |  | 143 | 240 | 162 | 207 | 267 | 244 | 269 | 205 |
| French Guyana | Camopi | GFCAM19 | 237 | 197 | 188 | 194 | 188 | 235 | 143 | 222 | 159 | 207 | 210 | 259 | 269 | 214 |
| French Guyana | Camopi | GFCAM20 | 237 | 225 | 188 | 194 | 188 | 208 | 143 | 243 | 147 | 231 | 213 | 241 | 341 | 238 |
| Honduras | Atlantida | HDRAtl1 | 243 | 205 | 188 | 200 | 179 | 232 | 143 | 276 | 156 | 237 | 183 | 256 | 287 | 256 |
| Honduras | Atlantida | HDRAtl2 | 237 | 225 | 185 | 200 | 182 | 247 | 143 | 273 |  | 231 | 210 | 247 | 236 | 196 |
| Honduras | Atlantida | HDRAtl3 | 234 | 229 | 188 | 200 | 176 | 229 | 143 | 279 | 162 | 222 | 213 | 253 |  | 220 |
| Honduras | Atlantida | HDRAtl4 | 234 | 241 | 188 | 200 | 188 | 235 | 146 | 273 |  | 225 | 187 | 253 | 299 | 178 |
| Honduras | Colon | HDRCol1 | 237 | 181 | 188 | 194 | 179 | 232 | 143 | 279 | 168 | 234 | 219 | 253 |  | 187 |
| Honduras | Colon | HDRCol2 | 237 | 181 |  |  | 179 | 247 | 143 | 276 | 177 | 237 | 183 | 256 | 305 | 217 |
| Honduras | Colon | HDRCol3 | 234 | 165 |  | 203 | 182 | 229 | 143 | 240 |  | 249 | 210 | 259 | 266 | 235 |
| Honduras | Colon | HDRCol4 | 234 | 225 |  | 203 | 182 | 187 | 146 | 240 |  | 246 | 210 | 259 | 266 | 235 |
| Honduras | Colon | HDRCol5 | 234 | 225 | 188 | 203 | 182 | 232 | 143 | 240 |  | 237 | 210 | 259 | 266 | 235 |
| Honduras | Colon | HDRCol6 | 225 | 161 |  | 203 | 182 | 175 | 155 | 228 |  | 234 | 210 | 253 | 251 | 235 |
| Honduras | Colon | HDRCol7 | 225 | 181 | 188 | 200 | 179 | 232 | 143 | 213 | 165 | 234 | 183 | 262 | 284 | 196 |
| Honduras | Colon | HDRCol8 | 237 | 181 | 188 | 200 | 179 | 232 | 143 | 213 | 165 | 234 | 183 | 262 | 284 | 196 |
| Honduras | Gracias A Dios | HDR1Gad1 | 228 | 181 | 188 | 191 | 179 | 235 | 158 | 234 | 171 | 225 | 183 |  | 239 | 211 |
| Honduras | Gracias A Dios | HDR1Gad2 | 231 |  | 197 | 200 | 173 | 178 |  | 213 | 162 | 210 |  |  | 221 | 199 |
| Honduras | Gracias A Dios | HDR1Gad3 | 237 | 205 | 188 | 200 | 173 | 232 | 143 | 219 | 165 | 216 | 201 | 241 | 353 | 187 |
| Honduras | Gracias A Dios | HDR1Gad4 | 231 | 181 | 188 |  |  | 250 | 143 |  |  | 237 | 207 |  | 254 | 184 |
| Honduras | Gracias A Dios | HDR1Gad5 | 228 | 209 | 197 | 218 | 179 | 175 | 158 | 270 | 162 | 234 | 183 | 247 | 182 | 217 |
| Honduras | Gracias A Dios | HDR1Gad6 | 228 |  |  |  | 179 | 175 | 143 | 204 | 162 | 198 |  |  | 248 | 232 |
| Honduras | Gracias A Dios | HDR1Gad7 | 237 | 205 | 188 | 200 | 179 | 178 | 143 | 213 | 162 | 222 | 183 | 256 | 191 | 223 |
| Honduras | Bay Islands | HDRBayIs1 | 237 | 181 | 188 | 194 | 182 | 238 | 155 | 228 | 159 | 186 | 183 | 247 | 317 | 205 |
| Honduras | Bay Islands | HDRBayIs2 | 225 | 181 | 188 | 194 | 182 | 238 | 158 | 228 | 159 | 237 | 183 | 247 | 317 | 205 |
| Honduras | Bay Islands | HDRBayIs3 | 234 | 209 | 188 | 203 | 182 | 175 | 143 | 213 | 165 | 243 | 216 | 256 | 353 | 187 |
| Honduras | Bay Islands | HDRBayIs4 | 228 | 185 | 188 | 194 | 182 | 247 | 155 |  | 156 | 237 | 231 | 256 | 260 | 235 |
| Honduras | Bay Islands | HDRBayIs5 | 237 | 181 | 188 | 200 | 179 | 235 | 143 | 213 | 165 | 237 | 183 | 256 | 338 | 205 |
| Honduras | Bay Islands | HDRBayIs6 | 225 | 181 | 188 | 203 | 179 | 235 | 143 | 213 | 165 | 237 | 183 | 244 | 317 | 205 |
| Honduras | Bay Islands | HDRBayIs7 | 222 | 245 | 188 | 194 |  | 238 | 158 | 228 | 159 | 237 |  | 247 |  | 205 |
| Honduras | Bay Islands | HDRBayIs8 | 222 | 233 | 188 | 200 | 167 | 229 | 143 | 279 | 165 | 222 | 213 | 241 |  | 220 |
| Honduras | Bay Islands | HDRBayIs9 | 222 | 245 | 188 | 194 | 182 | 235 | 155 | 228 | 159 | 228 | 183 | 247 | 317 | 205 |
| Honduras | Olancho | HDROlan1 | 237 | 213 | 188 | 200 | 182 | 181 | 155 | 234 | 159 | 180 | 183 | 244 |  | 202 |
| Honduras | Olancho | HDROlan2 | 228 | 213 | 188 | 200 | 173 | 181 | 158 | 234 | 159 | 243 |  | 244 | 317 | 202 |
| Honduras | Olancho | HDROlan3 | 237 | 181 | 185 | 200 | 179 | 253 | 143 | 276 | 177 | 237 | 183 | 256 | 287 | 217 |
| Honduras | Olancho | HDROlan4 | 225 | 213 | 188 | 200 | 182 | 178 | 155 | 234 | 159 |  | 183 | 244 |  | 199 |
| Honduras | Olancho | HDROlan5 | 228 | 181 | 185 | 200 | 179 | 175 | 143 | 261 | 177 | 237 | 183 | 256 | 305 | 217 |
| Honduras | Olancho | HDROlan6 | 222 | 181 | 185 | 194 | 182 | 175 | 143 | 237 | 165 | 222 | 183 | 256 | 221 | 196 |
| Honduras | Olancho | HDROlan7 | 234 | 225 | 188 | 191 | 176 | 184 | 143 | 216 | 165 | 189 | 213 |  | 302 | 196 |
| Honduras | Olancho | HDROlan8 | 249 | 181 | 188 | 200 | 179 | 175 | 164 | 204 | 165 | 237 | 213 | 253 | 284 | 217 |
| Honduras | Olancho | HDROlan9 | 237 | 181 | 185 | 200 | 179 | 250 | 143 | 276 | 177 | 183 | 183 | 256 | 305 | 217 |
| Honduras | Olancho | HDROlan10 | 237 | 181 | 185 | 200 | 179 | 250 | 143 | 276 | 171 | 183 | 183 | 256 | 287 | 217 |
| Honduras | Olancho | HDROlan11 | 222 | 197 | 188 | 197 | 182 | 178 | 146 | 237 | 165 | 240 | 183 | 253 | 221 | 196 |
| India | NA | IND1 | 231 | 217 | 194 |  | 176 | 241 | 149 | 285 | 162 | 186 | 207 | 253 | 239 | 175 |
| India | NA | IND2 | 237 | 213 | 194 | 197 | 176 | 241 | 146 | 243 | 168 | 186 | 231 | 256 | 284 | 178 |
| India | NA | IND3 | 234 | 209 | 200 | 200 | 173 | 238 | 143 | 246 | 162 | 192 | 219 | 250 | 341 | 217 |
| India | NA | IND4 | 231 | 213 | 191 |  | 173 | 241 | 143 | 252 | 156 | 237 | 222 | 250 | 233 | 178 |
| Iran | Chabahar district | IRAN1 | 234 | 213 | 194 | 200 | 173 | 184 | 143 | 237 | 168 | 207 | 210 | 250 | 239 | 181 |
| Iran | Chabahar district | IRAN2 | 228 | 217 | 191 | 221 | 173 | 184 | 137 | 231 | 171 | 183 | 213 | 271 |  | 208 |
| Iran | Chabahar district | IRAN3 | 237 | 217 | 188 | 200 | 173 | 184 | 146 | 258 | 165 |  | 225 |  | 260 | 175 |
| Iran | Chabahar district | IRAN4 | 234 | 217 | 191 | 197 | 173 | 184 | 143 | 228 | 159 | 204 | 228 | 250 | 236 | 211 |
| Iran | Chabahar district | IRAN5 | 228 | 209 | 200 | 203 | 185 | 184 | 149 | 264 | 162 | 198 | 207 |  | 323 | 205 |
| Iran | Chabahar district | IRAN6 | 234 | 213 | 194 | 200 | 173 | 184 | 143 | 237 | 168 | 207 | 210 | 250 | 239 | 178 |
| Iran | Chabahar district | IRAN7 | 234 | 209 | 194 | 200 | 179 | 184 | 143 | 270 | 174 | 186 | 216 | 250 | 248 | 178 |
| Iran | Chabahar district | IRAN8 | 234 | 213 | 185 | 200 | 173 | 184 | 143 | 288 | 156 | 228 | 222 | 265 | 302 | 205 |
| Iran | Chabahar district | IRAN9 | 237 | 205 | 200 | 203 | 176 | 184 | 143 | 219 | 156 | 216 | 222 | 247 |  | 169 |
| Iran | Chabahar district | IRAN10 | 228 | 201 | 191 | 194 | 173 | 184 | 143 | 231 | 165 | 186 | 231 | 250 | 230 | 175 |
| Iran | Chabahar district | IRAN11 | 234 | 209 | 197 | 200 | 176 | 184 | 143 | 237 | 171 | 195 | 234 | 253 | 338 | 205 |
| Iran | Chabahar district | IRAN12 | 231 | 213 | 197 | 194 | 182 | 244 | 146 | 258 | 168 | 201 | 225 | 253 | 272 | 205 |
| Iran | Chabahar district | IRAN13 | 234 | 209 | 185 | 194 | 173 | 238 | 137 | 258 | 165 | 189 | 210 | 250 | 257 | 178 |
| Iran | Pishin district | IRAN14 | 234 | 193 | 191 | 194 | 173 | 184 | 143 | 234 | 162 | 225 | 219 | 253 | 251 | 208 |
| Iran | Pishin district | IRAN15 | 231 | 209 | 191 | 200 | 173 | 241 | 143 | 228 | 156 | 204 | 222 | 253 | 242 | 184 |
| Iran | Pishin district | IRAN16 | 234 | 245 | 188 | 194 | 173 | 238 | 143 | 312 | 156 | 201 | 210 | 250 | 212 | 208 |
| Iran | Pishin district | IRAN17 | 231 | 213 | 188 | 203 | 173 | 253 | 143 | 240 | 165 | 189 | 240 | 250 | 233 | 178 |
| Thailand/myanmar | Border Thailand/myanmar | LAOPID1 | 234 | 213 | 194 | 200 | 173 | 250 | 143 | 234 | 156 | 180 | 210 | 250 | 215 | 241 |
| Thailand/myanmar | Border Thailand/myanmar | LAOPID2 | 234 | 221 | 185 | 200 | 176 | 256 | 155 | 276 | 171 | 216 | 183 | 250 | 263 | 205 |
| Thailand/myanmar | Border Thailand/myanmar | LAOPID3 | 231 | 241 | 191 | 206 | 173 | 238 | 143 | 228 | 159 | 216 | 213 | 244 | 272 | 202 |
| Thailand/myanmar | Border Thailand/myanmar | LAOPID4 | 231 | 217 | 191 | 221 | 173 | 253 | 137 | 270 | 156 | 183 | 213 | 271 | 284 | 241 |
| Thailand/myanmar | Border Thailand/myanmar | LAOPID5 | 231 | 241 | 185 | 194 | 173 | 247 | 143 |  | 159 | 186 | 234 | 253 | 206 | 205 |
| Thailand/myanmar | Border Thailand/myanmar | LAOTHv1 | 231 | 217 | 197 | 200 | 173 | 250 | 179 | 279 | 162 | 204 | 216 | 253 |  | 199 |
| Thailand/myanmar | Border Thailand/myanmar | LAOPID6 | 231 | 213 | 191 | 212 | 173 | 259 | 146 | 261 | 159 | 195 | 210 | 250 | 311 | 190 |
| Thailand/myanmar | Border Thailand/myanmar | LAOTHv2 | 234 | 217 | 191 | 197 | 173 | 259 | 143 | 261 | 171 | 189 | 228 | 250 | 287 | 214 |
| Thailand/myanmar | Border Thailand/myanmar | LAOPID7 | 234 | 233 | 188 | 197 | 173 | 259 | 149 | 258 | 171 | 189 | 216 | 241 | 284 | 214 |
| Thailand/myanmar | Border Thailand/myanmar | LAOTHv3 | 231 | 193 | 194 | 194 | 212 | 232 | 152 | 201 | 180 | 201 | 207 | 244 | 263 | 211 |
| Thailand/myanmar | Border Thailand/myanmar | LAOPID8 | 237 | 209 | 188 | 203 | 182 | 259 | 143 | 312 | 174 | 192 | 210 | 262 | 338? | 205 |
| Thailand/myanmar | Border Thailand/myanmar | LAOTHv4 | 231 | 189 | 191 | 224 | 194 | 247 | 146 | 273 | 162 | 204 | 210 | 241 | 260 | 229 |
| Thailand/myanmar | Border Thailand/myanmar | LAOPID9 | 231 | 189 | 203 | 197 | 179 | 259 | 146 | 234 | 159 | 186 | 213 | 247 |  | 202 |
| Thailand/myanmar | Border Thailand/myanmar | LAOTHv5 | 234 | 197 | 191 | 224 | 182 | 247 | 149 | 246 | 159 | 195 | 216 | 241 | 287 | 220 |
| Thailand/myanmar | Border Thailand/myanmar | LAOPID10 | 237 | 241 | 197 | 200 | 179 | 247 | 146 | 216 | 162 | 168 | 213 | 244 | 206 | 199 |
| Thailand/myanmar | Border Thailand/myanmar | LAOTHv6 | 237 | 257 | 185 | 224 | 194 | 256 | 155 | 285 | 168 | 219 | 183 | 250 | 269 | 202 |
| Thailand/myanmar | Border Thailand/myanmar | LAOPID11 | 237 | 209 | 188 | 200 | 173 | 247 | 170 | 246 | 162 | 201 | 213 | 250 | 230 | 205 |
| Thailand/myanmar | Border Thailand/myanmar | LAOPID12 | 234 | 213 | 194 | 206 | 191 | 211 | 152 | 288 | 159 | 204 | 210 | 244 |  | 208 |
| Thailand/myanmar | Border Thailand/myanmar | LAOTHv7 | 234 | 193 | 191 | 200 | 185 | 259 | 161 | 234 | 156 | 201 | 183 | 241 | 245 | 202 |
| Thailand/myanmar | Border Thailand/myanmar | LAOTHv8 | 234 | 217 | 191 | 224 | 206 | 259 | 143 | 252 | 159 | 216 | 210 | 241 | 287 | 211 |
| Thailand/myanmar | Border Thailand/myanmar | LAOPID13 | 234 | 217 | 191 | 224 | 206 | 259 | 143 | 252 | 159 | 216 | 210 | 241 | 287 | 211 |
| Thailand/myanmar | Border Thailand/myanmar | LAOPID14 | 234 |  | 200 | 203 | 185 | 223 | 152 | 258 | 165 | 192 | 216 | 238 | 263 | 211 |
| Mauritania | NA | MAURI27 | 234 | 201 | 188 | 212 | 182 | 226 | 146 | 240 | 162 | 174 | 210 | 250 | 248 | 181 |
| Mauritania | NA | MAURI28 | 237 | 209 | 185 | 197 | 191 | 226 | 143 | 240 | 162 |  | 234 | 250 |  | 211 |
| Mauritania | NA | MAURI29 | 231 | 181 | 188 | 200 | 173 | 226 | 146 | 204 | 168 |  | 210 | 253 | 224 | 220 |
| Mauritania | NA | MAURI30 | 234 | 181 | 188 | 200 | 182 | 247 | 143 | 204 | 162 | 204 | 228 | 250 | 242 | 226 |
| Mauritania | NA | MAURI31 | 237 | 181 | 185 | 212 | 185 | 244 | 146 | 255 | 162 | 201 | 216 | 250 | 254 | 235 |
| Mauritania | NA | MAURI32 | 234 | 181 | 188 | 197 | 173 | 226 | 143 | 237 | 162 | 174 | 234 | 250 | 248 | 178 |
| Mauritania | NA | MAURI33 | 237 | 185 | 188 | 200 | 182 | 256 | 143 |  | 168 |  | 228 | 250 | 248 | 175 |
| Mauritania | NA | MAURI1 | 237 | 201 | 188 | 200 | 179 | 181 | 134 | 228 | 162 | 174 | 234 | 250 | 248 | 226 |
| Mauritania | NA | MAURI2 | 234 | 201 | 203 | 212 | 173 | 226 | 143 | 204 | 168 |  | 222 | 250 | 248 | 211 |
| Mauritania | NA | MAURI3 | 234 | 201 | 188 | 200 | 182 | 226 | 143 | 255 | 162 |  | 234 | 250 | 248 | 226 |
| Mauritania | NA | MAURI4 | 234 | 181 | 185 | 212 | 173 | 244 | 143 | 234 | 162 | 183 | 222 | 250 | 260 | 220 |
| Mauritania | NA | MAURI5 | 234 |  | 188 | 200 | 173 | 187 | 143 | 255 | 168 | 174 | 222 |  | 248 |  |
| Mauritania | NA | MAURI6 | 234 | 201 | 188 | 212 | 173 | 187 | 143 | 195 | 168 |  | 222 | 250 | 248 | 214 |
| Mauritania | NA | MAURI7 | 234 | 201 | 203 | 212 | 173 | 184 | 143 | 195 | 168 |  | 222 | 250 | 248 | 211 |
| Mauritania | NA | MAURI8 | 234 | 201 | 185 | 200 | 182 | 226 | 143 | 243 | 174 | 174 | 234 | 250 | 248 | 226 |
| Mauritania | NA | MAURI9 | 231 | 201 | 188 | 194 | 182 | 226 | 143 | 231 | 168 | 174 | 228 | 250 | 290 | 226 |
| Mauritania | NA | MAURI10 | 231 | 201 | 188 | 194 | 182 | 226 | 143 | 204 | 168 |  | 210 | 253 | 248 | 220 |
| Mauritania | NA | MAURI11 | 234 | 181 | 188 | 197 | 185 | 184 | 143 |  | 168 | 174 | 231 | 250 | 248 | 211 |
| Mauritania | NA | MAURI12 | 234 |  | 188 | 200 | 182 | 184 |  |  | 162 | 174 | 234 | 250 | 248 | 226 |
| Mauritania | NA | MAURI13 | 234 | 181 | 188 | 200 | 185 | 184 | 143 | 204 | 168 | 174 | 228 | 250 | 290 | 226 |
| Mauritania | NA | MAURI14 | 234 | 201 | 188 |  | 173 | 184 |  | 258 | 168 | 204 | 183 | 250 | 248 | 211 |
| Mauritania | NA | MAURI15 | 194 | 201 | 203 | 212 | 173 | 205 | 143 |  | 159 | 180 | 222 | 253 | 248 | 220 |
| Mauritania | NA | MAURI16 | 237 | 185 | 191 | 197 | 182 | 187 | 143 |  | 162 | 192 | 216 | 250 |  | 175 |
| Mauritania | NA | MAURI17 | 234 | 201 | 188 | 212 | 182 | 247 | 143 | 243 | 159 | 201 | 210 | 250 | 251 | 220 |
| Mauritania | NA | MAURI18 | 234 | 201 | 188 | 200 | 173 | 226 | 143 | 204 | 168 | 165 | 222 | 250 | 248 | 211 |
| Mauritania | NA | MAURI19 | 237 | 181 | 188 | 200 | 176 | 247 | 143 | 204 | 168 | 174 | 216 | 238 | 290 | 238 |
| Mauritania | NA | MAURI20 | 231 | 201 | 188 | 212 | 164 | 244 | 143 | 201 | 159 | 198 | 210 | 238 | 248 | 211 |
| Mauritania | NA | MAURI21 | 231 | 201 | 188 | 200 | 173 | 226 | 143 | 204 | 168 | 174 | 228 | 238 | 248 | 226 |
| Mauritania | NA | MAURI22 | 234 | 201 | 188 | 200 | 182 | 244 | 143 | 243 | 162 | 165 | 228 | 250 | 290 | 226 |
| Mauritania | NA | MAURI23 | 234 | 193 | 188 | 200 | 182 | 226 | 143 | 255 | 162 | 174 | 234 | 250 | 248 | 226 |
| Mauritania | NA | MAURI24 | 234 | 201 | 188 | 200 | 182 | 226 | 143 | 255 | 159 | 174 | 234 |  | 248 | 226 |
| Mauritania | NA | MAURI25 | 234 | 181 | 185 | 197 | 173 | 184 | 143 | 237 | 159 | 177 | 216 | 250 | 260 | 178 |
| Mauritania | NA | MAURI26 | 234 | 201 | 188 | 200 | 182 | 226 | 143 | 255 | 159 | 174 | 234 | 238 | 248 | 226 |
| Mexico | Mexico | MEX1 | 234 | 217 | 188 | 200 | 182 | 208 | 143 | 243 | 162 | 204 | 225 | 256 | 302 | 178 |
| Mexico | Mexico | MEX2 | 231 | 217 | 188 | 200 | 182 | 181 | 164 | 258 | 156 | 228 | 201 | 259 | 248 | 187 |
| Mexico | Mexico | MEX3 | 234 | 217 | 188 | 200 | 182 | 208 | 161 | 258 | 162 | 204 | 183 | 259 | 260 | 181 |
| Mexico | Mexico | MEX4 | 231 | 217 | 188 | 200 | 182 | 208 | 161 | 258 | 153 | 204 | 183 | 259 | 260 | 178 |
| Mexico | Mexico | MEX5 | 234 | 221 | 188 | 200 | 182 | 208 | 161 | 258 | 162 | 204 | 183 | 259 | 260 | 184 |
| Mexico | Mexico | MEX6 | 234 | 217 | 188 | 200 | 182 | 178 | 143 | 243 | 162 | 195 | 225 | 256 | 248 | 181 |
| Mexico | Mexico | MEX7 | 234 | 217 | 188 | 200 | 182 | 184 | 164 | 258 | 162 | 204 | 222 |  | 260 | 181 |
| Mexico | Mexico | MEX8 | 234 | 217 | 188 | 200 | 182 | 181 | 143 | 231 | 162 | 195 | 225 | 256 | 248 | 181 |
| Mexico | Mexico | MEX9 | 234 | 217 | 188 | 200 | 182 | 181 | 161 | 258 | 165 | 228 | 183 | 259 | 248 | 187 |
| Mexico | Mexico | MEX10 | 234 | 217 | 188 | 200 | 182 | 208 | 143 | 243 | 162 | 204 | 225 | 256 | 248 | 178 |
| Mexico | Mexico | MEX11 | 234 | 217 | 188 | 200 | 182 | 184 | 161 | 258 | 165 | 228 | 183 | 259 | 248 | 187 |
| Mexico | Mexico | MEX12 | 234 | 209 | 194 | 200 | 182 | 181 | 143 | 258 | 159 | 228 | 183 | 256 | 284 | 184 |
| Mexico | Mexico | MEX13 | 234 | 205 | 188 | 200 | 188 | 208 | 143 |  | 165 | 237 | 213 | 259 | 290 | 178 |
| Mexico | Mexico | MEX14 | 234 | 217 | 188 | 200 | 182 | 181 | 161 | 258 | 162 | 204 | 183 | 259 | 260 | 181 |
| Mexico | Mexico | MEX15 | 234 | 217 | 188 | 200 | 182 | 181 | 143 | 243 | 162 | 204 | 225 | 256 | 248 | 181 |
| Mexico | Mexico | MEX16 | 234 | 209 | 194 | 200 | 182 | 208 | 143 | 258 | 159 | 228 | 183 | 256 | 284 | 184 |
| Mexico | Mexico | MEX17 | 234 | 217 | 188 | 200 | 182 | 208 | 161 | 258 | 165 | 231 | 183 | 259 | 248 | 187 |
| Mexico | Mexico | MEX18 | 234 | 205 | 188 | 200 | 188 | 208 | 143 |  | 165 | 237 | 213 | 259 | 290 | 178 |
| Mexico | Mexico | MEX19 | 234 | 217 | 188 | 200 | 182 | 208 | 161 | 258 | 165 | 228 | 183 | 259 | 248 | 187 |
| Mexico | Mexico | MEX20 | 234 | 217 | 188 | 200 | 182 | 208 | 143 | 243 | 153 | 204 | 225 | 256 | 248 | 181 |
| Mexico | Mexico | MEX21 | 231 | 217 | 188 | 200 | 173 | 181 | 143 | 243 | 162 | 198 | 213 | 244 | 248 | 181 |
| Mexico | Mexico | MEX22 | 234 | 217 | 179 | 200 | 173 | 208 | 161 | 258 | 162 | 204 | 183 | 247 | 260 | 178 |
| Mexico | Mexico | MEX23 | 231 | 205 | 188 | 200 | 176 | 211 | 143 |  | 165 | 237 | 204 | 247 | 290 | 178 |
| Mexico | Mexico | MEX24 | 234 | 217 | 188 | 200 | 173 | 178 | 143 | 243 | 162 | 204 | 213 | 244 | 248 | 181 |
| Mexico | Mexico | MEX25 | 231 | 221 | 188 | 200 | 182 | 235 | 143 | 225 | 165 | 216 | 183 | 256 | 260 | 181 |
| Mexico | Mexico | MEX26 | 234 | 213 | 188 | 200 | 173 | 208 | 143 | 243 | 162 | 204 | 216 | 244 | 248 | 181 |
| Mexico | Mexico | MEX27 | 234 | 217 | 188 | 200 | 182 | 184 | 161 | 258 | 165 | 231 | 183 | 259 | 248 | 187 |
| Mexico | Mexico | MEX28 | 234 | 221 | 188 | 200 | 182 | 184 | 143 | 225 | 165 | 216 | 183 | 256 | 260 | 181 |
| Mexico | Mexico | MEX29 | 234 | 217 | 188 | 200 | 182 | 184 | 146 | 258 | 162 | 204 | 225 | 256 | 248 | 181 |
| Mexico | Mexico | MEX30 | 231 | 201 | 185 | 200 | 179 | 232 | 143 | 249 | 156 | 222 | 183 | 250 | 233 | 241 |
| Pakistan | Chabahar district | PKT1 | 234 | 201 | 188 | 194 | 173 | 247 | 146 | 258 | 162 | 183 | 228 | 253 | 245 | 223 |
| Pakistan | Chabahar district | PKT2 | 237 | 213 | 197 | 200 | 176 | 247 | 137 | 234 | 168 | 183 | 210 | 253 | 272 | 172 |
| Pakistan | Chabahar district | PKT3 | 237 | 221 | 188 | 200 | 176 | 187 | 137 | 240 | 156 | 183 | 225 | 250 |  | 169 |
| Pakistan | Chabahar district | PKT4 | 234 | 213 | 185 | 200 | 173 | 244 | 143 | 249 | 162 | 183 | 222 | 253 | 230 | 175 |
| Pakistan | Chabahar district | PKT5 | 237 | 241 | 194 | 200 | 185 | 247 | 137 | 273 | 162 | 186 |  | 259 | 254 | 178 |
| Pakistan | Chabahar district | PKT6 | 237 | 241 | 194 | 200 | 185 | 250 | 137 | 273 | 162 | 186 | 210 | 259 | 254 | 181 |
| Pakistan | Chabahar district | PKT7 | 234 | 209 | 194 | 203 | 173 | 244 | 146 | 255 | 165 | 198 | 210 | 256 | 233 | 172 |
| Pakistan | Chabahar district | PKT8 | 234 | 193 |  | 200 | 173 | 184 | 143 | 225 | 156 | 201 |  | 253 | 221 | 175 |
| Pakistan | Chabahar district | PKT9 | 231 | 209 | 188 | 203 | 176 | 187 | 146 | 228 | 153 | 180 | 207 | 247 | 317 | 217 |
| Pakistan | Chabahar district | PKT10 | 234 | 213 | 194 | 206 | 179 | 247 | 137 | 276 | 156 | 186 | 213 | 253 | 272 | 214 |
| Pakistan | Chabahar district | PKT11 | 234 | 209 | 188 | 200 | 176 | 187 | 143 | 261 | 165 | 207 | 225 | 256 | 245 | 208 |
| Pakistan | Chabahar district | PKT12 | 231 | 209 | 200 | 200 | 179 | 184 | 143 | 276 | 159 | 183 | 225 | 247 | 308 | 223 |
| Pakistan | Chabahar district | PKT13 | 237 | 209 | 203 | 218 | 176 | 187 | 143 | 261 | 159 | 204 | 225 | 256 | 245 | 208 |
| Pakistan | Chabahar district | PKT14 | 237 | 209 | 203 | 218 | 176 | 187 | 143 | 261 | 159 | 207 | 225 | 256 | 245 | 208 |
| Pakistan | Chabahar district | PKT15 | 234 | 213 | 200 | 203 | 173 | 244 | 137 | 252 | 168 | 183 | 222 | 277 | 263 | 199 |
| Pakistan | Chabahar district | PKT16 | 234 | 213 | 200 | 203 | 173 | 244 | 137 | 252 | 168 | 183 | 222 | 277 | 263 | 199 |
| Pakistan | Chabahar district | PKT17 | 237 | 209 | 203 | 218 | 176 | 187 | 143 | 261 | 165 | 207 | 225 | 256 | 245 | 208 |
| Pakistan | Chabahar district | PKT18 | 234 | 221 | 194 | 194 | 173 | 244 | 143 | 249 | 156 | 210 | 210 | 250 | 341 | 172 |
| Pakistan | Chabahar district | PKT19 | 234 | 209 | 188 | 203 | 176 | 184 | 143 | 225 | 150 | 189 | 225 | 247 | 302 | 196 |
| Pakistan | Chabahar district | PKT20 | 237 | 209 | 188 | 194 | 173 | 187 | 143 | 225 | 162 | 183 | 222 | 250 | 233 | 175 |
| Pakistan | Chabahar district | PKT21 | 234 | 209 | 194 | 197 | 173 | 244 | 137 | 255 | 162 | 189 | 222 | 247 | 254 | 175 |
| Pakistan | Chabahar district | PKT22 | 243 | 209 | 194 | 212 | 173 | 241 | 143 | 282 | 168 | 183 | 219 | 241 | 251 | 178 |
| Pakistan | Chabahar district | PKT23 | 234 | 209 | 194 | 206 | 167 | 238 | 143 |  | 177 | 198 | 228 | 250 | 296 | 214 |
| Pakistan | Chabahar district | PKT24 | 228 | 213 | 188 | 212 | 176 | 256 | 137 | 240 | 156 | 186 | 222 | 241 | 287 | 187 |
| Pakistan | Chabahar district | PKT25 | 234 | 209 | 194 | 206 | 167 | 238 | 143 |  | 177 | 198 | 228 | 250 | 296 | 214 |
| Pakistan | Chabahar district | PKT26 | 228 | 213 | 188 | 212 | 176 | 256 | 137 | 240 | 156 | 186 | 222 | 241 | 287 | 187 |
| Pakistan | Chabahar district | PKT27 | 237 | 209 | 191 | 206 | 173 | 184 | 146 | 243 | 168 | 198 | 225 | 253 | 287 | 169 |
| Peru | Azucenas SJ/Maynas | PERU1 | 237 | 209 | 191 | 197 | 179 | 184 | 146 | 240 | 159 | 213 | 210 | 256 | 290 | 208 |
| Peru | Santa Clara SJ/Maynas | PERU2 | 234 | 209 | 188 | 203 | 173 | 235 | 143 | 255 | 159 | 213 | 225 | 244 |  | 220 |
| Peru | Rumococha SJ/Maynas | PERU3 | 240 | 221 | 188 | 197 | 179 | 208 | 143 | 294 | 162 | 213 | 228 | 250 | 197 | 220 |
| Peru | Camelias SJ/Maynas | PERU4 | 240 | 205 | 188 | 200 | 173 | 241 | 143 | 273 | 162 | 210 | 237 | 250 |  | 220 |
| Peru | Maynas-San Juan | PERU5 | 234 | 209 | 194 | 194 |  | 229 | 143 | 255 | 171 | 192 | 228 |  | 248 | 196 |
| Peru | Maynas-San Juan | PERU6 | 234 | 209 | 188 | 200 | 179 | 238 | 143 | 261 | 171 | 192 | 210 | 256 | 275 | 211 |
| Peru | Maynas-San Juan | PERU7 | 234 | 205 | 191 | 203 | 179 | 229 | 143 | 255 | 159 | 213 | 249 | 244 | 170 | 211 |
| Peru | Maynas-Iquitos | PERU8 | 240 | 209 | 188 | 203 | 173 | 226 | 143 | 261 | 159 | 192 | 210 | 244 |  | 202 |
| Peru | Maynas-Iquitos | PERU9 | 237 | 201 | 188 | 191 | 173 | 250 | 143 | 255 | 165 | 213 | 210 | 256 | 299 | 208 |
| Peru | Maynas-Iquitos | PERU10 | 234 | 189 | 194 | 197 | 173 | 238 | 143 | 273 | 159 | 204 | 249 | 223 | 293 | 205 |
| Peru | Napo river | PERU11 | 234 | 205 | 188 | 197 | 179 | 244 | 152 | 255 | 147 | 213 | 228 | 256 |  | 211 |
| Peru | Maynas-Iquitos | PERU12 | 234 | 189 | 194 | 197 | 173 | 241 | 143 | 261 | 165 |  | 210 | 250 | 293 | 202 |
| Peru | Maynas-Iquitos | PERU13 | 231 | 209 | 197 | 200 | 179 | 208 | 149 | 264 | 171 | 213 | 225 | 247 | 299 | 205 |
| Peru | Caballacocha-San Pablo | PERU14 | 234 | 193 | 188 | 203 | 176 | 184 | 143 | 255 | 171 | 213 |  | 250 |  | 205 |
| Peru | Azucenas SJ/Maynas | PERU15 | 234 | 221 | 191 | 197 | 179 | 187 | 143 | 258 | 165 | 213 | 228 | 244 | 197 | 220 |
| Peru | Azucenas SJ/Maynas | PERU16 | 234 | 193 | 191 | 212 | 173 | 184 | 143 | 258 | 180 | 195 | 225 | 241 | 239 | 199 |
| Peru | 25 de Enero SJ/Maynas | PERU17 | 240 | 205 | 188 | 200 | 173 | 238 | 143 | 261 | 165 | 213 | 234 | 250 |  | 220 |
| Peru | Maynas-San Juan | PERU18 | 234 | 205 | 191 | 194 | 173 | 208 | 143 | 255 | 165 | 213 | 225 | 259 | 263 | 208 |
| Peru | Maynas-Iquitos | PERU19 | 234 | 209 | 188 | 197 | 179 | 229 | 143 |  | 174 | 231 | 210 | 247 | 239 | 220 |
| Peru | Maynas-Iquitos | PERU20 |  | 189 | 188 | 203 | 173 | 229 | 143 | 267 | 147 | 213 | 249 | 259 | 233 | 211 |
| Peru | Maynas-San Juan | PERU21 | 234 | 205 | 191 |  | 173 | 208 | 143 | 255 | 165 | 213 | 225 | 259 |  | 208 |
| Peru | Maynas-Iquitos | PERU22 | 234 | 209 |  | 197 | 179 | 211 | 143 |  |  | 213 | 234 |  | 251 | 199 |
| Peru | Maynas-Iquitos | PERU23 | 234 | 209 | 188 | 203 | 173 | 238 | 143 | 261 | 171 | 213 | 225 | 244 |  | 211 |
| Peru | Maynas-San Juan | PERU24 | 240 | 193 | 191 | 194 | 179 | 241 | 143 | 273 | 162 | 213 | 222 | 250 | 251 | 208 |
| Peru | Maynas-San Juan | PERU25 | 231 | 205 | 188 | 200 | 179 | 229 | 143 | 255 | 171 | 225 | 210 | 250 | 209 | 217 |
| Peru | Maynas-Iquitos | PERU26 | 231 | 189 | 185 |  | 188 | 229 | 146 |  |  | 225 |  |  | 197 | 217 |
| Peru | Maynas-Iquitos | PERU27 | 234 | 197 | 191 | 200 | 179 | 229 | 143 | 258 | 168 | 213 | 249 | 256 | 287 | 217 |
| Peru | Maynas-San Juan | PERU28 | 234 | 205 | 200 | 197 | 179 | 238 | 143 | 255 | 162 | 213 | 225 | 259 | 197 | 211 |
| Peru | Maynas-Iquitos | PERU29 | 234 | 189 | 197 | 188 | 179 | 238 | 143 | 255 | 171 | 213 | 258 | 259 | 281 | 205 |
| Peru | Curaray-Napo River | PERU30 | 234 | 209 | 188 |  | 173 | 241 | 143 | 258 | 165 | 213 | 225 | 244 |  | 220 |
| Peru | Maynas-Iquitos | PERU31 | 237 | 201 | 188 | 197 | 173 | 229 | 146 | 255 | 162 | 213 | 225 | 256 | 290 | 208 |
| Peru | Maynas-San Juan | PERU32 | 234 | 189 | 197 | 197 | 179 | 232 | 143 |  | 168 | 204 | 225 |  | 209 | 220 |
| Peru | Maynas-San Juan | PERU33 | 237 | 201 | 188 | 200 | 179 |  | 143 | 255 | 165 | 213 | 225 | 256 |  | 244 |
| Peru | Maynas-Iquitos | PERU34 |  | 201 | 188 |  | 179 | 244 | 143 |  |  | 213 | 222 | 256 | 251 |  |
| Peru | Iquitos-Rumococha | PERU35 | 234 | 189 | 188 | 197 | 179 | 229 | 143 |  | 168 | 231 | 225 | 259 | 209 | 220 |
| Peru | Iquitos-Las Palmeras Terminal | PERU36 | 237 | 205 | 188 | 206 | 179 | 241 | 143 | 273 | 147 | 231 | 243 | 256 | 221 | 199 |
| Peru | Maynas-San Juan | PERU37 | 234 | 201 | 188 | 197 | 179 | 232 | 143 | 261 | 162 | 192 | 234 | 247 | 185 | 199 |
| Peru | Maynas-Los Delfines | PERU38 | 237 | 209 | 188 |  | 179 | 232 | 143 | 261 | 165 | 231 | 180 | 235 | 275 | 199 |
| Peru | Maynas-San Juan | PERU39 | 234 | 189 | 191 | 197 | 173 |  | 146 | 261 | 165 |  |  | 235 | 299 | 199 |
| Peru | Maynas-San Juan | PERU40 | 234 | 189 | 188 | 197 | 179 | 238 | 143 |  | 168 | 216 | 225 | 250 | 275 | 211 |
| Peru | Maynas-San Juan | PERU41 | 234 | 205 | 194 | 206 | 179 | 211 | 143 | 255 | 165 | 228 | 252 | 250 | 290 | 220 |
| Peru | Alto Nanay | PERU42 | 237 | 209 | 191 | 197 | 179 | 232 | 143 | 249 | 147 | 213 | 252 | 259 | 227 | 199 |
| Peru | Maynas-San Juan | PERU43 | 234 | 197 | 188 | 200 | 173 | 238 | 143 | 300 | 171 | 237 | 243 | 250 |  | 202 |
| Peru | Maynas-San Juan | PERU44 | 234 | 209 | 188 | 212 | 179 | 229 | 143 | 210 | 159 | 192 | 231 | 235 | 197 | 208 |
| Peru | Maynas-Iquitos | PERU45 | 243 | 209 | 188 | 197 | 182 | 229 | 143 | 255 | 165 | 213 |  | 250 | 251 | 205 |
| Peru | Maynas-Iquitos | PERU46 | 234 | 201 | 194 | 200 | 179 | 229 | 143 | 273 | 168 | 237 | 258 | 259 | 251 | 220 |
| Peru | Maynas-Iquitos | PERU47 | 234 | 189 | 194 | 197 | 173 | 241 | 143 | 261 | 171 | 213 | 210 | 250 | 290 | 208 |
| Peru | Maynas-Amazon river | PERU48 | 234 | 193 | 188 | 194 | 179 | 244 | 143 | 273 | 177 | 213 | 234 | 250 | 239 | 208 |
| Peru | Camelias SJ/Maynas | PERU49 | 240 | 205 | 188 | 200 | 173 | 244 | 143 | 273 | 165 | 210 | 234 | 250 |  | 220 |
| Peru | Monasi SJ/Maynas | PERU50 | 240 | 205 | 188 | 200 | 173 | 241 | 143 | 273 | 165 | 210 | 234 | 250 | 278 | 220 |
| Peru | Camelias SJ/Maynas | PERU51 | 240 | 209 | 188 | 200 | 173 | 241 | 143 | 261 | 171 | 213 | 225 | 250 | 209 | 235 |
| Peru | Simon Bolivar SJ/Maynas | PERU52 | 240 | 189 | 191 | 197 | 179 | 226 | 143 | 273 | 159 | 231 | 228 | 250 |  | 199 |
| Peru | Laguna Azul SJ/Maynas | PERU53 | 240 | 205 | 188 | 200 | 173 | 229 | 143 | 258 | 165 | 213 | 234 | 235 |  | 220 |
| Peru | Punchana/Maynas | PERU54 | 234 | 221 | 188 | 197 | 179 | 211 | 143 | 261 | 165 | 213 | 228 | 244 |  | 211 |
| Peru | Quistococha SJ/Maynas | PERU55 | 234 | 189 | 191 | 194 | 173 | 244 | 143 | 258 | 180 | 213 | 258 | 253 |  | 220 |
| Peru | Quistococha SJ/Maynas | PERU56 | 240 | 197 | 188 | 200 | 173 | 211 | 146 | 294 | 147 | 210 | 210 | 250 | 338 | 220 |
| Peru | Pucayacu SJ/Maynas | PERU57 | 240 | 205 | 188 | 200 | 173 | 244 | 143 | 252 | 165 | 210 | 234 | 250 | 308 | 220 |
| Peru | Simon Bolivar SJ/Maynas | PERU58 | 237 | 189 | 188 | 197 | 179 | 241 | 149 | 261 | 162 | 213 | 258 | 250 | 236 | 205 |
| Peru | America SJ/Maynas | PERU59 | 234 | 189 | 191 | 194 | 179 | 229 | 143 | 255 | 165 | 192 | 210 | 268 | 209 | 223 |
| Peru | Shiriara SJ/Maynas | PERU60 | 240 | 205 | 188 | 200 | 173 | 241 | 143 | 273 | 165 | 210 | 234 | 250 |  | 220 |
| Peru | Camelias SJ/Maynas | PERU61 | 234 | 209 | 188 | 203 | 173 | 241 | 143 | 261 | 171 | 213 | 225 | 244 |  | 211 |
| Peru | Santa Clara SJ/Maynas | PERU62 | 234 | 221 | 191 | 197 | 179 | 208 | 143 | 258 | 165 | 213 | 228 | 244 | 197 | 220 |
| Peru | Santa Clara SJ/Maynas | PERU63 | 240 | 189 | 194 | 212 | 179 | 238 | 143 | 258 | 159 | 234 | 210 | 241 |  | 211 |
| Peru | San Juan/Maynas | PERU64 | 234 | 193 | 191 | 212 | 173 | 229 | 143 | 258 | 180 | 195 | 225 | 241 | 239 | 199 |
| Peru | Bella Luz SJ/Maynas | PERU65 | 234 | 225 | 191 | 197 | 179 | 208 | 143 | 258 | 165 | 213 | 228 | 244 | 197 | 220 |
| Peru | Santa Clara SJ/Maynas | PERU66 | 234 | 209 | 194 | 203 | 173 | 229 | 143 | 285 | 147 | 219 | 252 | 259 | 209 | 205 |
| Peru | Santa Clara SJ/Maynas | PERU67 | 234 | 209 | 191 | 197 | 179 | 241 | 146 | 261 | 147 | 213 | 210 | 268 | 287 | 208 |
| Peru | Santa Clara SJ/Maynas | PERU68 | 234 | 189 | 188 | 194 | 173 | 241 | 143 | 240 | 171 | 213 | 234 | 250 |  | 205 |
| Peru | Santa Clara SJ/Maynas | PERU69 | 234 | 209 | 191 | 197 | 179 | 241 | 146 | 261 | 147 | 213 | 210 | 268 | 287 | 208 |
| Sudan | New Halfa | SUDHL1 | 234 | 237 | 194 | 206 | 173 | 226 | 143 | 249 | 153 | 219 | 222 | 262 | 221 | 217 |
| Sudan | Khartoum | SUDKH1 | 234 | 217 | 191 | 206 | 170 | 253 | 149 | 249 | 159 | 189 | 210 | 247 | 341 | 169 |
| Sudan | New Halfa | SUDHL2 | 240 | 229 | 194 | 206 | 176 |  | 146 | 243 | 159 | 186 | 228 |  | 230 | 175 |
| Sudan | Khartoum | SUDKH2 | 234 | 237 | 191 | 215 | 182 | 244 | 149 | 270 | 156 | 183 | 219 | 256 | 299 | 211 |
| Sudan | Khartoum | SUDKH3 | 237 | 229 | 194 | 203 | 179 | 259 | 143 | 243 | 159 | 210 | 228 | 250 | 302 | 184 |
| Sudan | Khartoum | SUDKH4 | 234 | 213 | 200 | 200 | 182 | 244 | 143 |  | 162 | 210 | 222 | 247 | 230 | 154 |
| Sudan | Khartoum | SUDKH5 | 231 | 229 | 188 | 200 | 179 | 229 | 143 | 252 | 171 | 186 | 210 | 247 | 230 | 178 |
| Sudan | Khartoum | SUDKH6 | 240 | 229 | 191 |  | 179 | 247 | 146 | 204 | 147 | 186 | 219 | 247 | 239 | 175 |
| Sudan | New Halfa | SUDHL3 | 234 | 209 | 188 | 206 | 191 | 241 | 143 |  | 180 | 183 | 210 | 250 | 230 | 214 |
| Sudan | Khartoum | SUDKH7 | 231 | 181 | 185 | 197 | 179 | 241 | 143 | 210 | 162 | 183 | 228 | 277 | 266 | 169 |
| Sudan | New Halfa | SUDHL4 | 237 | 229 | 188 | 221 | 173 | 241 | 137 | 225 | 162 | 222 | 228 | 262 | 314 | 226 |
| Sudan | Khartoum | SUDKH8 | 234 | 209 | 191 | 200 | 170 | 250 | 143 | 291 | 153 | 210 | 222 | 247 | 272 | 178 |
| Sudan | Khartoum | SUDKH9 | 231 | 229 | 188 | 200 | 179 | 229 | 146 | 252 | 171 | 186 | 210 | 247 | 230 | 178 |
| Sudan | Khartoum | SUDKH10 | 237 | 249 | 194 | 200 | 170 | 241 | 146 |  | 159 | 210 | 228 | 250 | 302 | 184 |
| Sudan | New Halfa | SUDHL5 | 231 |  | 194 | 227 | 182 | 250 | 137 |  | 153 | 186 | 222 |  | 239 | 169 |
| Sudan | New Halfa | SUDHL6 |  | 181 | 185 |  | 176 |  | 146 | 288 | 147 |  | 210 | 244 | 251 | 238 |
| Sudan | New Halfa | SUDHL7 | 237 | 205 | 197 |  | 182 | 247 | 143 | 279 | 147 | 183 | 231 |  | 269 | 229 |
| Sudan | Khartoum | SUDKH11 | 231 | 217 | 194 | 221 | 179 | 241 | 143 | 291 | 159 | 186 | 210 | 247 | 230 | 220 |
| Sudan | Khartoum | SUDKH12 | 228 | 205 | 194 |  | 185 | 241 | 143 |  | 153 | 186 | 210 | 250 | 221 | 196 |
| Sudan | Khartoum | SUDKH13 | 234 | 209 | 188 | 200 | 185 | 241 | 143 | 204 | 168 | 210 | 210 | 247 | 251 | 220 |
| Sudan | Khartoum | SUDKH14 | 240 | 233 | 191 | 224 | 185 | 247 | 143 | 204 | 156 | 210 | 222 | 277 | 239 | 169 |
| Sudan | Khartoum | SUDKH15 | 237 | 181 | 188 | 206 | 176 | 241 | 143 | 207 | 162 | 183 | 228 | 247 |  | 175 |
| Sudan | Khartoum | SUDKH16 | 234 | 217 | 191 | 206 | 170 | 253 | 149 | 249 | 159 | 186 | 210 | 247 | 341 | 169 |
| Sudan | Khartoum | SUDKH17 | 234 | 209 | 191 | 206 | 170 | 250 | 149 | 252 | 162 | 189 | 210 | 247 | 341 | 217 |
| Sudan | Khartoum | SUDKH18 | 228 | 213 | 185 |  | 185 | 229 | 143 | 252 | 168 | 183 | 228 | 250 | 239 | 178 |
| Sudan | New Halfa | SUDHL8 | 237 | 225 | 188 | 227 | 173 | 253 | 149 | 207 | 153 | 183 | 222 | 262 | 245 | 211 |
| Sudan | Khartoum | SUDKH19 | 228 | 205 | 194 |  | 185 | 241 | 143 | 201 | 153 | 189 | 210 | 250 | 221 | 196 |
| Sudan | Khartoum | SUDKH20 | 240 | 233 | 191 | 224 | 185 | 247 | 140 | 204 | 156 | 210 | 222 | 277 | 239 | 169 |
| Sudan | Khartoum | SUDKH21 | 231 | 213 | 188 | 197 | 179 | 250 | 143 |  | 168 | 171 | 228 | 256 | 221 | 205 |
| Sudan | Khartoum | SUDKH22 | 231 | 217 | 191 | 206 | 170 | 253 | 149 | 249 | 159 | 186 | 210 | 247 | 272 | 169 |
| Sudan | Khartoum | SUDKH23 | 234 | 217 | 191 | 206 | 170 | 253 | 149 | 249 | 159 | 189 | 210 | 247 | 341 | 169 |
| Sudan | Khartoum | SUDKH24 | 234 | 217 | 191 | 206 | 170 | 250 | 149 | 252 | 159 | 189 | 210 | 247 | 341 | 169 |
| Sudan | Khartoum | SUDKH25 | 240 | 233 | 191 | 224 | 185 | 247 | 140 | 204 | 156 | 210 | 222 | 277 | 239 | 169 |
| Sudan | Khartoum | SUDKH26 | 237 | 181 | 191 | 206 | 176 | 226 | 143 | 294 | 147 | 210 | 222 | 253 | 311 | 181 |
| Sudan | Khartoum | SUDKH27 | 234 | 209 | 191 | 206 | 170 | 253 | 149 | 249 | 159 | 189 | 210 | 277 | 272 | 217 |
| Sudan | Khartoum | SUDKH28 | 228 | 205 | 194 |  | 185 | 241 | 143 | 207 | 153 | 186 | 210 | 250 | 221 | 196 |
| Sudan | Khartoum | SUDKH29 | 231 | 217 | 194 | 197 | 179 | 241 | 143 | 291 | 159 | 183 | 210 | 247 | 230 | 220 |
| Sudan | Khartoum | SUDKH30 | 234 | 237 | 191 | 215 | 182 | 244 | 149 | 270 | 156 | 183 | 219 | 256 | 299 | 211 |
| Sudan | Khartoum | SUDKH31 | 237 | 213 | 194 | 197 | 179 | 244 | 143 | 249 | 159 | 186 | 210 | 262 |  | 217 |
| Sudan | Khartoum | SUDKH32 | 234 | 217 | 191 | 206 | 170 | 250 | 149 | 249 | 159 | 186 | 210 | 247 | 341 | 169 |
| Sudan | Khartoum | SUDKH33 | 234 | 217 | 191 | 206 | 170 | 250 | 149 | 249 | 159 | 186 | 210 | 247 | 341 | 169 |
| Sudan | Khartoum | SUDKH34 | 228 | 269 |  | 197 | 185 | 244 |  |  | 171 | 210 | 228 | 247 | 311 | 238 |
| Sudan | Khartoum | SUDKH35 | 246 | 253 | 191 | 227 | 176 | 247 | 146 | 252 | 159 | 210 | 219 | 247 | 230 | 184 |
| Sudan | Khartoum | SUDKH36 | 237 | 181 | 188 | 206 | 179 | 241 | 146 | 207 | 171 | 186 | 228 | 244 | 209 | 238 |
| Sudan | New Halfa | SUDHL9 | 231 | 213 | 185 |  | 173 | 250 | 140 | 213 | 162 | 183 | 225 | 244 | 230 | 220 |
| Sudan | Khartoum | SUDKH37 | 237 | 181 | 194 | 227 | 176 | 244 | 143 | 207 | 159 | 186 | 228 | 277 | 311 | 184 |
| Sudan | Khartoum | SUDKH38 | 234 | 217 | 191 | 206 | 170 | 250 | 149 | 249 | 159 | 189 | 210 | 247 | 341 | 169 |
| Sudan | New Halfa | SUDHL10 | 234 | 209 | 191 | 206 | 173 | 250 | 143 | 207 | 159 | 207 | 225 | 271 | 341 | 205 |
| Sudan | New Halfa | SUDHL11 | 234 | 213 | 185 | 206 | 188 | 244 | 143 | 204 | 159 | 183 | 213 | 262 | 275 | 214 |
| Sudan | New Halfa | SUDHL12 | 234 | 217 | 188 |  | 182 | 250 |  |  |  | 207 | 222 | 250 |  | 205 |
| Sudan | Khartoum | SUDKH39 | 234 | 217 | 191 | 206 | 170 | 250 | 149 | 249 | 159 | 186 | 210 | 247 | 341 | 169 |
| Sudan | Khartoum | SUDKH40 | 234 | 229 | 188 | 206 | 173 | 247 | 149 | 204 | 168 | 210 | 234 | 244 | 326 | 211 |
| Sudan | Khartoum | SUDKH41 | 234 | 221 | 194 | 203 | 182 | 241 | 134 | 237 | 153 | 186 | 228 | 250 | 248 | 196 |
| Sudan | New Halfa | SUDHL11 | 237 | 241 | 188 | 206 | 179 | 250 | 140 | 237 | 153 | 186 | 231 | 262 | 341 | 172 |
| Sudan | Khartoum | SUDKH42 | 234 | 181 | 191 | 206 | 176 | 229 | 143 | 294 | 147 | 210 | 222 | 253 | 311 | 181 |
| Sudan | Khartoum | SUDKH43 | 240 |  |  | 200 | 179 |  | 143 | 237 | 159 | 189 | 210 | 250 | 251 | 175 |
| Sudan | Khartoum | SUDKH44 | 231 | 217 | 194 | 194 | 179 | 241 | 143 | 291 | 159 | 183 | 210 | 247 | 230 | 220 |
| Sudan | New Halfa | SUDHL12 | 237 | 217 | 191 | 206 | 170 | 241 | 149 |  | 153 | 219 | 210 | 247 | 221 | 196 |
| Sudan | Khartoum | SUDKH45 | 228 | 205 | 194 | 197 | 185 | 241 | 143 |  | 153 | 189 | 210 | 250 | 221 | 196 |
| Sudan | Khartoum | SUDKH46 | 234 | 197 | 194 | 194 | 179 | 241 | 140 |  | 156 | 183 | 228 | 247 | 230 | 154 |
| Sudan | Khartoum | SUDKH47 | 231 | 217 | 194 | 194 | 179 | 241 | 143 | 291 | 159 | 186 | 210 | 247 | 230 | 220 |
| Sudan | New Halfa | SUDHL13 | 231 | 213 | 197 | 236 | 182 | 256 | 137 |  | 153 | 183 | 210 | 256 | 272 | 190 |
| Sudan | Khartoum | SUDKH48 | 234 | 217 | 191 | 206 | 170 | 250 | 149 | 249 | 159 | 189 | 210 | 247 | 341 | 169 |
| Sudan | Khartoum | SUDKH49 | 234 | 217 | 191 | 206 | 170 | 253 | 149 | 249 | 159 | 189 | 210 | 247 | 341 | 169 |
| Sudan | New Halfa | SUDHL14 | 240 | 221 | 191 | 194 | 176 | 244 | 140 | 204 | 156 | 180 |  | 247 | 251 | 178 |
| Sudan | Khartoum | SUDKH50 | 231 | 229 | 188 | 200 | 173 | 244 | 143 |  | 153 | 210 | 216 | 247 | 260 | 154 |
| Sudan | Khartoum | SUDKH51 | 240 | 233 | 191 | 224 | 185 | 247 | 140 | 204 | 156 | 210 | 222 | 277 | 239 | 169 |
| Sudan | Khartoum | SUDKH52 | 237 | 213 | 194 | 200 | 182 | 250 | 143 | 213 | 180 | 186 | 219 | 247 | 209 | 214 |
| Sudan | Khartoum | SUDKH53 | 234 | 209 | 185 | 209 | 170 | 241 | 149 | 225 | 162 | 183 | 228 | 247 | 341 | 169 |
| Sudan | Khartoum | SUDKH54 | 234 | 217 | 191 | 206 | 170 | 253 | 149 | 249 | 159 | 186 | 210 | 247 | 341 | 169 |
| Sudan | Khartoum | SUDKH55 | 240 | 233 | 191 | 224 | 185 | 247 | 140 | 204 | 156 | 210 | 222 | 283 | 239 | 169 |
| Sudan | Khartoum | SUDKH56 | 228 | 205 | 194 | 194 | 185 | 187 | 146 | 258 | 153 |  | 210 | 250 | 221 | 196 |
| Sudan | Khartoum | SUDKH57 | 231 | 229 | 188 | 191 | 185 | 244 | 143 | 213 | 171 | 186 | 210 | 247 | 239 | 178 |
| Sudan | New Halfa | SUDHL15 | 231 | 241 | 185 | 218 | 185 | 229 | 143 |  | 159 | 183 | 210 | 250 | 239 | 214 |
| Sudan | Khartoum | SUDKH58 | 234 | 209 | 191 | 206 | 170 | 253 | 149 | 249 | 162 | 186 | 210 | 247 | 341 | 217 |
| Sudan | Khartoum | SUDKH59 | 228 | 205 | 194 |  | 185 | 241 | 143 |  | 153 | 189 | 210 | 250 | 221 | 199 |
| Sudan | Khartoum | SUDKH60 | 228 | 205 | 194 |  | 185 | 241 | 143 | 258 | 153 | 186 | 210 | 250 | 221 | 196 |
| Sudan | New Halfa | SUDHL16 | 234 | 229 | 188 | 194 | 176 | 187 | 143 | 243 | 168 | 207 | 216 | 247 | 314 | 208 |
| Sudan | Khartoum | SUDKH61 | 231 | 249? | 194 | 200 | 179 | 244 | 143 | 243 | 159 | 189 | 210 | 253 | 299 | 193 |
| Sudan | Khartoum | SUDKH62 | 234 | 217 | 191 | 206 | 170 | 241 | 149 | 249 | 159 | 186 | 210 | 247 | 341 | 169 |
| Sudan | Khartoum | SUDKH63 | 234 | 217 | 191 | 206 | 170 | 187 | 149 | 249 | 159 | 186 | 210 | 247 | 341 | 169 |
| Sudan | Khartoum | SUDKH64 | 240 | 233 | 191 | 224 | 185 | 247 | 140 | 204 | 156 | 210 | 222 | 277 | 239 | 169 |
| Sudan | Khartoum | SUDKH65 | 240 | 181 | 191 | 206 | 185 | 244 | 140 |  | 156 | 183 | 210 | 253 | 239 | 178 |
| Sudan | Khartoum | SUDKH66 | 234 | 217 | 191 | 206 | 170 | 187 | 149 | 249 | 159 | 186 | 210 | 247 | 341 | 169 |
| Sudan | Khartoum | SUDKH67 | 237 | 181 | 188 | 206 | 179 | 241 | 143 | 207 | 171 | 183 | 228 | 244 | 224 | 208 |
| Sudan | New Halfa | SUDHL17 | 240 | 217 | 185 | 197 | 173 | 253 | 143 | 291 | 147 | 207 | 210 | 262 | 221 | 202 |
| Sudan | Khartoum | SUDKH68 | 240 | 233 | 191 | 224 | 185 | 247 | 140 | 204 | 156 | 210 | 222 | 277 | 239 | 169 |
| Sudan | Khartoum | SUDKH69 | 234 | 217 | 185 | 224 | 179 | 241 | 143 | 225 | 156 | 186 | 228 | 277 | 239 | 169 |
| Sudan | Khartoum | SUDKH70 | 231 | 217 | 191 | 206 | 170 | 187 | 149 | 249 | 159 | 186 | 210 | 247 | 341 | 169 |
| Sudan | Khartoum | SUDKH71 | 234 | 217 | 191 | 206 | 170 | 250 | 149 | 249 | 159 | 186 | 210 | 247 | 341 | 169 |
| Sudan | Khartoum | SUDKH72 | 237 | 201 | 194 | 194 | 176 | 241 | 143 | 207 | 153 | 180 | 213 | 253 | 251 | 232 |
| Sudan | Khartoum | SUDKH73 | 237 | 209 | 185 | 194 | 179 | 244 | 140 | 207 | 165 | 183 | 210 | 247 | 230 | 232 |
| Sudan | Khartoum | SUDKH74 | 234 | 181 | 191 | 206 | 176 | 229 | 143 | 294 | 147 | 210 | 222 | 253 | 311 | 178 |
| Sudan | Khartoum | SUDKH75 | 225 | 205 | 194 |  | 185 | 238 | 146 | 258 | 150 | 189 | 210 | 250 | 221 | 196 |
| Sudan | Khartoum | SUDKH76 | 240 | 229 | 191 | 224 | 185 | 244 | 140 | 204 | 153 | 210 | 222 | 277 | 239 | 169 |
| Thailand | NA | THAI1 | 237 | 213 | 188 | 197 | 185 | 238 | 152 | 261 |  | 192 | 228 | 259 | 284 |  |
| Thailand | NA | THAI2 | 237 | 213 | 188 | 197 | 185 | 238 | 155 | 261 | 168 | 192 | 228 | 259 | 284 | 202 |
| Thailand | NA | THAI3 | 237 | 197 | 191 | 197 | 182 | 223 | 155 | 261 | 159 | 189 | 213 |  | 269 | 205 |
| Thailand | NA | THAI4 | 234 | 257 | 200 | 197 | 176 |  | 155 | 276 | 150 |  | 213 | 244 | 254 | 199 |
| Thailand | NA | THAI5 | 237 | 209 | 191 | 203 | 182 | 208 | 143 | 273 | 159 | 195 | 207 | 247 | 275 | 205 |
| Thailand | NA | THAI6 | 231 | 193 | 194 | 197 | 173 |  | 158 | 228 | 168 |  | 207 | 244 | 335 | 211 |
| Thailand | NA | THAI7 | 237 | 197 | 194 | 200 | 182 | 244 | 143 | 255 | 162 | 222 | 216 | 250 | 299 | 220 |
| Thailand | NA | THAI8 | 237 | 177 | 185 | 200 | 185 | 238 | 152 | 243 | 153 | 207 | 210 | 244 | 245 | 205 |
| Thailand | NA | THAI9 | 231 | 237 | 194 | 203 | 218 | 241 | 146 | 231 | 144 | 180 | 207 | 250 | 263 | 208 |
| Thailand | NA | THAI10 | 234 | 205 | 200 |  | 185 | 232 | 173 | 243 | 162 | 195 | 210 | 253 | 248 | 199 |
| Togo | NA | TOGO1 | 234 | 209 | 185 | 194 | 182 | 253 | 143 | 204 | 162 | 183 | 216 | 250 | 248 | 199 |
| Turkey | NA | TQ1 | 234 | 225 | 194 | 194 | 176 | 226 | 146 | 264 | 165 | 216 | 210 | 253 | 305 | 178 |
| Turkey | NA | TQ2 | 234 | 217 | 197 | 200 | 173 | 178 | 143 | 216 | 147 | 171 | 222 | 256 | 260 | 178 |
| Turkey | NA | TQ3 | 237 | 213 | 194 | 209 | 173 | 226 | 143 | 222 | 147 | 210 | 210 | 256 | 350 | 202 |
| Turkey | NA | TQ4 | 237 | 217 | 206 | 194 | 173 | 229 |  | 222 | 147 | 207 | 213 | 253 | 350 | 202 |
| Turkey | NA | TQ5 | 272 | 213 | 185 | 200 | 173 | 226 | 146 | 264 | 147 | 207 | 222 | 256 | 305 | 181 |
| Turkey | NA | TQ6 | 234 | 213 | 188 | 194 | 173 | 238 | 143 | 264 | 144 | 207 | 213 | 250 | 182 | 169 |
| Turkey | NA | TQ7 | 240 | 217 | 188 | 206 | 173 | 232 | 146 | 300 | 174 | 237 | 216 | 268 | 290 | 229 |
| Venezuela | NA | VNZ1 | 234 | 237 | 191 | 200 | 179 | 184 | 152 | 267 | 162 | 234 | 201 | 238 | 278 | 211 |
| Venezuela | NA | VNZ2 | 234 | 237 | 191 | 200 | 179 | 241 | 152 | 267 | 159 | 234 | 201 | 238 | 278 | 211 |
| Venezuela | NA | VNZ3 | 234 | 209 | 191 | 194 | 185 | 181 | 143 | 282 | 168 | 207 | 183 | 268 | 272 | 220 |
| Venezuela | NA | VNZ4 | 234 | 209 | 191 | 212 | 188 | 208 | 155 | 255 | 156 | 195 | 213 | 268 | 338 | 220 |
| Venezuela | NA | VNZ5 | 234 | 237 | 191 | 200 | 188 | 241 | 152 | 267 | 162 | 234 | 210 | 250 | 278 | 211 |
| Venezuela | NA | VNZ6 | 234 | 209 | 191 | 194 | 185 | 211 | 143 | 282 | 168 | 198 | 183 | 268 | 272 | 220 |
| Venezuela | NA | VNZ7 | 237 | 237 | 191 | 200 | 188 | 241 | 152 | 267 | 159 | 234 | 210 | 250 | 278 | 211 |
| Venezuela | NA | VNZ8 | 237 | 217 | 194 | 191 | 179 | 208 | 143 | 288 | 174 | 201 | 234 | 259 | 278 | 205 |
| Venezuela | NA | VNZ9 | 243 | 201 | 194 | 200 | 170 | 184 | 143 | 252 | 165 | 213 | 225 | 241 | 278 | 214 |
| Venezuela | NA | VNZ10 | 237 | 209 | 191 | 200 | 179 | 238 | 155 | 240 | 174 | 216 | 264 | 229 | 338 | 238 |
| Venezuela | NA | VNZ11 | 234 | 233 | 194 | 194 | 176 | 238 | 143 | 243 | 162 | 207 | 210 | 268 | 281 | 259 |
| Venezuela | NA | VNZ12 | 234 | 197 | 191 | 212 | 188 | 229 | 155 | 255 | 165 | 195 | 213 | 241 | 338 | 238 |
| Venezuela | NA | VNZ13 | 234 | 237 | 191 | 212 | 179 | 232 | 155 | 255 | 165 | 195 | 204 | 229 | 338 | 238 |
| Venezuela | NA | VNZ14 | 234 | 209 | 191 | 194 | 185 | 208 | 143 | 282 | 168 | 207 | 183 | 268 | 272 | 220 |
| Venezuela | NA | VNZ15 | 234 | 237 | 191 | 200 | 179 | 241 | 155 | 267 | 162 | 234 | 201 | 238 | 278 | 211 |
| Venezuela | NA | VNZ16 | 234 | 237 | 191 | 200 | 179 | 241 | 152 | 267 | 162 | 234 | 201 | 238 | 281 | 211 |
| Venezuela | NA | VNZ17 | 228 | 197 | 191 | 194 | 179 | 184 | 143 | 240 | 165 | 243 | 183 | 268 | 215 | 232 |
| Venezuela | NA | VNZ18 | 234 | 237 | 191 | 200 | 188 | 187 | 152 | 267 | 162 | 234 | 210 | 250 | 281 | 211 |
| Venezuela | NA | VNZ19 | 234 | 237 | 191 | 200 | 188 | 241 | 155 | 267 | 162 | 234 | 210 | 250 | 281 | 211 |
