# Supplementary material for: Human Plasmodium vivax diversity, population structure and evolutionary origin
Source: PLoS Negl Trop Dis. 2020 Mar 9;14(3):e0008072. doi: 10.1371/journal.pntd.0008072 (PMC7082039; doi:10.1371/journal.pntd.0008072)
Supplement: S4 Table — AFR: Central African Republic + Cameroon + Togo; ARM: Armenia; AZE: Azerbaijan; BAN: Bandarban; BAY: Bay Islands; CAM: Camopi; CAY: Cayenne; COX: Cox’s Bazar; ETH: Ethiopia; HLF: New Halfa; HND: Honduras; IND: India; IRN: Iran; KGR: Khagrachari; KHA: Khartoum; MEX: Mexico; MRT: Mauritania; PAK: Pakistan; PER: Peru; STG: Saint Gorges de l’Oyapock; THA: Thailand; TMY: Thailand/Myanmar; TUR: Turkey; VEN: Venezuela. (DOCX) [file pntd.0008072.s009.docx]

**Table S4.**

|  | MS1 | MS2 | MS4 | MS5 | MS7 | MS8 | MS9 | MS10 | MS12 | MS15 | MS16 | MS20 |  |
| --- | --- | --- | --- | --- | --- | --- | --- | --- | --- | --- | --- | --- | --- |
|  | ASIA | | | | | | | | | | | | |
| THA | 3 | 8 | 3 | 5 | 6 | 7 | 6 | 6 | 5 | 5 | 9 | 6 |  |
| TMY | 3 | 10 | 8 | 9 | 9 | 15 | 8 | 11 | 7 | 8 | 11 | 10 |  |
| IND | 3 | 3 | 2 | 2 | 3 | 4 | 3 | 3 | 4 | 3 | 4 | 3 |  |
| BAN | 4 | 6 | 4 | 7 | 6 | 7 | 5 | 8 | 7 | 6 | 9 | 8 |  |
| KGR | 3 | 8 | 5 | 4 | 6 | 5 | 5 | 7 | 2 | 6 | 7 | 5 |  |
| COX | 4 | 11 | 8 | 10 | 8 | 15 | 9 | 12 | 8 | 10 | 18 | 9 |  |
|  | MIDDLE EAST | | | | | | | | | | | | |
| PAK | 5 | 6 | 7 | 5 | 3 | 13 | 8 | 9 | 7 | 7 | 14 | 12 |  |
| ARM | 3 | 5 | 3 | 6 | 3 | 8 | 6 | 5 | 6 | 6 | 10 | 7 |  |
| AZE | 3 | 3 | 3 | 3 | 4 | 5 | 7 | 4 | 5 | 5 | 5 | 7 |  |
| IRN | 4 | 7 | 5 | 5 | 4 | 11 | 7 | 11 | 11 | 5 | 14 | 8 |  |
| TUR | 3 | 3 | 4 | 2 | 2 | 4 | 4 | 5 | 4 | 4 | 5 | 5 |  |
|  | AFRICA | | | | | | | | | | | | |
| AFR | 1 | 4 | 3 | 3 | 1 | 2 | 2 | 1 | 2 | 2 | 3 | 2 |  |
| ETH | 5 | 5 | 5 | 5 | 2 | 7 | 6 | 8 | 6 | 7 | 6 | 6 |  |
| MRT | 3 | 5 | 4 | 7 | 3 | 11 | 4 | 9 | 7 | 3 | 7 | 9 |  |
| HLF | 4 | 10 | 7 | 8 | 5 | 10 | 7 | 6 | 7 | 6 | 10 | 16 |  |
| KHA | 7 | 14 | 11 | 6 | 5 | 14 | 10 | 6 | 7 | 8 | 15 | 17 |  |
|  | AMERICA | | | | | | | | | | | | |
| MEX | 2 | 6 | 1 | 5 | 4 | 5 | 5 | 8 | 7 | 5 | 6 | 5 |  |
| HND | 8 | 10 | 6 | 5 | 5 | 13 | 7 | 15 | 6 | 7 | 16 | 13 |  |
| BAY | 5 | 5 | 3 | 3 | 3 | 3 | 3 | 5 | 4 | 4 | 4 | 4 |  |
| VEN | 4 | 6 | 4 | 5 | 3 | 7 | 6 | 8 | 8 | 6 | 5 | 7 |  |
| PER | 5 | 8 | 8 | 5 | 4 | 13 | 9 | 12 | 12 | 10 | 21 | 11 |  |
| STG | 3 | 6 | 6 | 10 | 2 | 9 | 6 | 6 | 8 | 7 | 11 | 8 |  |
| CAY | 3 | 6 | 5 | 6 | 3 | 12 | 6 | 8 | 5 | 6 | 8 | 8 |  |
| CAM | 6 | 6 | 5 | 7 | 2 | 6 | 7 | 7 | 6 | 8 | 11 | 9 |  |
