# Supplementary material for: Human Plasmodium vivax diversity, population structure and evolutionary origin
Source: PLoS Negl Trop Dis. 2020 Mar 9;14(3):e0008072. doi: 10.1371/journal.pntd.0008072 (PMC7082039; doi:10.1371/journal.pntd.0008072)
Supplement: S5 Table — ARM: Armenia; AZE: Azerbaijan; BAN: Bandarban; BAY: Bay Islands; CAM: Camopi; CAY: Cayenne; COX: Cox’s Bazar; ETH: Ethiopia; HLF: New Halfa; HND: Honduras; IND: India; IRN: Iran; KGR: Khagrachari; KHA: Khartoum; MEX: Mexico; MRT: Mauritania; PAK: Pakistan; PER: Peru; STG: Saint Gorges de l’Oyapock; THA: Thailand; TMY: Thailand/Myanmar; TUR: Turkey; VEN: Venezuela. (DOCX) [file pntd.0008072.s010.docx]

**Table S5.**

|  | **MS1** | **MS2** | **MS4** | **MS5** | **MS7** | **MS8** | **MS9** | **MS10** | **MS12** | **MS15** | **MS16** | **MS20** | **Average** |
| --- | --- | --- | --- | --- | --- | --- | --- | --- | --- | --- | --- | --- | --- |
| ASIA | | | | | | | | | | | | | |
| **THA** | 0.622 | 0.956 | 0.667 | 0.800 | 0.889 | 0.911 | 0.917 | 0.929 | 0.867 | 0.861 | 0.978 | 0.889 | 0.857 |
| **TMY** | 0.658 | 0.910 | 0.866 | 0.823 | 0.861 | 0.967 | 0.827 | 0.931 | 0.827 | 0.840 | 0.928 | 0.909 | 0.862 |
| **IND** | 0.833 | 0.833 | 1.000 | 0.667 | 0.833 | 1.000 | 0.833 | 0.833 | 1.000 | 0.833 | 1.000 | 0.833 | 0.875 |
| **BAN** | 0.782 | 0.833 | 0.800 | 0.909 | 0.782 | 0.846 | 0.756 | 0.923 | 0.894 | 0.859 | 0.936 | 0.897 | 0.851 |
| **KGR** | 0.464 | 1.000 | 0.857 | 0.821 | 0.893 | 0.857 | 0.893 | 0.964 | 0.536 | 0.929 | 0.964 | 0.857 | 0.836 |
| **COX** | 0.715 | 0.938 | 0.858 | 0.889 | 0.870 | 0.957 | 0.877 | 0.939 | 0.759 | 0.889 | 0.976 | 0.905 | 0.881 |
| MIDDLE EAST | | | | | | | | | | | | | |
| **PAK** | 0.670 | 0.675 | 0.849 | 0.707 | 0.632 | 0.943 | 0.866 | 0.840 | 0.807 | 0.869 | 0.938 | 0.934 | 0.811 |
| **ARM** | 0.410 | 0.771 | 0.292 | 0.710 | 0.581 | 0.837 | 0.776 | 0.748 | 0.789 | 0.813 | 0.843 | 0.786 | 0.696 |
| **AZE** | 0.495 | 0.267 | 0.338 | 0.452 | 0.586 | 0.700 | 0.790 | 0.629 | 0.552 | 0.595 | 0.581 | 0.562 | 0.546 |
| **IRN** | 0.684 | 0.831 | 0.750 | 0.507 | 0.500 | 0.941 | 0.875 | 0.958 | 0.926 | 0.676 | 0.990 | 0.882 | 0.793 |
| **TUR** | 0.714 | 0.714 | 0.810 | 0.286 | 0.600 | 0.810 | 0.714 | 0.857 | 0.857 | 0.810 | 0.905 | 0.905 | 0.749 |
| AFRICA | | | | | | | | | | | | | |
| **AFR** | 0.000 | 1.000 | 0.833 | 0.833 | 0.000 | 0.500 | 0.500 | 0.000 | 1.000 | 0.500 | 0.833 | 0.500 | 0.541 |
| **ETH** | 0.737 | 0.790 | 0.660 | 0.710 | 0.344 | 0.742 | 0.703 | 0.775 | 0.620 | 0.713 | 0.537 | 0.675 | 0.667 |
| **MRT** | 0.545 | 0.591 | 0.663 | 0.697 | 0.239 | 0.870 | 0.674 | 0.687 | 0.833 | 0.387 | 0.533 | 0.800 | 0.627 |
| **HLF** | 0.778 | 0.928 | 0.781 | 0.842 | 0.765 | 0.962 | 0.837 | 0.797 | 0.843 | 0.817 | 0.935 | 0.977 | 0.855 |
| **KHA** | 0.769 | 0.861 | 0.784 | 0.773 | 0.686 | 0.868 | 0.796 | 0.748 | 0.649 | 0.699 | 0.870 | 0.838 | 0.778 |
| AMERICA | | | | | | | | | | | | | |
| **MEX** | 0.331 | 0.547 | 0.000 | 0.453 | 0.582 | 0.618 | 0.680 | 0.772 | 0.697 | 0.692 | 0.685 | 0.731 | 0.566 |
| **HND** | 0.828 | 0.794 | 0.590 | 0.677 | 0.557 | 0.929 | 0.838 | 0.911 | 0.637 | 0.830 | 0.954 | 0.897 | 0.787 |
| **BAY** | 0.861 | 0.806 | 0.667 | 0.607 | 0.722 | 0.679 | 0.667 | 0.722 | 0.643 | 0.750 | 0.714 | 0.583 | 0.702 |
| **VEN** | 0.450 | 0.725 | 0.661 | 0.731 | 0.702 | 0.795 | 0.813 | 0.801 | 0.865 | 0.830 | 0.789 | 0.784 | 0.746 |
| **PER** | 0.574 | 0.817 | 0.764 | 0.545 | 0.261 | 0.832 | 0.829 | 0.662 | 0.862 | 0.825 | 0.942 | 0.842 | 0.730 |
| **STG** | 0.434 | 0.616 | 0.599 | 0.707 | 0.173 | 0.828 | 0.710 | 0.684 | 0.663 | 0.661 | 0.622 | 0.639 | 0.611 |
| **CAY** | 0.397 | 0.783 | 0.736 | 0.794 | 0.372 | 0.923 | 0.817 | 0.855 | 0.779 | 0.663 | 0.842 | 0.790 | 0.729 |
| **CAM** | 0.801 | 0.768 | 0.747 | 0.774 | 0.505 | 0.853 | 0.768 | 0.784 | 0.804 | 0.883 | 0.974 | 0.795 | 0.788 |
