# Supplementary material for: Human Plasmodium vivax diversity, population structure and evolutionary origin
Source: PLoS Negl Trop Dis. 2020 Mar 9;14(3):e0008072. doi: 10.1371/journal.pntd.0008072 (PMC7082039; doi:10.1371/journal.pntd.0008072)
Supplement: S6 Table — *P-value < 0.008 (Bonferroni correction). (DOCX) [file pntd.0008072.s011.docx]

**Table S6.**

|  | America | Africa | Asia | Middle East |
| --- | --- | --- | --- | --- |
| America | 0 | 0.160 (0.074) | 0.147 (0.152) | 0.407 (0.000)* |
| Africa |  | 0 | 0.139 (0.196) | 0.294 (0.054) |
| Asia |  |  | 0 | 0.476 (0.018) |
| Middle East |  |  |  | 0 |
