# Supplementary material for: Human Plasmodium vivax diversity, population structure and evolutionary origin
Source: PLoS Negl Trop Dis. 2020 Mar 9;14(3):e0008072. doi: 10.1371/journal.pntd.0008072 (PMC7082039; doi:10.1371/journal.pntd.0008072)
Supplement: S7 Table — All significant values are indicated in bold (p-value < 0.05). AFR: Cantral African Republic + Cameroon + Togo; ARM: Armenia; AZE: Azerbaijan; BAN: Bandabar; BAY: Bay Islands; CAM: Camopi; CAY: Cayenne; COX: Cox’s Bazar; ETH: Ethiopia; HLF: New Halfa; HND: Honduras; IND: India; IRN: Iran; KGR: Khagrachari; KHA: Khartoum; MEX: Mexico; MRT: Mauritania; PAK: Pakistan; PER: Peru; STG: Saint Georges de l’Oyapock; THA: Thailand; TMY: Thailand/Myanmar; TUR: Turkey; VEN: Venezuela. (DOCX) [file pntd.0008072.s012.docx]

**Table S7.**

|  | **AFR** | **ARM** | **AZE** | **BAN** | **BAY** | **CAM** | **CAY** | **COX** | **ETH** | **HLF** | **HND** | **IND** | **IRN** | **KGR** | **KHA** | **MEX** | **MRT** | **PAK** | **PER** | **STG** | **THA** | **TMY** | **TUR** | **VEN** |
| --- | --- | --- | --- | --- | --- | --- | --- | --- | --- | --- | --- | --- | --- | --- | --- | --- | --- | --- | --- | --- | --- | --- | --- | --- |
| **AFR** | 0 | 0.219 | 0.369 | 0.177 | 0.276 | 0.172 | 0.233 | 0.112 | 0.265 | 0.074 | 0.175 | 0.142 | 0.095 | 0.103 | 0.126 | 0.279 | 0.196 | 0.101 | 0.168 | 0.299 | 0.174 | 0.106 | 0.197 | 0.199 |
| **ARM** |  | 0 | 0.08 | 0.171 | 0.222 | 0.163 | 0.172 | 0.127 | 0.203 | 0.116 | 0.171 | 0.097 | 0.107 | 0.144 | 0.162 | 0.3 | 0.215 | 0.11 | 0.14 | 0.25 | 0.168 | 0.132 | 0.113 | 0.182 |
| **AZE** |  |  | 0 | 0.261 | 0.347 | 0.259 | 0.284 | 0.212 | 0.34 | 0.228 | 0.264 | 0.236 | 0.236 | 0.268 | 0.253 | 0.383 | 0.314 | 0.213 | 0.253 | 0.338 | 0.272 | 0.232 | 0.261 | 0.286 |
| **BAN** |  |  |  | 0 | 0.151 | 0.082 | 0.131 | **0.023** | 0.162 | 0.075 | 0.103 | **0.048** | 0.087 | **0.042** | 0.103 | 0.229 | 0.197 | 0.079 | 0.127 | 0.204 | **0.031** | **0.038** | 0.117 | 0.101 |
| **BAY** |  |  |  |  | 0 | 0.134 | 0.165 | 0.121 | 0.201 | 0.146 | 0.077 | 0.178 | 0.157 | 0.168 | 0.178 | 0.266 | 0.269 | 0.169 | 0.199 | 0.26 | 0.133 | 0.13 | 0.207 | 0.192 |
| **CAM** |  |  |  |  |  | 0 | 0.064 | 0.064 | 0.183 | 0.088 | 0.113 | 0.091 | 0.104 | 0.087 | 0.138 | 0.247 | 0.191 | 0.118 | 0.134 | 0.145 | 0.056 | 0.070 | 0.135 | 0.113 |
| **CAY** |  |  |  |  |  |  | 0 | 0.095 | 0.197 | 0.122 | 0.135 | 0.139 | 0.129 | 0.124 | 0.159 | 0.295 | 0.23 | 0.133 | 0.143 | 0.113 | 0.09 | 0.097 | 0.143 | 0.147 |
| **COX** |  |  |  |  |  |  |  | 0 | 0.142 | **0.025** | 0.083 | **0.023** | 0.06 | **0.016** | 0.073 | 0.216 | 0.166 | 0.054 | 0.104 | 0.159 | **0.009** | **0.01** | 0.078 | 0.087 |
| **ETH** |  |  |  |  |  |  |  |  | 0 | 0.13 | 0.15 | 0.171 | 0.146 | 0.162 | 0.161 | 0.305 | 0.254 | 0.147 | 0.175 | 0.239 | 0.163 | 0.147 | 0.2 | 0.219 |
| **HLF** |  |  |  |  |  |  |  |  |  | 0 | 0.103 | **0.016** | 0.054 | **0.043** | **0.042** | 0.226 | 0.164 | **0.039** | 0.118 | 0.19 | 0.058 | **0.039** | 0.066 | 0.132 |
| **HND** |  |  |  |  |  |  |  |  |  |  | 0 | 0.071 | 0.098 | 0.102 | 0.132 | 0.168 | 0.177 | 0.097 | 0.121 | 0.185 | 0.093 | 0.082 | 0.124 | 0.118 |
| **IND** |  |  |  |  |  |  |  |  |  |  |  | 0 | **0** | **0.041** | 0.072 | 0.212 | 0.137 | **0** | 0.098 | 0.221 | **0.008** | **0** | **0.032** | 0.127 |
| **IRN** |  |  |  |  |  |  |  |  |  |  |  |  | 0 | **0.047** | 0.103 | 0.185 | 0.117 | **0.024** | 0.077 | 0.197 | 0.079 | **0.029** | **0.045** | 0.116 |
| **KGR** |  |  |  |  |  |  |  |  |  |  |  |  |  | 0 | 0.085 | 0.195 | 0.165 | 0.056 | 0.095 | 0.201 | 0.068 | **0.009** | 0.083 | 0.081 |
| **KHA** |  |  |  |  |  |  |  |  |  |  |  |  |  |  | 0 | 0.233 | 0.193 | 0.098 | 0.146 | 0.212 | 0.113 | 0.082 | 0.141 | 0.156 |
| **MEX** |  |  |  |  |  |  |  |  |  |  |  |  |  |  |  | 0 | 0.234 | 0.208 | 0.223 | 0.346 | 0.236 | 0.18 | 0.252 | 0.23 |
| **MRT** |  |  |  |  |  |  |  |  |  |  |  |  |  |  |  |  | 0 | 0.16 | 0.16 | 0.255 | 0.171 | 0.143 | 0.22 | 0.191 |
| **PAK** |  |  |  |  |  |  |  |  |  |  |  |  |  |  |  |  |  | 0 | 0.101 | 0.202 | 0.071 | 0.052 | 0.082 | 0.121 |
| **PER** |  |  |  |  |  |  |  |  |  |  |  |  |  |  |  |  |  |  | 0 | 0.168 | 0.114 | 0.092 | 0.144 | 0.132 |
| **STG** |  |  |  |  |  |  |  |  |  |  |  |  |  |  |  |  |  |  |  | 0 | 0.136 | 0.171 | 0.238 | 0.212 |
| **THA** |  |  |  |  |  |  |  |  |  |  |  |  |  |  |  |  |  |  |  |  | 0 | **0.022** | 0.117 | 0.115 |
| **TMY** |  |  |  |  |  |  |  |  |  |  |  |  |  |  |  |  |  |  |  |  |  | 0 | 0.068 | 0.093 |
| **TUR** |  |  |  |  |  |  |  |  |  |  |  |  |  |  |  |  |  |  |  |  |  |  | 0 | 0.173 |
| **VEN** |  |  |  |  |  |  |  |  |  |  |  |  |  |  |  |  |  |  |  |  |  |  |  | 0 |
